# Object-oriented hand dexterity and grasping abilities, from the animal quarters to the neurosurgical OR: a systematic review of the underlying neural correlates in non-human, human primate and recent findings in awake brain surgery

**DOI:** 10.3389/fnint.2024.1324581

**Published:** 2024-02-15

**Authors:** Leonardo Tariciotti, Luca Mattioli, Luca Viganò, Matteo Gallo, Matteo Gambaretti, Tommaso Sciortino, Lorenzo Gay, Marco Conti Nibali, Alberto Gallotti, Gabriella Cerri, Lorenzo Bello, Marco Rossi

**Affiliations:** ^1^Neurosurgical Oncology Unit, Department of Oncology and Hemato-Oncology, Università degli Studi di Milano, Milan, Italy; ^2^MoCA Laboratory, Department of Medical Biotechnology and Translational Medicine, Università degli Studi di Milano, Milan, Italy; ^3^Neurosurgical Oncology Unit, Department of Medical Biotechnology and Translational Medicine, Università degli Studi di Milano, Milan, Italy

**Keywords:** grasping network, object-oriented hand manipulation, hand manipulation, motor cognition, brain mapping, awake surgery, brain tumor

## Abstract

**Introduction:**

The sensorimotor integrations subserving object-oriented manipulative actions have been extensively investigated in non-human primates via direct approaches, as intracortical micro-stimulation (ICMS), cytoarchitectonic analysis and anatomical tracers. However, the understanding of the mechanisms underlying complex motor behaviors is yet to be fully integrated in brain mapping paradigms and the consistency of these findings with intraoperative data obtained during awake neurosurgical procedures for brain tumor removal is still largely unexplored. Accordingly, there is a paucity of systematic studies reviewing the cross-species analogies in neural activities during object-oriented hand motor tasks in primates and investigating the concordance with intraoperative findings during brain mapping. The current systematic review was designed to summarize the cortical and subcortical neural correlates of object-oriented fine hand actions, as revealed by fMRI and PET studies, in non-human and human primates and how those were translated into neurosurgical studies testing dexterous hand-movements during intraoperative brain mapping.

**Methods:**

A systematic literature review was conducted following the PRISMA guidelines. PubMed, EMBASE and Web of Science databases were searched. Original articles were included if they: (1) investigated cortical activation sites on fMRI and/or PET during grasping task; (2) included humans or non-human primates. A second query was designed on the databases above to collect studies reporting motor, hand manipulation and dexterity tasks for intraoperative brain mapping in patients undergoing awake brain surgery for any condition. Due to the heterogeneity in neurosurgical applications, a qualitative synthesis was deemed more appropriate.

**Results:**

We provided an updated overview of the current state of the art in translational neuroscience about the extended frontoparietal grasping-praxis network with a specific focus on the comparative functioning in non-human primates, healthy humans and how the latter knowledge has been implemented in the neurosurgical operating room during brain tumor resection.

**Discussion:**

The anatomical and functional correlates we reviewed confirmed the evolutionary continuum from monkeys to humans, allowing a cautious but practical adoption of such evidence in intraoperative brain mapping protocols. Integrating the previous results in the surgical practice helps preserve complex motor abilities, prevent long-term disability and poor quality of life and allow the maximal safe resection of intrinsic brain tumors.

## 1 Introduction

Dexterous, effortless and reproducible hand movements represent a determinant feature of human behavior: they allow interaction with the surrounding environment, manipulate and craft objects and tools, generate complex non-verbal forms of communication and satisfy indispensable needs according to external contingencies. Since the first experiments in the early twentieth century on direct cortical stimulation, investigations on the anatomical and functional substrates of pure motor responses first and complex, meaningful hand motor actions later have shown a growing trend with an intensification in the last three decades, fostered by the expanding number of available invasive and non-invasive study modalities. The former, mostly in non-human primates, clarified that motor behavior control lies on the cortico-subcortical sensory and motor input integration throughout reciprocal modulations of primary motor, parieto-premotor and cortico-thalamic loops (Alexander and Crutcher, 1990; Rizzolatti et al., 1998).

In monkeys, previous intracortical micro-stimulation (ICMS), cytoarchitectonic and functional imaging studies clarified the involvement of a frontoparietal system connecting areas of the inferior parietal lobule (IPL) and the ventral premotor (PMv) cortex in the selection process and online control of purposeful goal-oriented hand actions (Belmalih et al., 2009; Gerbella et al., 2011; Maeda et al., 2015; Borra et al., 2017) This large-scale network, centered on vPM and extended to prefrontal and temporal areas, has been defined as *lateral grasping network* and is supposed to shape the motor output integrating sensorimotor information with higher order inputs as action goals and object’s features derived from the context or retrieved from memory (Borra et al., 2017; Borra and Luppino, 2019). Similarly, consistent data from healthy human subjects showed a homolog topography comprehending areas within the supramarginal gyrus (SMG), the angular gyrus (AG), intraparietal sulcus and the ventral premotor cortex (vPM) at the core of a vast network encoding objects properties, contextual information and behavioral adaptation schemes for generating complex hand-limb motor actions (Jeannerod et al., 1995; Rosenzopf et al., 2022; Sartin et al., 2022). Converging findings from different study modalities agree that non-human and human primates might share a common functional architecture subserving goal-directed actions comprehending prehension and manipulation of objects and tools (Orban and Caruana, 2014). In both species, an analog duality in dorsal frontoparietal connectivity (i.e., a dorsomedial pathway specialized in the visuo-motor integration for reaching and limb lifting and dorso-ventral pathway encoding sensorimotor integration and more direct access to motor output through parieto-premotor and premotor-motor projections) has been identified, segregating the control of specific and complementary features of hand motor schemes (Geschwind, 1975; Goldenberg, 2009; Grafton, 2010; Nelissen and Vanduffel, 2011; Glover et al., 2012; Caminiti et al., 2015). The evolutionary gain in humans led to an expansion of multimodal areas in the frontal, temporal and parietal lobes, probably due to an evolutionary-guided alteration of the macaque’s pre-existing dual-stream frontoparietal network areas. In this view, this modification allowed more complex behavioral responses—which are not affordable for non-human primates—to be encoded (i.e., praxis abilities, complex communicative limb gestures and abstract manipulative tasks; de Waal and Ferrari, 2010; Van Essen et al., 2016; Dressing et al., 2018). Accordingly, fMRI studies in humans described a wider fronto-temporo-parietal network, defined as “the praxis representation network” (PRN), consistently involved in elaborating conceptual and sensorial knowledge into goal-directed and specialized hand motor actions (Frey, 2008; Króliczak and Frey, 2009).

The extensive knowledge about neural correlates of motor functioning has been successfully translated from animal studies to intraoperative brain mapping protocols developed to guide oncological and epilepsy neurosurgery. This intimate relation has been indissoluble since Penfield and colleagues’ first experiments on direct electrical stimulation (DES) of the human cortex eliciting motor responses, influenced by previous pioneering animal experiments Penfield witnessed and collaborated on under Sherrington’s guide in the early 20th century. Since then, animal studies have inspired and provided a solid background to many brain mapping studies in intraoperative scenarios.

Awake surgery employing DES is routinely performed in specialized centers to preserve cortical and subcortical essential components of the motor network to maximally extend the resection, preserving patient’s motor abilities (so-called “onco-functional balance”; Duffau and Mandonnet, 2013). Despite several advancements, a comprehensive exploration of the mechanisms underlying object-oriented dexterous hand movements in pre- and clinical scenarios remains a relevant challenge. The old-world monkeys, like macaques, represent the closest ancestor of *Homo Sapiens* in which, through invasive neurophysiological and anatomical studies, detailed anatomical and physiological notions on the neural bases of sensorimotor and high-domain cognitive functioning can be carried out and generalized to human models. However, despite undoubted similarities between species, the about 30 million years of independent evolution drove significant differences in brain architecture and function, contributing to the peculiar cognitive capabilities of humans but also entangling complete transpositions of evidence from one species to the other. In addition, invasive methods with high temporal and spatial resolutions constituting the gold standard for formulating causal inference in neural mechanisms are not reproducible—for obvious reasons – in humans.

FMRI applications have been developed to fill this gap in humans; to map specific areas, activated voxels scans and analyses of time-dependent regional activities can be obtained first, then the relation of such areas to the specific function elicited can be indirectly derived with reduced spatial and temporal resolutions compared to the methods above developed in monkeys (for additional details see Hillman, 2014).

Although the translational impact of these neural substrates on the neurosurgical practice is indisputable, the anatomo-functional consistency between preclinical data gathered on primates and the results obtained in neurosurgical settings with brain mapping protocols has not yet been fully analyzed and discussed. A systematic review of this topic is a critical step in shedding light on the influence of cross-species basic science on neurosurgical practice, directly impacting patients’ quality of life and survival expectations. The current study aims to systematically collect evidence on the neural substrates of object-oriented hand manipulation movements in healthy non- and human primates and from intraoperative studies investigating responses after direct electrical stimulation of hand movement-related cortical areas and subcortical structures.

## 2 Methods

### 2.1 Information sources and search strategy

A systematic literature review was conducted according to the Cochrane Handbook for Systematic Reviews and was reported based on the PRISMA statement for reporting systematic reviews and meta-analyses (Page et al., 2021). The aim of the current study was clarified through the definition of the following questions:

- Query 1 (*Non-human primate and human grasping investigations*; from here referred to as Q1): *What is the evidence on the anatomical-functional substrate of objects/tools grasping and hand manipulation skills in non-human primates and healthy humans? What are the homologies and differences between species?*
- Query 2 (*Intraoperative awake grasping and hand manipulation tasks investigations*: from here referred to as Q2): *What implication have the evidence collected from non-human primates and healthy humans in designing and implementing intraoperative advanced brain mapping paradigms to preserve grasping capacity, hand dexterity and fine motor abilities during awake surgeries*? *What is the state of the art on the intraoperative mapping and monitoring of grasping and/or any additional fine hand motor task during awake brain surgery?*

A systematic literature search was conducted in three biomedical databases: (i) PubMed, (ii) EMBASE, (iii) Web of Science. The search was updated to 28 February 2023 and further updated on 15 September 2023). To the best of our knowledge, no additional study published after this date and available through a literature search in the databases reported above could be included in our study.

### 2.2 Inclusion criteria

#### 2.2.1 Query 1

**Figure 1:**
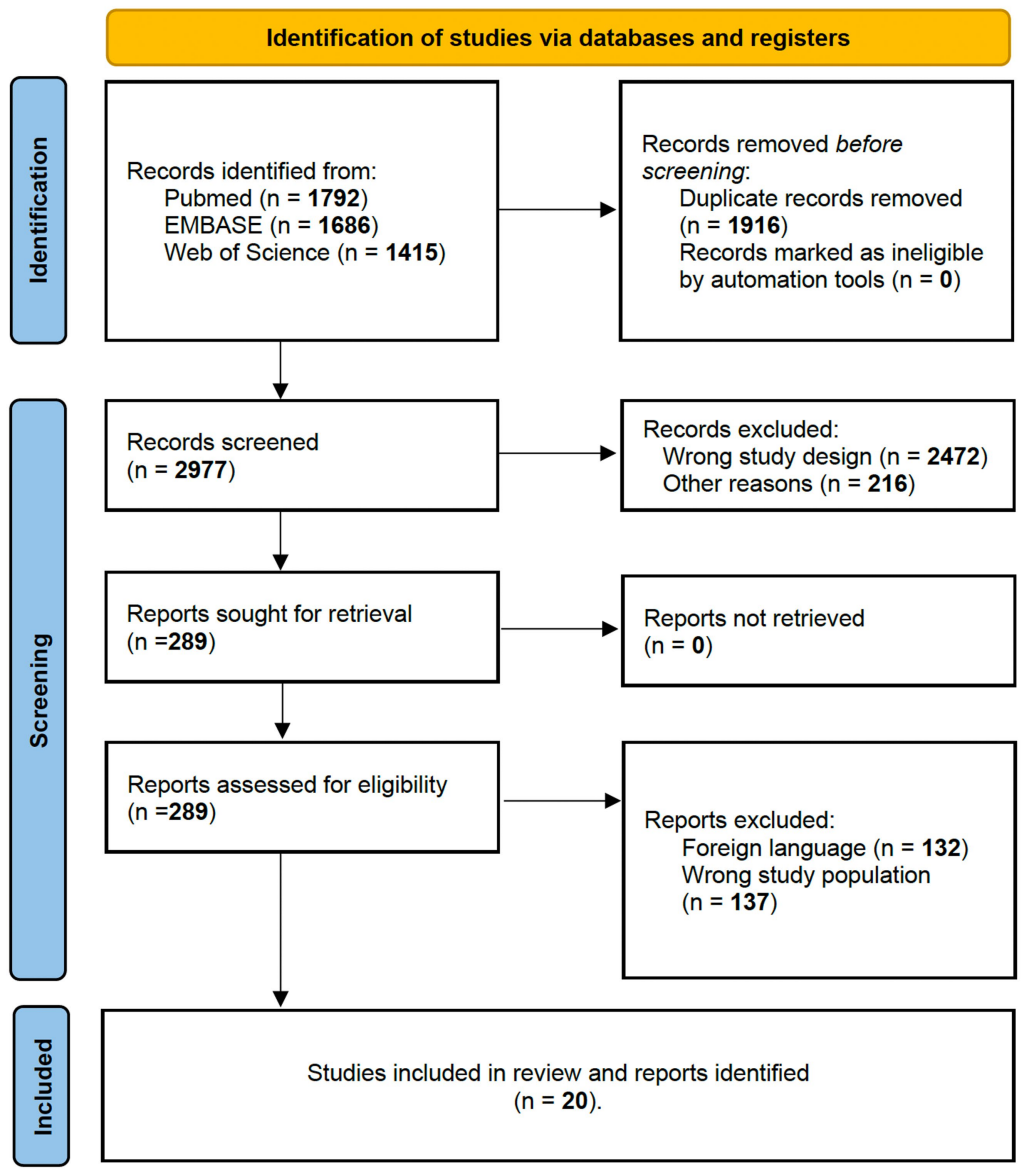
PRISMA 2020 flow diagram for studies involving non-human primates and healthy human participants investigating brain activated region during grasp-related fine hand gestures.

For Query 1 (Q1), the following PICO terms were used: “*(Grasping) AND ((fMRI) OR (MRI) OR (functional MRI) OR (PET))*.” All studies had to respect the following inclusion criteria to be considered in our systematic review (Figure 1):

- To report an experimental investigation on non-human primates or healthy adult human candidates with a sample size equal to or greater than two participants (we excluded single case reports given the poor level of evidence provided);
- To test a hand grasping, reach-and-grasp or hand manipulation task reporting a contrast showing more significant activation levels for the execution task than a control condition. Control conditions include passive view, reach, simple finger movements, object detection and object discrimination);
- To declare a measurement of brain activity during the active execution of the task mentioned above as the study’s primary outcome. Comparative assessment of brain activity during planning or passive tasks was reported as secondary findings if the study’s primary goal fit all inclusion criteria. Studies focused on brain activity during resting or planning phases were excluded;
- To use fMRI or PET to measure neural activity indirectly;
- To have conducted a ROI-base or whole-brain analysis;
- To have performed a univariate, a multivoxel pattern (MVPA), or a functional connectivity analysis (aiming to qualitatively summarize the body of literature irrespectively of the nature of data provided, a consensus among authors was achieved for interpreting the impact of results expressed through different types of analyses; L.T., L.M., L.V., M.R.);
- To report activation areas in Montreal Neurologic Institute (MNI) or Talairach coordinate spaces (TAL); studies reporting findings in native space were excluded.

#### 2.2.2 Query 2

**Figure 2:**
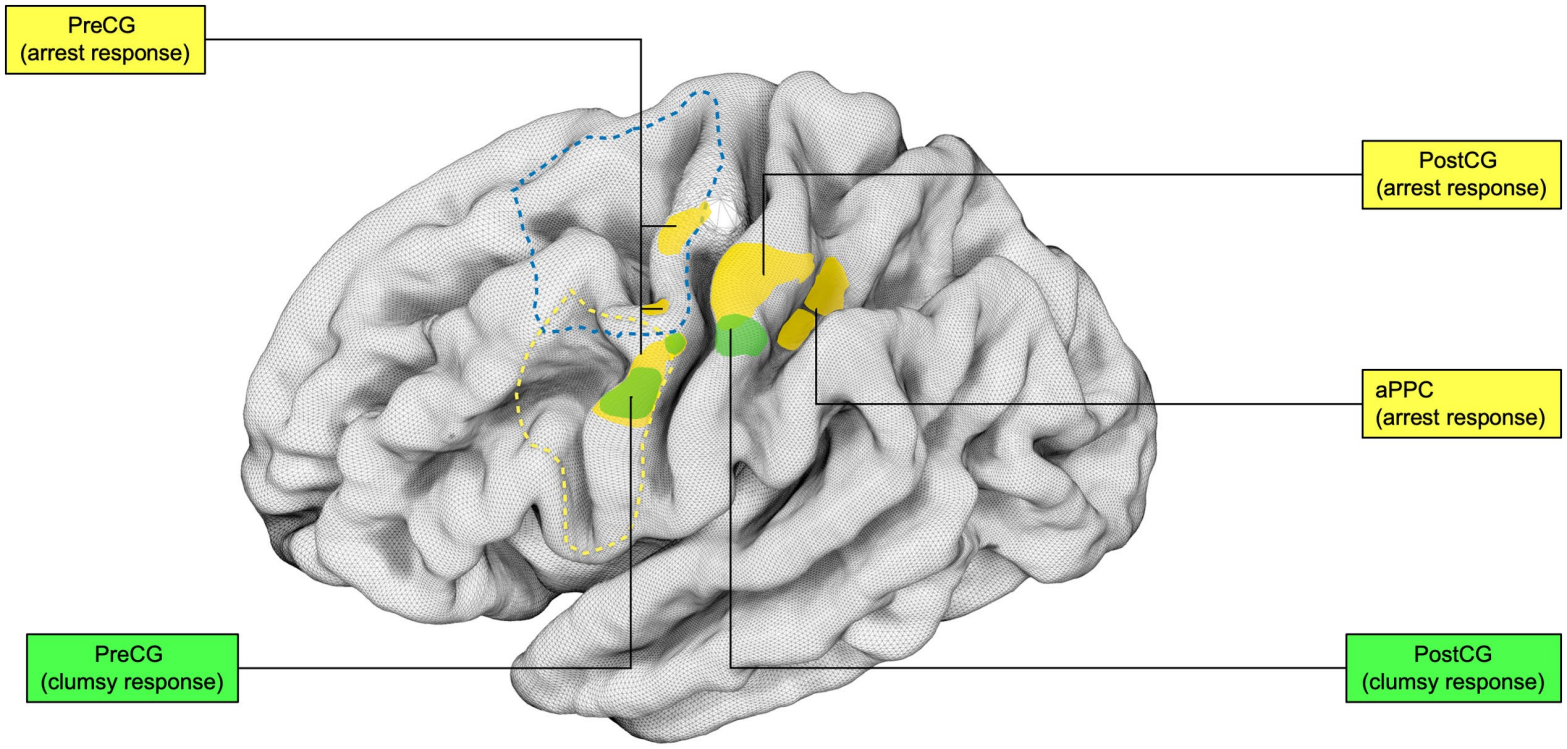
PRISMA 2020 flow diagram for intraoperative awake surgery brain mapping and monitoring of grasping and fine hand manipulation tasks.

For Query 2 (Q2), the following PICO terms were used: *“((Dexterity) OR (grasping) OR (Fine motor) OR (Grip) OR (Haptic) OR (Hand manipulation) OR (manipulation) OR (Hand movement) OR (praxis) OR (apraxia) OR (sensorimotor network) OR (motor network)) AND ((Intraoperative monitoring) OR (IOM) OR (Direct Electrical Stimulation) OR (DES) OR (awake) OR (Intraoperative Mapping)).”* All studies had to respect the following inclusion criteria to be considered in our systematic review (Figure 2):

- To report an experimental study investigating any intraoperative assessment of grasping, reach-and-grasp ability or other fine hand movement tasks (i.e., *dexterity, haptic-related fine finger movements, precision grip, pinching and whole-hand power grip*).
- To test the functions mentioned earlier in human patients undergoing awake surgery for any condition (i.e., intra-axial tumors, vascular lesions in so-called “eloquent areas” or epilepsy surgery) employing brain mapping with or without intraoperative neurophysiological monitoring (IOM).
- To report surgical outcomes regarding function preservation, functional independence after surgery, quality of life and/or extent of resection.

### 2.3 Exclusion criteria

The following exclusion criteria were applied (both Q1 and Q2):

- *Publication design:* Case reports, abstracts, commentaries, editorial papers, conference papers and publications written in any language but English were excluded
- *Contents:* all studies not fulfilling the aforementioned inclusion criteria were excluded.

### 2.4 Selection process

Two authors (L.T. and L.M.) independently reviewed the titles and abstracts of the retrieved articles, classifying them as included, excluded and maybe. During this stage, the articles that did not meet the inclusion criteria were excluded (such as reports written not in English, studies on non-primate animals, *in vitro* studies, abstracts, reviews, commentaries and case reports). In case of disagreement between the authors, the consensus was reached by full-text jointly-conducted examination. Afterwards, the full texts of the articles classified as “included” and “maybe” were independently assessed by the same authors (L.T. and L.M.). Again, in case of disagreement, the consensus was reached by broad discussion with the senior author (M.R.). No automatic tools were employed during the selection process.

### 2.5 Review of reports

Due to the heterogeneity in the body of evidence collected for Q1 and Q2, quantitative synthesis was deemed inappropriate. For Q1, a qualitative synthesis of results was provided: results collected from the reviewed articles were compiled through a narrative approach, and an updated description of the anatomical and functional bases of grasping and fine hand motor tasks characteristics and functional implications in non-human primates and healthy humans was provided. Similarly, for Q2, a synthesis of findings will be provided: the state-of-the-art intraoperative neurophysiological monitoring and brain mapping paradigms for hand fine movements/grasping-related tasks preservation during awake surgeries were presented in a narrative form.

Overall, to improve the readability of our qualitative review, major evidence from selected studies will be organized according to their anatomical location and connectivity, following cortical and cortico-subcortical topographical segregation. We are aware this approach will penalize the characterization and discussion of specific studies and their methodological peculiarities; however, we aimed to provide a physician-oriented comprehensive review of the most relevant cortical and subcortical nodes involved in the mechanisms under investigation with a potential impact on the neurosurgical research and clinical practice.

A detailed methodological revision of study designs and their implications was beyond the scope of the current study.

### 2.6 Graphical contents

The relevant sectors of the lateral grasping network in monkeys and object-oriented hand dexterity network / praxis representative network in humans were collected and reproduced in tridimensional standardized left-hemisphere brain maps. In Monkeys, lateral grasping network sectors, comprehending the exploratory oculomotor network, were drawn on the Mount Sinai cohort data on the INIA-19 template,^1^ as previously done by Howells and colleagues (for additional methodological insights, see Rohlfing et al., 2012; Howells et al., 2020). Similarly, the relevant areas in humans were extracted by the Human Connectome Project atlas, multi-modal cortical parcellation (HCP-MMP1.0; Glasser et al., 2016). Intraoperative Data shown were extracted from Vigano et al. and Fornia et al. and rendered within the left hemisphere only (Fornia et al., 2020a, 2023). The figures in the article were designed and produced within the open-source surface render software “SurfIce” (SurfIce, 2015).

## 3 Results

### 3.1 Imaging-based experimental findings

For Q1, bibliographic searches on literature databases yielded 3,611 records (PubMed: 2,038; Embase:783; Web of Science: 790). After removing duplicates (971 records) and unrelated manuscripts, 598 were selected for full-text evaluation. Among these, 528 were further excluded, as they did not meet the predefined inclusion criteria. Additional 13 studies were extracted from appropriate references during the screening. Overall, 85 records were included in our systematic review: six on non-human primates and 79 on healthy human candidates. Figure 1 shows the flow diagram of the literature search and study selection.

#### 3.1.1 Non-human primates

Six studies (Nishimura et al., 2007; Hopkins et al., 2010; Nelissen and Vanduffel, 2011; Hecht et al., 2013; Fiave et al., 2018; Nelissen et al., 2018) on non-human primates were completed between 2007 and 2018 and included overall nine macaques and 78 chimpanzees [in Hopkins et al., 2010, only four chimpanzees underwent the behavioral task during PET scanning; 70 chimpanzees were recruited for baseline MRI scan only and further segmentation of hand knob region]. Five studies explored activation areas on the whole brain surface, while Fiave et al. focused only on the left hemisphere. Four studies conducted grasp and reach-and-grasp experiments with (Nishimura et al., 2007; Hopkins et al., 2010) or without visual aid (i.e., “grasping in the dark”; Nelissen and Vanduffel, 2011; Hecht et al., 2013; Fiave et al., 2018; Nelissen et al., 2018). All studies but Hopkins et al. allowed right hand movements only.

Three investigations used an FDG-PET imaging acquisition and further co-registration in MRI-normalized coordinates (Nishimura et al., 2007; Hopkins et al., 2010; Hecht et al., 2013), while the other three studies performed the behavioral task during fMRI (Nelissen and Vanduffel, 2011; Fiave et al., 2018; Nelissen et al., 2018).

**Figure 3:**
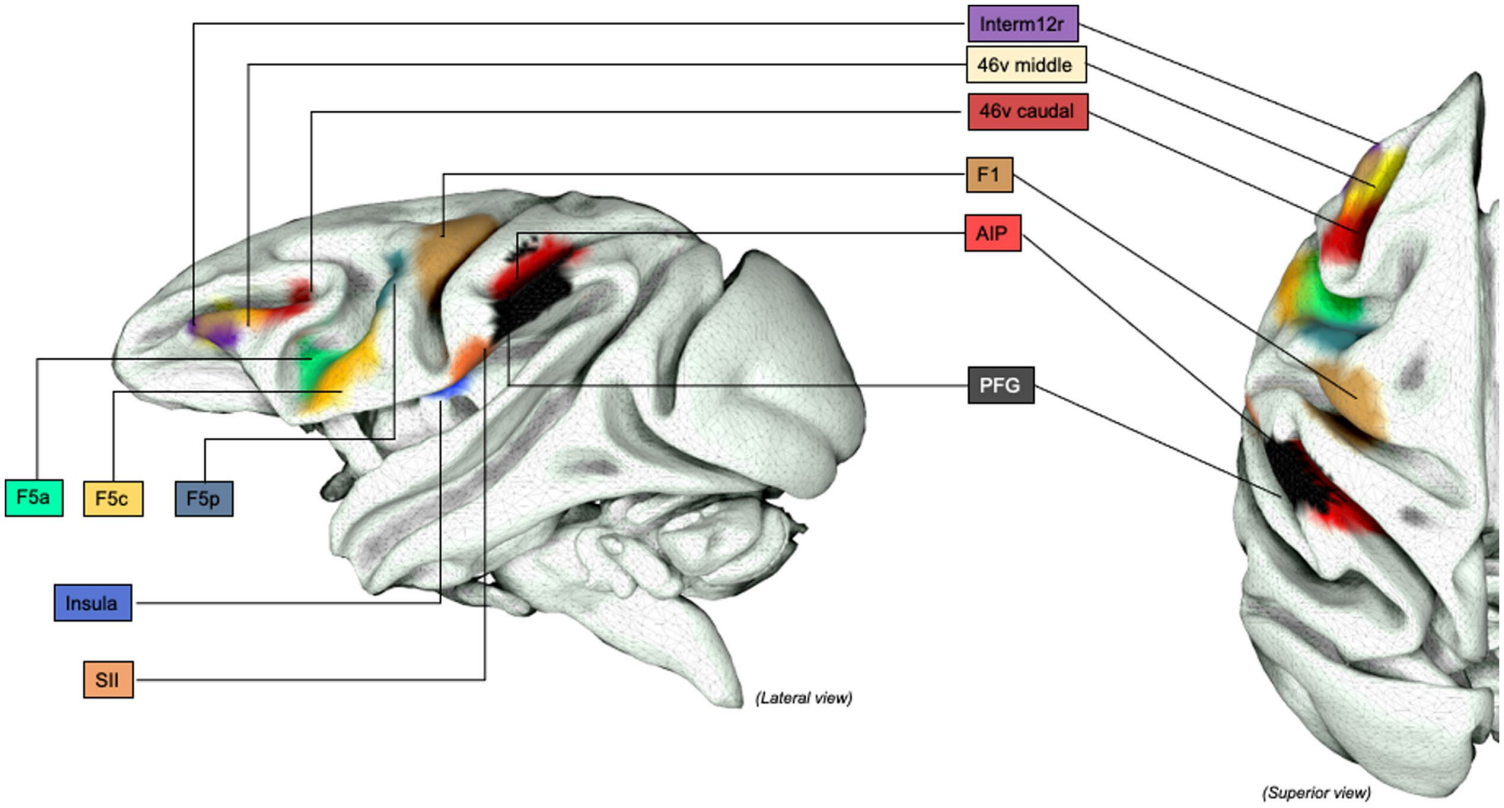
Grasping Network (non-human primates): Graphical representation of the lateral grasping network in non-human primates (macaques). INIA19 template. Neuromaps Atlas (*Macaca mulatta*). 46, lateral prefrontal cortex, area 46; AIP, Anterior IntraParietal area; F1, frontal motor area F1, macaque homolog of human M1 area; F5, frontal motor area F5, macaque homolog of human vPM area; anterior (F5a), posterior (F5p) and convexity (F5c); PFG, posterior parietal area PFG; SII, Secondary Somatosensory cortex; Insula, insular cortex (anterior); Interm12r, intermediate segment of rostral frontomesial area 12.

**Figure 4:**
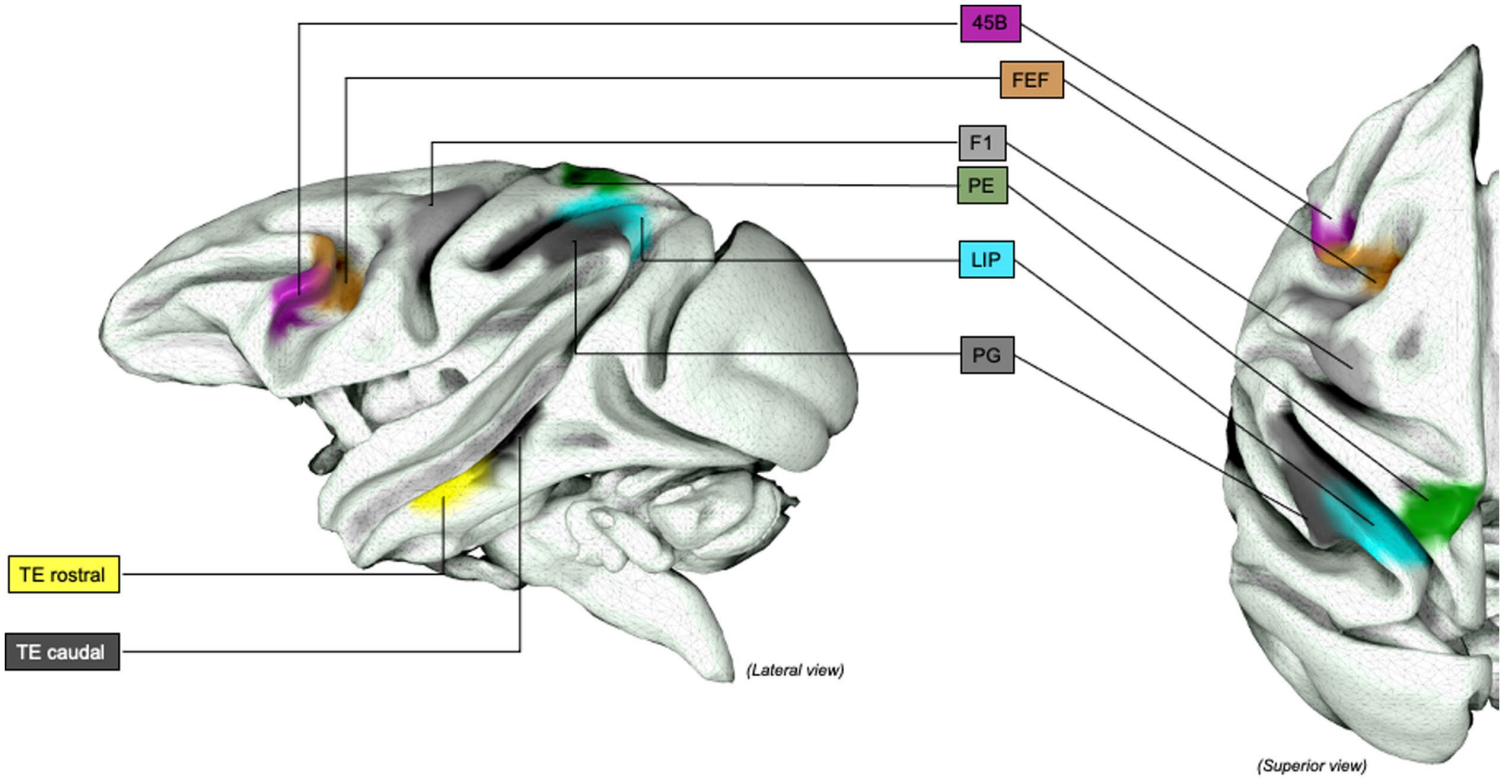
Grasping Network (non-human primates), extended representation: Graphical representation of the extended lateral grasping network in non-human primates (macaques). INIA19 template. Neuromaps Atlas (*Macaca mulatta*). 45B, part of the macaque homolog of Broca’s area; AIP, Anterior INtraParietal area; F1, frontal motor area F1, macaque homolog of human M1 area; FEF, Frontal eye Field; LIP, lateral IntraParietal Sulcus; PE, posterior parietal are PE; PG, posterior parietal area PG; TE, inferior temporal area TE, rostral and caudal.

The behavioral contrasts reported by the authors are the following: Grasp > Rest (Hecht et al., 2013); Reach-and-Grasp > Rest (Nishimura et al., 2007; Hopkins et al., 2010; Nelissen and Vanduffel, 2011; Fiave et al., 2018; Nelissen et al., 2018); Reach-and-Grasp > Reach (Nelissen and Vanduffel, 2011; Nelissen et al., 2018); Grasp > transitive and intransitive passive observation (Hecht et al., 2013); Reach-and-Touch > Rest (Fiave et al., 2018) and Grasp > Touch (Fiave et al., 2018). Additional information on the study design and findings are reported in Table 1. The results of the individual studies will be summarized in the narrative discussion and in Figures 3, 4.

**Table 1:** Main findings (non-human primates): Abbreviations are reported in the main table.

References	Imaging technique	Sample (*N*)	Age	Handedness	Target	Contrast (i.e. Grasp > Rest)	Category	Details	Cortical areas involved	Principle findings	Abbreviations
Nishimura et al. (2007)	FDG-PET; 3 T MRI	3 macaques (2 *Macaca mulatta* and 1 *Macaca fuscata*)	N/A	N/A	Whole brain	Reach-and-Grasp > Passive feeding (Rest)	Grasping (visually-aided)	The monkeys were seated on a monkey chair and trained to reach from a fixed starting position, grasp and retrieve a small piece of sweet potato or carrot (about 7 mm cubic) through a narrow vertical slit using both index finger and thumb with a constant pace. The monkeys performed a series of reach–grip–retrieve–eat movements once every 5 s. In the control task, the monkeys were given the food piece stuck on the tip of the rod into their mouth through a long tube.	Contralateral PMd, M1, S1, pulvinar, VIP, MIP (minor activation in LIP), AIP, PO (V6 and V6a). Ipsilateral intermediate and lateral deep cerebellar nuclei, intermediate zone of cerebellar cortex and medial bank of calcarine sulcus	Reach-and-Grasp > Rest: Activation was consistently observed in the parietal regions such as PO, MIP, VIP, LIP and AIP, frontal regions such as PMd, M1 and S1 on the contralateral hemisphere and in the ipsilateral intermediate and lateral deep cerebellar nuclei.	Area AIP, anterior portion of the lower bank of the intraparietal sulcus; Area PFG, anterior portion of the inferior parietal lobule; S1/S2, primary/secondary somatosensory region; F1, hand field of primary motor cortex; F5c/F5p/F5a, ventral premotor areas; F6, pre-supplementary motor area; vlPF, ventrolateral prefrontal cortex.
Hopkins et al. (2010)	FDG-PET; 3 T MRI	PET study: 4 Chimpanzee (3 females); MRI study: 70 Chimpanzee (48 females)	PET study: 14–18 years; MRI study: (Mean = 21.52, s.d. = 11. 59).	Right (1) and Left hand (3)	Whole brain	Reach-and-Grasp > Rest	Grasp (not specified)	PET study: The goal of the current study was to evaluate regional cortical activation using positron emission tomography (PET) in chimpanzees performing a reach-and-grasp task. Thus, the aim of the study was to determine if the KNOB is significantly activated when chimpanzees produce prehensile reaching-and-grasping actions. MRI study: We subsequently constructed a probabilistic map of the KNOB region in chimpanzees in order to assess the overlap in consistency in the anatomical landmarks of the KNOB with the functional maps generated from the PET analysis	Contralateral: Motor hand Knob, dPCG, dMFG, vPM, OL, SMG, Precuneus, SFG, Superior parietal cortex; Ipsilateral: SFG, Superior parietal cortex, OL, vPM, LG	PET study: Significant clusters were found in the region corresponding to the KNOB in the hemisphere contralateral to the hand used for grasping. In addition, significant clusters in the contralateral hemisphere were found for the medial and ventral premotor areas, dorsal primary motor cortex, and the superior frontal gyrus. MRI study: We compared right- and left-handed chimpanzees on lateralization in gray and white matter within the KNOB region and found that asymmetries in white matter of the KNOB region were larger in the hemisphere contralateral to the preferred hand.	
Nelissen and Vanduffel (2011)	fMRI; 3 T	2 Rhesus m. (2 male)	3–5 years	Right hand	Whole brain	Reach-and-Grasp > Rest; Reach-and-Grasp > Reach	Grasp no vision	Functional magnetic resonance imaging (fMRI) of brain activity while macaque monkeys performed reaching and grasping movements in a 3 tesla MR scanner	Grasp > Reach: F5 (F5p, F5a), F4, granular frontal opercular (GrFO) area, F1, SI (area 3a, 3b, 1, and 2), 5 (PEip and PE), AIP, PFG, SII, parietal ventral area (PV), ventral somatosensory area (VS) and parietal rostral area (PR). Grasp > Rest: AIP, F5(F5p and F5a), V6A, MIP (also termed PRR) did not differentiate between the grasping and reaching tasks, showing almost equal increases in MR signal relative to fixation.	Grasp > Reach: Significant signal changes in portions of contralateral premotor F5 (mainly sectors F5p and F5a in the arcuate sulcus) and F4, as well as in a region anteroventral to F5a designated the granular frontal opercular (GrFO) area. Grasping vs. reaching revealed larger activity in motor area F1, SI (area 3a, 3b, 1, and 2), in area 5 (PEip and PE), area AIP and PFG. Additional activations were revealed in area SII, parietal ventral area (PV) and ventral somatosensory area (VS) and parietal rostral area (PR). Regions activated in the ipsilateral hemisphere included premotor F5, the hand region F1, area 3a and 3b and portions of IPL areas PFG and PG; Grasp > Rest: The time courses of key regions involved in grasping control, AIP and the two F5 sectors located in the arcuate sulcus (F5p and F5a). Areas V6A and MIP (also termed PRR) did not differentiate between the grasping and reaching tasks, showing almost equal increases in MR signal relative to fixation.	
Hecht et al. (2013)	FDG-PET; 3 T MRI	4 Chimpanzee (2 females);	N/A	Right hand	Whole brain	Grasp > Rest; Grasp > Transitive Observation; Grasp > Intransitive Observation	Grasp no vision	Chimpanzees underwent three functional neuroimaging conditions (Figure 1): (1) performance of a manual, transitive (object-directed) grasping action; (2) observation of a human experimenter demonstrating the same action; and (3) observation of an intransitive version of this action in which the demonstrated grasping movement was mimed without touching any object. Chimpanzees performed grasping actions in the execution condition with the right hand; these actions were performed inside a metal box so that subjects were unable to view their own movements. Chimpanzee subjects drank a 15 mCi dose of FDG mixed in sugar-free Kool-Aid, performed the behavioral task for each condition, and then were anesthetized and scanned. PET images were coregistered to and masked with skull-stripped MRI images so that only voxels relating to the brain would be analyzed. The number of activated voxels in predefined ROI, in each condition, in each subject was calculated.	LOC: OA (BA 19); IT: TE1 (BA 21), TE2 (BA 20), PH (BA 37); STS; SPL: PEm (BA 5), PEp (BA 5); IPL: PFD, PF (BA 40/7b), PG (BA 39/7a); S1-S2: PB (BA 3, BA 1), PC (BA 2); M1: FA (BA 4); PMd: FB (BA 6), FC (BA 8); PMv: FBA (BA 6); DLPFC: FDm (BA 9), FD (BA 46); VLPFC: FCBm (BA 44), FDp (BA 45).	The Grasp > Rest contrast revealed left-lateralized clusters of activation in primary motor cortex (in the vicinity of the hand and arm representations), ventral premotor cortex, inferior frontal gyrus, inferior parietal cortex, and lateral temporal cortex. The contrasts for Grasp > Transitive Observation and Grasp > Intransitive Observation produced clusters in inferior parietal cortex. The anterior aspect of this cluster is most likely in area AIP. Small clusters also occurred around the border of the precentral gyrus (area FBA, homologous to BA 6) and pars opercularis of the inferior frontal gyrus (area FCBm, homologous to BA 44).	
Fiave et al. (2018)	fMRI (MVPA method); 3 T	2 Rhesus m.	3–5 years	Right hand	Left hemisphere	Reach-and-grasp > Rest; Reach-and-touch > Rest; Grasp > Touch	Grasp no vision	The subjects were trained to perform two different manual motor acts within MRI gantry: a reach-and-grasp movement or a reach-and-touch movement. (Grasp) After the monkey had grasped the object, he was required to lift it 5mmand hold it in that position for at least 530 ms (maximum holding time 2,000 ms). (Touch) The monkey was required to reach forward and place his open hand on the object. Contrast agent, monocrystalline iron oxide nanoparticle (MION), was injected into the femoral/saphenous vein (6–11 mg/kg). The contrast agent improved the contrast-noise ratio. Each ROI was manually selected on the basis that it had previously been shown to either (a) house mirror neurons, or (b) to be involved in action execution and/or action observation.	F1, F2, vlPF, SII, F5p, F5c, F5a, F6, SI, SII, AIP, PFG	In general, execution of both types of motor acts (compared to fixation only baseline), yielded strongest responses in anterior parietal, motor, somatosensory and frontal cortices. Executed grasps vs. touches yielded significantly distinct multi-voxel patterns in contralateral.	
										Cortex and could be decoded accurately in both monkey subjects in parietal areas AIP and PFG, ventral premotor areas F5c and F5a, primary motor (F1) cortex and dorsal premotor cortex F2.	
										Ventrolateral prefrontal cortex (vlPF), secondary (SII) somatosensory, ventral premotor F5p, dorsal premotor F6 and SI ROIs yielded significant decoding for both motor acts.	
Nelissen et al. (2018)	fMRI (Univariate + MPVA method); 3 T	2 Rhesus m (2 male).	3–5 years	Right hand	Whole brain	Reach-and-Grasp > Rest; Reach-and-Grasp > Reach	Grasp no vision	Functional magnetic resonance imaging (fMRI) of brain activity while macaque monkeys performed reaching and grasping movements in a 3 tesla MR scanner. In the main fMRI experiment, monkeys were trained to grasp 3 different objects: a small cube (sides 12 mm length) or 2 spheres of 23 or 30 mm radius. More specifically, by undertaking a multiclass decoding analysis, we investigated whether different hand configurations during grasping of the 3 differently sized objects (Figure 6A, black circle) could be decoded from the MR signals obtained from the parietal and frontal regions of the lateral grasping or medial reaching circuits.	Reach and grasp > Rest: Contralateral AIP, PFG and F1 (Hand M1). Ipsilateral: F5, S2. Reach and grasp > Reach only: AIP, F5. Hand configurations decoding: V6A, F2, MIP (only in monkey 1). Cube > sphere (30 mm) decoding: PFG, V6A, and MIP Cube > sphere (23 mm) decoding: V6A (only in monkey 1)	The analysis revealed significantly stronger responses for reach-and-grasp than for reach-only in ventral premotor area F5 and anterior parietal area AIP, while MR signal increases during reach-and-grasp and reach-only tasks in posterior parietal area V6A, and dorsal premotor area F2 were not significantly different. At the MVPA analysis, in both animals, hand configurations could also be decoded significantly from parietal area V6A and dorsal premotor F2. Area MIP allowed significant decoding for all objects only in monkey M1. Significant decoding for each of the 3 pairwise target objects comparisons was observed in parietal area AIP, premotor areas F5 and F2, and primary motor area F1, in both animals. ROIs from areas PFG, V6A, and MIP yielded significant decoding specifically for cube vs. the biggest sphere (30 mm) in both animals. In addition, cube vs. smallest sphere (23 mm) could also be decoded above chance from area V6A in monkey.	

#### 3.1.2 Healthy human candidates

Seventy-nine studies were completed between 1996 and 2022, including 1,412 healthy patients (560 females, 9 studies did not specify gender heterogeneity; Grafton et al., 1996; Matsumura et al., 1996; Binkofski et al., 1999a,b; Ehrsson et al., 2000, 2001, 2007; Kuhtz-Buschbeck et al., 2001; Chapman et al., 2002, 2007, 2011; Culham et al., 2003; Grèzes et al., 2003; Ward and Frackowiak, 2003; Frey et al., 2005, 2015; Talati et al., 2005; Shmuelof and Zohary, 2006; Cavina-Pratesi et al., 2007; Grol et al., 2007; Króliczak et al., 2007; Milner et al., 2007; Vaillancourt et al., 2007; Begliomini et al., 2007a,b, 2008, 2014, 2015; Park et al., 2008; Stark and Zohary, 2008; Verhagen et al., 2008; Gallivan et al., 2009, 2011a; Hinkley et al., 2009; Matsuda et al., 2009; Spraker et al., 2009; Cabinio et al., 2010; Kurniawan et al., 2010; Fiehler et al., 2011; Holmström et al., 2011; Hong and Jang, 2011; Kim et al., 2011, 2021; Martin et al., 2011; Monaco et al., 2011, 2014, 2015, 2017; Neely et al., 2011; Glover et al., 2012; Makuuchi et al., 2012; Nathan et al., 2012a; Vingerhoets et al., 2012a; Renzi et al., 2013; Rossit et al., 2013; Fabbri et al., 2014, 2016; Plata et al., 2014; Gutteling et al., 2015; Pavlova et al., 2015; Hamzei et al., 2016; Leo et al., 2016; Marangon et al., 2016; di Bono et al., 2017; Gatti et al., 2017; Przybylski and Króliczak, 2017; Ariani et al., 2018; Cavina-Pratesi et al., 2018; Styrkowiec et al., 2019; Marneweck and Grafton, 2020; Sulpizio et al., 2020; Turella et al., 2020; Bencivenga et al., 2021; Knights et al., 2021, 2022; Errante et al., 2021a; Livne et al., 2022; Michalowski et al., 2022; Ras et al., 2022).

All 79 studies investigated cortical sites of activation in the left and right hemispheres. Seventy-two studies included right-handed patients, and seven included left-handed participants (Begliomini et al., 2008; Cabinio et al., 2010; Martin et al., 2011; Gallivan et al., 2011a; Vingerhoets et al., 2012b; Fabbri et al., 2014; Gutteling et al., 2015); two studies did not declare the participants’ handedness (Knights et al., 2021, 2022). Seven studies on right-handed patients investigated grasping task of the non-dominant hand (Binkofski et al., 1999b; Ward and Frackowiak, 2003; Shmuelof and Zohary, 2006; Hong and Jang, 2011; Kim et al., 2011; Vingerhoets et al., 2012a; Begliomini et al., 2015).

Twenty-two studies performed experiments with no direct view of the target of the grasping task (“i.e. grasping in the dark; Ehrsson et al., 2000, 2001, 2007; Kuhtz-Buschbeck et al., 2001; Shmuelof and Zohary, 2006; Milner et al., 2007; Spraker et al., 2009; Kurniawan et al., 2010; Fiehler et al., 2011; Holmström et al., 2011; Hong and Jang, 2011; Kim et al., 2011, 2021; Neely et al., 2011; Renzi et al., 2013; Fabbri et al., 2014; Marangon et al., 2016; Gatti et al., 2017; Ariani et al., 2018; Cavina-Pratesi et al., 2018; Styrkowiec et al., 2019; Turella et al., 2020). Moreover, eight studies reported specific hand manipulation tasks with (Binkofski et al., 1999a,b; Pavlova et al., 2015; Michalowski et al., 2022; Ras et al., 2022) and without visual aid (Talati et al., 2005; Marangon et al., 2016; Styrkowiec et al., 2019), and two studies acquired functional imaging during a pointing behavioral task (Frey et al., 2005; Cavina-Pratesi et al., 2018).

**Figure 5:**
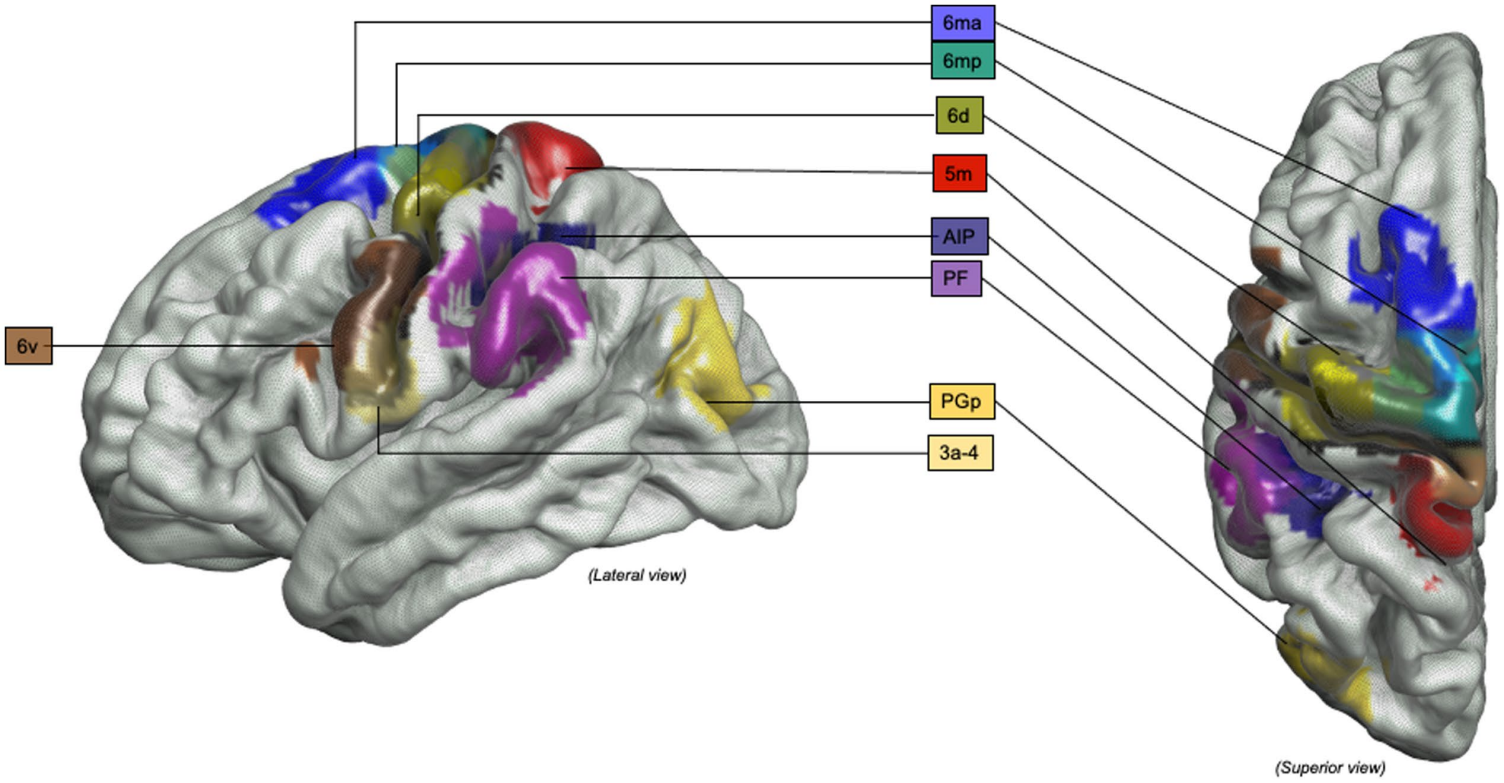
Object-oriented hand manipulation network in humans: Graphical representation of the object-oriented hand manipulation network (within the praxis-representative network) in humans. Atlas, Human Connectome Project-MMP1.0; 3a-4, Brodmann areas 3a-4; 5 m, Brodmann area 5 medial; 6ma, Brodmann area 6ma (preSMA, mesial SFG); 6mp, Brodmann area 6mp (SMA, mesial SFG); 6d, Brodmann area 6d, premotor area dorsal; 6v, Broadmann area 6v, premotor area ventral; AIP, Anterior IntraParietal area; PF, Inferior Parietal Lobule area PF; PGp, posterior portion of human Angular Gyrus.

**Figure 6:**
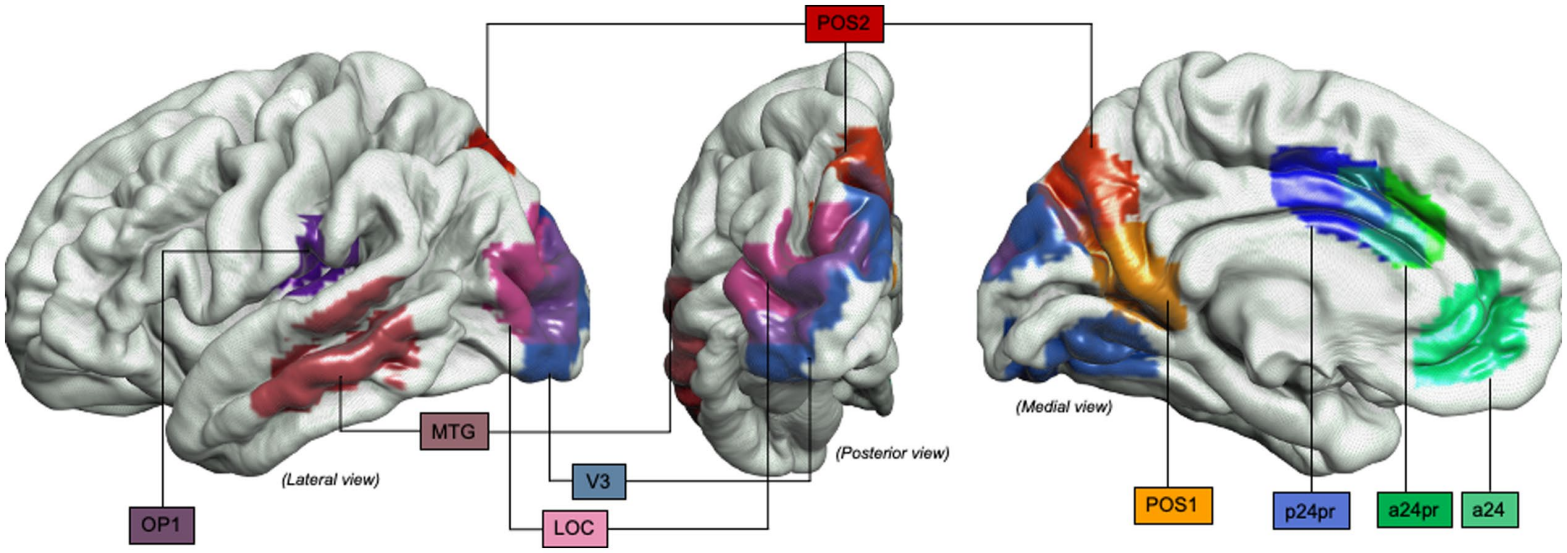
Object-oriented hand manipulation network in humans (extended view): Graphical representation of the extended object-oriented hand manipulation network (within the praxis-representative network) in humans. Atlas, Human Connectome Project-MMP1.0; a24, subdivision of Anterior Cingulate Cortex; a24pr, subdivision of the anterior part of Middle Cingulate Cortex; LOC, Lateral Occipital Cortex; MTG, Middle Temporal Gyrus; OP1, Operculum Parietal 1 area; p24pr, subdivision of the posterior part of Middle Cingulate Cortex; POS, Parieto-Occipital Sulcus; V3, Visual Area 3.

Additional information, including behavioral contrasts implemented, is available in Supplementry Tables 1–3. The results of individual studies will be summarized in the narrative discussion and reported in Figures 5, 6.

### 3.2 Intraoperative awake surgery findings

**Figure 7:**
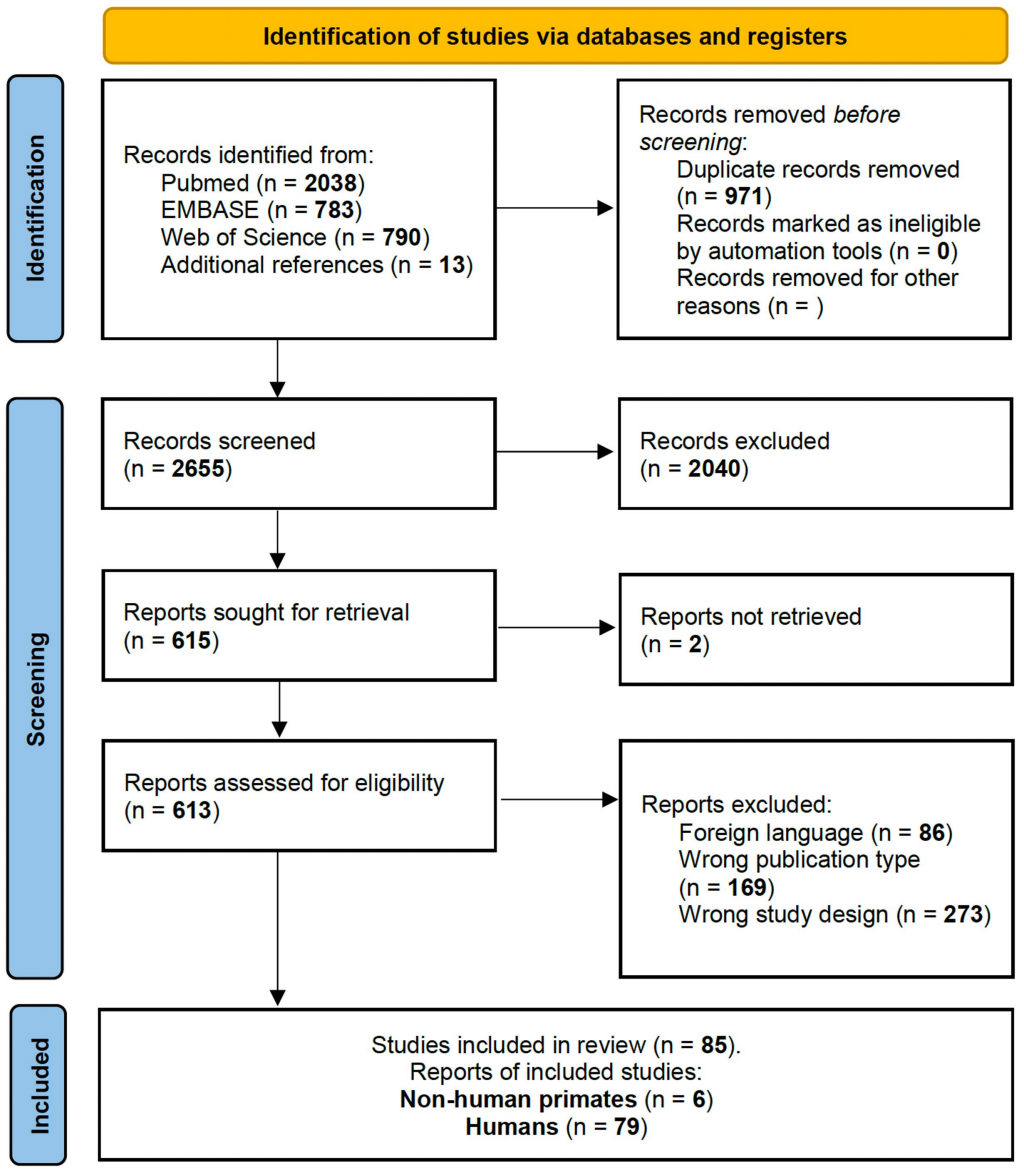
Intraoperative brain mapping stimulation sites distribution during DES-induced hand manipulation task (hMT) disruption: Distribution of stimulation sites in the standardized left hemisphere. The sectors are segregated according to the EMG and behavioral response during DES administration and online execution of the hand-manipulation task (hMT). The “arrest response sites” were defined as those anatomical sectors containing effective sites associated with deficient muscle performance, characterized by complete task arrest during DES. The “clumsy response sites” were defined as those anatomical sectors for which effective sites were associated with high variability among muscles, possibly reflecting poorer muscle coordination. The arrest response pattern is clustered in the ventral and dorsal sectors of the precentral (PreCG, PMd), the postcentral gyrus (postCG) and the anterior sector of the posterior parietal cortex (aPPC). In contrast, the clumsy pattern is identified in the dorsal vPM and the PostCG’s mid-portion. Not represented in the figure: Within the arrest behavioral responses, a “suppression response,” determined as a hand movement arrest and a general decrease in muscle recruitment, was identified in the precentral gyrus in correspondence with a sector within the dorsal vPM and in within arrest sites within aPPC, PostCG and anterior supramarginal gyrus. Similarly, a “recruitment response,” characterized by an involuntary movement and a general unspecific increase of muscles activity within an arrest behavioral phenomenon, was localized in a dorsal sector of PMd and a caudal PostCG sector. Mixed muscle effects were also identified within the caudal PostCG sector. Yellow, arrest response; Green, clumsy response.

For Q2, bibliographic searches on literature databases yielded 4,892 records (PubMed: 1,790; Embase:1,686; Web of Science: 1,415). After removing duplicates (1,916 records) and unrelated manuscripts, 287 records were selected for full-text evaluation. Among these, 269 were further excluded, as they did not meet the predefined inclusion criteria. Overall, 20 records were included in our systematic review. Figure 2 shows the flow diagram of the literature search and study selection. The results of individual studies will be summarized in the narrative discussion (see also Table 2 and Figure 7).

**Table 2:** Main intraoperative findings during awake brain mapping procedures (human primates).

References	Sample (*N*)	Age (years)	Gender	Handedness	Condition	Laterality	Test/Paradigm description	Anesthesia	Category	Localization technique	Details	Preoperative deficit	Morbidity	EOR	Cortical areas involved	Principle findings	Abbreviations
Rosenberg et al. (2010)	26	Mean 39.3 years (21–68)	M:F = 18:8	R:L = 22:4	SMA lesions	LH:RH = 19:4	Uni- and bi-manual finger tapping	Local	Dexterity	LF (bipolar) with previous fMRI	Patients were instructed to perform a sequence of 3 numbers to which they had to tap the correct finger. The evaluation of motor function was documented according to the number of errors in tapping as well as on slowness and hesitations in performance.	17 seizures, 5 motor deficit, 3 speech impairment	6 postoperative new deficits: the single patient who exhibited immediate motor neurological deterioration had motor SMA dominance ipsilateral to the lesion and did not exhibit any deterioration during DCS. The deficit was transient.	N/A	Direct cortical stimulations of the SMA region caused motor dysfunction in 14 of 26 patients. In 12 of the 14 patients, the SMA lesion was on the left side (10 were right handed), and in 2 of the patients the SMA lesion was on the right side (1 was right handed).	We suggest that DCS does not cause functional dysfunction if a compensatory network exists, possible by the recruitment of the nonlesioned SMA or areas in the vicinity of the lesion. When DCS results in some functional deficit, such a compensatory mechanism does not exist or is insufficient to sustain the relevant function.	DCS, Direct Cortical Stimulation; SMA, Supplementary Motor Area.
Schucht et al. (2013)	21	Mean 35 years (24–50)	M:F = 9:12	R:L = 17:4	LGG	LH:RH = 16:5	Continuous alternate flexion/extension of limbs, hand and fingers.	Local	Coordination	LF (bipolar)	Continuous alternating flexion and extension of the arm, hand, and fingers at a frequency at approximately 0.5 Hz.	Seizures were the presenting symptoms in all patients. Four patients had a slight speech deficit and none had a motor or sensory deficit prior to surgery.	All patients recovered well from surgery and were discharged home within 1 week following surgery. 15 patients experienced postoperative worsening of speech. Akinesia was noted in the contralateral arm in 1 patient and in both the contralateral arm and the leg in another 1. All patients with neurological worsening underwent rehabilitation at home; on re-examination at 3 months all had regained their respective preoperative level.	N/A	N/A	The diverse interferences with motor function resulting in inhibition and acceleration imply a modulatory influence of the detected fiber network.	
																The subcortical stimulation sites were distributed veil-like, anterior to the primary motor fibers, suggesting descending pathways originating from premotor areas known for negative motor response characteristics. Further stimulation sites in the parietal white matter as well as in the anterior arm of the internal capsule indicate a large-scale fronto-parietal motor control network.	
Rech et al. (2014)	8	Mean 41.7 years (31–53)	N/A	N/A	frontal LGG	LH:RH = 4:4	continuous alternate flexion/extension of limbs, hand and fingers.	local	coordination	LF (bipolar)	Our protocol of functional monitoring during tumor resection required patients to perform continuous movements of the controlateral upper extremity. This test consisted of repetitive and alternating flexion and extension of the arm, hand and fingers at a frequency at 0.5 Hz. The same continuous movements of the contralateral lower extremity were also required during the resection of the postero-mesial part of the frontal tumor.	Seizures were the presenting symptoms in all patients. None of them had motor deficits or language disorders on neurological examination	All patients recovered well from surgery and were discharged home within 5 days following surgery. 2 patients experienced a slight paresis of the upper limb and ataxia was noted in one case. 1 patient had a mustism and another patient had a slight dysarthria. All patients with neurological worsening underwent rehabilitation at home. On re-examination at 3 months, all patients had regained their respective preoperative level, with no neurological deficit, especially no disorders of bimanual coordination.	N/A	Positive motor responses were found for each patient over the primary motor cortex; the same positive responses were observed during subcortical stimulations of the corticospinal tract. No NMR was elicited with cortical stimulation. Unilateral NMR (UNMR) were elicited at the subcortical level for each patient: sites of stimulations were located at the level of the white matter underneath the premotor cortex, immediately in front of the precentral sulcus, in a veil-like in a coronal plane manner. In addition, bilateral NMR (BNMR) were elicited in all eight patient at the subcortical level, both for inphase and antiphase movement: sites of stimulation were located at the level of the white matter underneath the dorsal premotor cortex and the posterior part of the SMA, rostrally to the corticospinal tract—whatever the side. These BNMR were localized between the sites responsible of UNMR, in the same coronal plane. Subcortical fibers responsible for NMR were followed deeper throughout the resection. Sites of BNMR were found at the level of the anterior arm of the internal capsule and at the level of the head of the caudate nucleus.	We suggest that the BMMP could modulate the excitatory output (“pyramidal” tract) through inhibitory signals coming from each hemisphere at the same time, to synchronize the motor programs of both hands, and thus to allow bimanual coordination. Indeed, the absence of postoperative permanent deficit of bimanual coordination in patients with underwent a large resection within the frontal lobe, is in favor of such a role of the BMMP, since this pathway was in essence preserved during surgery.	NMR, Negative Motor Response.
Rech et al. (2016)	18	mean 31.9 (27–65)	M:F = 9:9	R:L = 15:2 + 1both	frontal LGG	LH:RH = 8:10	continuous alternate flexion/extension of limbs, hand and fingers.	local	coordination	LF (bipolar), 3DTI 3 months after surgery	our protocol of functional monitoring during tumor resection required patients to perform continuous movements of the controlateral upper extremity. This test consisted of repetitive and alternating flexion and extension of the arm, hand and fingers at a frequency at 0.5 Hz. The NPS checked whether the movements (i) were made continuously or whether they stopped, (ii) whether there was a modification of the frequency (e.g., acceleration or slowdown), and (iii), whether there was a modification of the bilateral coordination	None of them had motor deficit (especially no motor initiation disturbance) or language impairment before surgery.	All patients recovered well from surgery and were discharged home within 5 days following surgery. 4 patients experienced a slight paresis of the upper limb, 4 had a worsening of the verbal fluency, and 3 a mutism whose one with a complete akinesia of the hemibody. All patients with neurological disorders underwent rehabilitation at home. On re-examination at 3 months, all patients had regained their respective preoperative level, with no motor neither speech deficits	N/A	During stimulation of the white matter underneath the dorsal premotor cortex and supplementary motor area, rostral to the corticospinal tracts, all patients experienced cessation of the movement of lower and upper limbs, of bimanual coordination, and/or speech. These subcortical sites were somatotopically distributed. Indeed, stimulation of the fibers from mesial to lateral directions and from posterior to anterior directions evoked arrest of movement of the lower limb (mesially and posteriorly), upper limb(s), and face/speech (laterally and anteriorly).	Thanks to the new findings reported in our present study, and knowing that anatomically the FST is more medially located, we can suggest that this tract might be particularly involved in the control of lower and upper limb movements. In addition, we have also previously described that direct stimulation of the frontal aslant tract (FAT, which connects the pre-SMA with the inferior frontal gyrus elicited speech disturbances. Thus, we hypothesize that the motor control network is a complex circuit constituted by multiple tracts, including U-fibers (as suggested above), associative fibers (FAT), and projection fibers (FST), somatotopically organized. Moreover, the bilateral organization of this wide multi-bundle network, as supported by induction of NMRs/BNMRs during stimulation of both hemispheres, might explain plasticity mechanisms underlying functional improvement after a so-called “SMA-syndrome.”	FAT, Frontal Asalnt tract; FST: Fronto-Striatal tract; SMA, Supplementary Motor Area.
Rech et al. (2017)	12 (but 13 surgeries)	mean 40 ± 9 years	M:F = 6:6	R:L = 12:0	frontal gliomas	LH:RH = 5:7	Simultaneous continuous flexion/extension of the contralateral arm, hand, fingers and lower limb, and language task	local	coordination	LF (bipolar)	Our protocol of functional monitoring during tumor resection required patients to perform continuous movements of the controlateral upper extremity. This test consisted of repetitive and alternating flexion and extension of the arm, hand and fingers at a frequency at 0.5 Hz. The NPS checked whether the movements (i) were made continuously or whether they stopped, (ii) whether there was a modification of the frequency (e.g., acceleration or slowdown), and (iii), whether there was a modification of the bilateral coordination	No patient had preoperative neurological deficit, especially for fine movements and bimanual coordination	patients with preservation of the sites eliciting NMR experienced transient motor disorders. 5 patients presented a slight paresis of the upper limb combined with a facial paresis for 2 of them, and 2 patients presented a mutism. No complete SMA syndrome was observed in this group. All patients recovered almost completely from these dysfunctions during the first week following surgery. At 3 months, all patients presented a complete recovery of the motor function and speech. All patients with a resection up to the pyramidal tract presented a complete SMA syndrome associating akinesia of the contralateral hemibody and mutism, if the resection was performed in the dominant hemisphere: recovery began during the first week, initially with lower limb and then for rough movements of the upper limb; mutism disappeared during the same time. At 3 months, they recovered totally from akinesia and mutism but they all presented a dysfunction during fine movements (especially fine fingers movements, like writing) or during action requiring bimanual coordination; moreover, they were unable to perform any synchronous or independent movements of the upper limbs, even 6 months later.	1) 86%, then 62% (100% of CE) 2) 97% 3) 100% 4) 87% 5) 78% (100% of CE) 6) 88% (100% of CE) 7) 85% (100% of CE) 8) 100% 9) 100% 10)100% of CE 11)100% 12)100%	Positive motor responses were elicited for each patient over the primary motor cortex with a somatotopic distribution. Stimulations elicited speech arrest in the white matter under the posterior part of the inferior frontal gyrus, corresponding to face/speech NMR. Slightly more posterior, medial and dorsal, always in the white matter, at the level of the hand knob, stimulations elicited a complete inhibition of the arm, hand and fingers, corresponding to upper limb NMR. More medially, posteriorly and dorsally, lower limb NMR was elicited by inhibiting the movement of the contralateral lower limb. A bimanual NMR was found between sites eliciting upper limb NMR. This distribution was also identified in patient with resection of the NMN.	The preservation of high motors skills requires the monitoring of the negative motor network during surgery by an active motor mapping under awake conditions, whatever the side or handedness.	NMN: Negative Motor Network; NMR: Negative Motor Response;
Rossi et al. (2018)	79	N/A	N/A	R:L = 75:4	27 parietal and 52 frontal gliomas	LH:RH = 54:25	Hand Manipulation Task	asleep-awake-asleep	praxis, precision grip	LF (bipolar)	A specific tool was used for the purpose. It consists of a small cylindrical handle (∅2 and length 6 cm) inserted inside a fixed rectangular base (3 × 3 cm and 9 cm of length) by means of a wormscrew. The rectangular base was kept stable close to the patient’s hand along the armrest of the operating table, while the patient sequentially grasped, held, rotated, and released the cylindrical handle continuously with the thumb and the index finger, using a precision grip. The proximity between the hand and the cylindrical handle allowed the patients to perform the movement using just the fingers, avoiding any reaching movement. Each patient was opportunely trained the day before surgery to perform the HMt at and to report any perceived task-related difficulties, including somatic sensation possibly evoked by LF-DES. The task was performed with the highest regularity paced by an internally generated rhythm without any external cue or visual information about the hand or the cylindrical handle movement. During the procedure, a trained neuropsychologist performed real-time monitoring of the patients’ HMt behavioral outcome, reporting any impairment in task performance and/or any somatic sensation reported by patients. In order to achieve the main aim of the study, an offline analysis of the EMG data recorded during HMt execution was performed. At the beginning of the HMt session, the patient was asked to start the performance at his/her own rhythm to achieve a rhythmic, regular and stable task execution, assessed by online inspection of the behavioral outcome and of the ongoing EMG activity. Once this condition was achieved, LF-DES stimulation of the cortical areas of interest was delivered, randomly during HMt execution, by the surgeon. Stimulations were spaced by 3–4 s to avoid dragging effects.	patients with sensory/motor deficits were excluded	For tumors located in the dominant hemisphere, the incidence of ideomotor apraxia was higher in group B, both at 5 days and at 1–3 months after surgery. Conversely, the incidence of constructional deficits showed no significant difference between groups at 5 days and 1–3 months For tumors located in the nondominant hemisphere, the lower incidence of ideomotor apraxia in group A with respect to group B emerged in the long-term rather than in the acute postoperative period The incidence of constructional deficits, which was statistically different between groups at 5 days, was eventually superimposable in the long term.	mean 96.37%	DH functional boundaries (responses): Frontal Lobe -cortical: M1 (motor responses), vlPM (speech & HMt); -subcortical: M1 (motor responses), vlPM (speech & HMt), SMA/dPM (speech & HMt) Parietal Lobe: -cortical: S1-dorsal (HMt), SMG (HMt, language), AG (language) -subcortical: S1-dorsal (HMt), SMG (language, HMt). NDH functional boundaries (responses): Frontal lobe: -cortical: M1 (motor responses), vlPM (HMt) -subcortical:M1 (motor responses), SMA/dPM (HMt) Parietal Lobe: -cortical:S1-dorsal (HMt), SMG (HMt) -subcortical: S1-dorsal (HMt), SMG (HMt).	HMt is an easily performed tool that allows the identification of specific patterns of interference on task execution (both behavioral and EMG) associated with the different parietofrontal eloquent sites, providing surgeons with an invaluable tool, similar to a fingerprint, to navigate within the praxis network during resection: the M1 block is characterized by tonic muscle activation, the S1 by clonic twitches and release of the object, and the SMG and vlPM by the arrest of movement without muscle activation. Moreover, the stimulation of the dorsomedial sectors of the premotor cortex induced a slow deceleration of the movement and a loss of rhythmicity in the hand-object interaction. Similar features were observed when DES was applied to subcortical sites below the areas described.	HMt: Hand Manipulation task; M1: Primary Motor cortex; dPM: dorsal PreMotor cortex; M1: Primary Motor cortex; S1: Primary Somatosensory Area; SMA: Supplementary Motor Area; SMG: SupraMarginal Gyrus; vlPM: ventro-lateral PreMotor cortex;
	41	N/A	N/A	R:L = 38:3	18 parietal and 23 frontal gliomas	LH:RH = 17:24	PMR	Asleep-awake (for nonmotor functions)-asleep	N/A					mean 93.3%	DH functional boundaries (responses): Frontal Lobe -cortical: M1 (motor responses), vlPM (speech); -subcortical: M1 (motor responses), vlPM (speech), SMA/dPM (speech). Parietal Lobe: -cortical: SMG (language), AG (language); -subcortical: SMG (language). NDH functional boundaries (responses): Frontal lobe: -cortical: M1 (motor responses); -subcortical:M1 (motor responses). Parietal Lobe: -cortical: M1 & S1 (motor responses); -subcortical: M1 & S1 (motor responses).		
Rolland et al. (2018)	14	mean 44 years (17–67)	M:F = 9:5	R:L = 13:1	right IPL gliomas (mostly LGG)	LH:RH = 0:14	N/A	asleep-awake-asleep	N/A	LF (bipolar)	Simultaneously with the naming test, the patient was asked to perform simple repetitive movements of the contralateral left upper limb in a constant manner (flexion of the arm, wrist, and fingers, then extension of the arm while opening the hand and fingers, and so forth every 4 s). We recorded the onset of any modification of the movement (slowness, arrest, lack of accuracy) or the occurrence of involuntary or dystonic movement. Moreover, the patient was asked to inform us immediately when they perceived any abnormal sensation (e.g., hypoesthesia or paresthesia), and to describe it.	13 patients presented with seizures as first symptoms, whereas the discovery was incidental in 1 patient	In the immediate postoperative period, the following deficits were observed: spatial neglect (2 patients), somatosensory disturbances (1 patient), left hemianopia (1 patient), left superior quadrantanopia (3 patients), and mild difficulties with complex movements of the left hand (1 patient). Despite this transitory postoperative worsening, no patient experienced a persistent and severe deficit. All patients recovered within 3 months after surgery, except in 4 patients with left superior quadrantanopia, with no consequences for quality of life.	Total or subtotal resection (i.e., <10 mL of residual tumor) was achieved in all patients but 1.	Identification of cortical somatosensory areas was possible in the 14 patients affected in the postcentral gyrus. Hand or finger dysesthesias (n = 9) were encountered equally as often as those in the forearm, face, tongue, and lips (n = 9). No other eloquent cortical sites were detected by electric stimulation medially and posteriorly. A site involved in naming has been identified by at the IPL/pSTG junction. Several critical sites for spatial cognition were also identified in the posterior supramarginal gyrus (n = 2), at the TPJ (n = 2) as well as in pMTG (n = 1). At the subcortical level the most frequent symptoms while stimulating the thalamocortical fibers were dysesthesia of the face and the left upper limb (n = 12), occasionally in the lower limb (n = 5) and in the abdomen (n = 1). Motor tracts stimulation elicited facial movement (n = 3) or arrest of the movement of the left upper limb (n = 7); articulatory disturbances were elicited by stimulation of the lateral SLFIII (n = 6). Deeper and superiorly, stimulation of the SLFII induced spatial disorders during the line bisection task (n = 5) and vertigo (n = 1). In the lateral and posterior part of the surgical cavity, nonverbal semantic disorders were induced (n = 7) stimulating the right IFOF. Visual deficits (n = 6) were also generated by stimulating the deep and posterior part of the surgical cavity, corresponding to the optic radiations.	right IPL shows a poorly known functional connectivity comprising inferior parietal and posterior temporal lobes, but also associative bundles like SLF system and IFOF: these findings supports awake surgery with not only cortical mapping but also subcortical mapping of the white matter tracts, because they mediate many neural functions to be preserved,	IFOF: Inferior Fronto-Occipital Fasciculus; IPL: Inferiorio Parietal Lobule; pMTG: posterior Middle Temporal Gyrus; pSTG: posterior Superior Temporal Gyrus; SLF: Superior Longitudinal Fasciculus; TPJ: Temporo-Parietal Junction;
Rech et al. (2019)	117	mean 39 years ± 10	M:*F* = 0.95	R:L = 99:14 + 3	LGG	LH:RH = 64:53 (62 controlateral to handedness)	simultaneous continuous flexion/extension of the contralateral arm, hand, fingers and lower limb, and language task	awake	coordination	LF (bipolar)	Simultaneously with the naming test, the patient was asked to perform simple repetitive movements of the contralateral left upper limb in a constant manner (flexion of the arm, wrist, and fingers, then extension of the arm while opening the hand and fingers, and so and forth every 4 s). We recorded the onset of any modification of the movement (slowness, arrest, lack of accuracy) or the occurrence of involuntary or dystonic movement.	no general and motor impairment	No permanent impairments were observed at 3 months after the surgery	N/A	Facial PMRs were located in and around the primary motor area of the face; Upper limb PMRs were located more dorsally (in and around the hand knob). Both extended outside of M1. On both hemispheres, facial NMRs were distributed in two clusters: -cluster A extended over the precentral gyrus from the SFS to the IFS and overlapped with areas 55b and 6d (Glasser parcellation). The maximum probability of finding a facial NMR in this cluster was situated at the junction between areas 6v and 55b on the LH (15%), and within area 55b on the RH (12%), rostrally to the face primary motor cortex. -cluster B located more ventrally, in the ventral premotor cortex; it corresponded to area 6v. Upper limb NMRs were distributed from the sylvian fissure to the hand knob, rostrally to the primary motor cortex. Two clusters were found on the RH: -cluster A located below the IFS and corresponding to areas 43 and 6v and part of area 55b; -cluster overlapped with areas 6d and 55b and the FEF. Three clusters were found on the left hemisphere: -cluster A located below the IFS and corresponding to all of area 6v and parts of areas 43 and 44; -cluster B located rostrally to upper limbs and hands M1, and overlapped within area 6d; -cluster C located between clusters A and B (rostrally to the face’s M1), and covered all of area 55b and the upper part of area 6v.	Our results suggest that: (i) the cortico-subcortical negative motor network has an inhibitory role *per se*; and (ii) PMRs are not artificially disrupted through intracortical inhibitory connections. Clusters of NMAs are located on the dorsal and the ventral premotor cortex, and that these clusters might be functionally connected to the primary motor cortex and the parietal lobe. Hence, these clusters of NMAs might have a role in the control of arm and hand movements during reaching and grasping and in internally or externally driven movements.	FEF: Frontal Eye Field; IFS: Inferior Frontal Sulcus; M1: Primary Motor cortex; NMA: Negative Motor Area; PMR: Positive Motor Response; SFS: Superior Frontal Sulcus;
Viganò et al. (2019)	17	N/A	N/A	R:L = 15:2	right gliomas	LH:RH = 0:17	Hand Manipulation Task	asleep-awake-asleep	praxis	High Frequency Stimulation at rest (HF-DES-Rest), Low Frequency Stimulation during a voluntary hand manipulation task (HMt, LF-DES-HMt) and neuroimaging data by DTI.	A specific tool was used for the purpose. It consists of a small cylindrical handle (∅2 and length 6 cm) inserted inside a fixed rectangular base (3 × 3 cm and 9 cm of length) by means of a wormscrew. The rectangular base was kept stable close to the patient’s hand along the armrest of the operating table, while the patient sequentially grasped, held, rotated, and released the cylindrical handle continuously with the thumb and the index finger, using a precision grip. The proximity between the hand and the cylindrical handle allowed the patients to perform the movement using just the fingers, avoiding any reaching movement. Each patient was opportunely trained the day before surgery to perform the HMt at and to report any perceived task-related difficulties, including somatic sensation possibly evoked by LF-DES. The task was performed with the highest regularity paced by an internally generated rhythm without any external cue or visual information about the hand or the cylindrical handle movement. During the procedure, a trained neuropsychologist performed real-time monitoring of the patients’ HMt behavioral outcome, reporting any impairment in task performance and/or any somatic sensation reported by patients. In order to achieve the main aim of the study, an offline analysis of the EMG data recorded during HMt execution was performed. At the beginning of the HMt session, the patient was asked to start the performance at his/her own rhythm to achieve a rhythmic, regular and stable task execution, assessed by online inspection of the behavioral outcome and of the ongoing EMG activity. Once this condition was achieved, LF-DES stimulation of the cortical areas of interest was delivered, randomly during HMt execution, by the surgeon. Stimulations were spaced by 3–4 s to avoid dragging effects.	Patients with sensory-motor deficits and/or cognitive deficits affecting the motor and/or language function were not included in the study. Only patients without seizures, or with a short seizure history well-controlled by one AED were included.	N/A	N/A	HF-DES-rest: -In patients undergoing MEPs comparison (n = 5) with stimulation at cMT, stimulation successfully elicited reliable MEPs in the caudal sector in the entire sample of muscles analyzed, while when applied on the rostral sector it systematically failed to evoke reliable MEPs that were clearly distinguishable from EMG background activity. This data suggests a non-homogeneous distribution of excitability in the two subsectors, with the caudal one more excitable than the rostral. -In patients (n = 8) in which cMT stimulation was not applied on the rostral sector, using an over-threshold stimulation protocol, MEP amplitudes evoked stimulating on rostral hand-knob were significantly lower compared with the ones evoked stimulating on caudal hand-knob. LF-DES-hMT: two different patterns of interferences: -Dysfunctional Hand Movement (dHM), (10 sites out of 20 (50%); -Suppression of Hand Movement (sHM), (10 sites out of 20 (50%); sites identified were in the right area 4 (upper limb region) and right caudal dorsolateral area 6, respectively. Overall a significant impairment in HMt execution correlated with DES stimulation, although with different features: In dHM sites, DES impaired the task by inducing an accessory activation of hand and arm muscles, producing a dysfunctional hand-object interaction. In sHM sites, DES impaired the task by inhibiting ongoing activation of the muscles required for the movement.	A non-homogeneous rostro-caudal distribution of cortical excitability exists within the hand-knob. The caudal sector showed significantly higher excitability with respect to the rostral one. This result may also be supported by the pattern of muscles activated by the over-threshold stimulations in the two sectors: the same stimulation protocol induced activation of a higher number of muscles when applied to the caudal sector compared with the rostral.	cMT: cortical Motor threshold; DES: Direct Electrical Stimulation; HMt: Hand Manipulation task; MEP: Motor Evoked Potential;
Fornia et al. (2018)	36	mean 42 years ± 12.5 (25–75)	N/A	R:L = 36:0	Left gliomas	N/A	Hand Manipulation Task	Asleep-awake-asleep	Praxis	High Frequency Stimulation at rest (HF-DES-Rest), Low Frequency Stimulation during a voluntary hand manipulation task (HMt, LF-DES-HMt) and neuroimaging data by DTI.	A specific tool was used for the purpose. It consists of a small cylindrical handle (∅2 and length 6 cm) inserted inside a fixed rectangular base (3 × 3 cm and 9 cm of length) by means of a wormscrew. The rectangular base was kept stable close to the patient’s hand along the armrest of the operating table, while the patient sequentially grasped, held, rotated, and released the cylindrical handle continuously with the thumb and the index finger, using a precision grip. The proximity between the hand and the cylindrical handle allowed the patients to perform the movement using just the fingers, avoiding any reaching movement. Each patient was opportunely trained the day before surgery to perform the HMt at and to report any perceived task-related difficulties, including somatic sensation possibly evoked by LF-DES. The task was performed with the highest regularity paced by an internally generated rhythm without any external cue or visual information about the hand or the cylindrical handle movement. During the procedure, a trained neuropsychologist performed real-time monitoring of the patients’ HMt behavioral outcome, reporting any impairment in task performance and/or any somatic sensation reported by patients. In order to achieve the main aim of the study, an offline analysis of the EMG data recorded during HMt execution was performed. At the beginning of the HMt session, the patient was asked to start the performance at his/her own rhythm to achieve a rhythmic, regular and stable task execution, assessed by online inspection of the behavioral outcome and of the ongoing EMG activity. In order to investigate the patient’s ability to monitor his/her motor performance, the HMt was coupled with a verbal MMt in two versions: -in the online MMt, patients were asked to verbally monitor the task overtly, in real time, by saying OK for each grasp-hold-turn phase executed without any difficulty, and by saying STOP when they experienced difficulties in task execution. -In the delayed MMt, patients were asked to answer immediately after DES in PMC to a specific question: Did you correctly execute the motor task? The patient had to answer YES in the case of correct performance and NO in the opposite case.	Only patients either without or with a short history of seizures, well controlled with only one antiepileptic drug, were included in the analysis	N/A	N/A	effective sites were found within the PreCG mainly clustering in vPM cortex (n = 46) and the ventrocaudal sector of the dPM (n = 29) at the border with the upper limb representation in primary motor cortex (M1). No effective sites were found in the IFG and MFG.	stimulation of vPM induced both aCC arrest and aCC clumsy patterns, both mainly characterized by a suppression of motor unit recruitment required by the task. Stimulation of dPM also induced a significant aCC arrest-pattern, mainly characterized by a general recruitment effect, notably preceded by a brief muscle suppression.	dPM: dorsal PreMotor cortex; IFG: Inferior Frontal Gyrus; MFG: Middle Frontal Gyrus; PreCG: PreCentral Gyrus; vPM: ventral PreMotor cortex;
Fornia et al. (2020a)	12	mean 44.83 years (30–58)	N/A	R:L = 11:1	left LGG	N/A	Hand Manipulation Task, Verbal Motor-Monitoring Task	asleep-awake-asleep	praxis, awareness	Low Frequency Stimulation during a voluntary hand manipulation task (HMt, LF-DES-HMt)	A specific tool was used for the purpose. It consists of a small cylindrical handle (∅2 and length 6 cm) inserted inside a fixed rectangular base (3 × 3 cm and 9 cm of length) by means of a wormscrew. The rectangular base was kept stable close to the patient’s hand along the armrest of the operating table, while the patient sequentially grasped, held, rotated, and released the cylindrical handle continuously with the thumb and the index finger, using a precision grip. The proximity between the hand and the cylindrical handle allowed the patients to perform the movement using just the fingers, avoiding any reaching movement. Each patient was opportunely trained the day before surgery to perform the HMt at and to report any perceived task-related difficulties, including somatic sensation possibly evoked by LF-DES. The task was performed with the highest regularity paced by an internally generated rhythm without any external cue or visual information about the hand or the cylindrical handle movement. During the procedure, a trained neuropsychologist performed real-time monitoring of the patients’ HMt behavioral outcome, reporting any impairment in task performance and/or any somatic sensation reported by patients. In order to achieve the main aim of the study, an offline analysis of the EMG data recorded during HMt execution was performed. At the beginning of the HMt session, the patient was asked to start the performance at his/her own rhythm to achieve a rhythmic, regular and stable task execution, assessed by online inspection of the behavioral outcome and of the ongoing EMG activity. Once this condition was achieved, LF-DES stimulation of the cortical areas of interest was delivered, randomly during HMt execution, by the surgeon. Stimulations were spaced by 3–4 s to avoid dragging effects.	N/A	N/A	N/A	DES applied on both PMC (in eight patients) and S1 (in four patients) produced a clear motor impairment in hMT (i.e., evoked suppression of the activity in all muscles considered) in 27 out of 47 stimulated sites (17 over PMC and 10 over S1). During the online MMt version of the task, four patients were stimulated in PMC and four patients in S1. In 88.9% of PMC trials (eight out of nine trials) affecting the HMt, the patients reported online that they were correctly executing the requested action despite the complete arrest of their right-hand movement. Conversely, DES delivered over S1 interrupted motor task execution without altering the patients’ motor awareness. The effect obtained on PMC during the on-line MMt was replicated in an additional four patients tested with the delayed MMt. All patients reported correct execution of the HMt in the 100% of the trials (four out of four trials), despite complete movement arrest due to DES	our results indicate that, during voluntary hand movements, DES on both PMC and S1 interrupted movement execution, while only DES applied on PMC dramatically altered the patients’ motor awareness, making them unconscious of the motor arrest. Taken together, these findings promote the role of PMC as a shared neural substrate for both motor execution and motor awareness of voluntary actions, disclosing a crucial hub in the anatomy-functional network of human motor awareness	DES: Direct Electrical Stimulation; HMt: Hand Manipulation task; MMt: Motor Monitoring task; PMC: PreMotor cortex; S1: Primary Somatosensory cortex;
Monticelli et al. (2020)	21	mean 52.8 years (18–80)	N/A	N/A	gliomas	LH:RH = 12:9	double tasks with both contralateral arm movement and counting	awake-awake-awake	coordination	LF (bipolar)	In order to identify NMRs around the IFG and sensorimotor area, double tasks were required with both contralateral arm movement and counting. When a total motor arrest (TMA) was obtained, the subsequent mapping was performed with a stimulus with 0.5 mA augmented amplitude. TMA was considered as a complete motor and verbal block, without alteration of vigilance or loss of muscle tone. When necessary, “denomination orale d’imagerie” test (DO80), pyramid and palm trees test (PPTT), and reading the mind in the eyes test (RME) were administered in order to identify language functionality and mentalizing responses.	None of the patients had preoperative motor deficits; only one had motor apraxia. None of the patients had preoperative language deficits.	After the surgical procedure, four patients (33.3%) had transient postoperative hyposthenia of the contralateral (3 left, 1 right) superior limb; in all cases, strength completely recovered 1 month after surgery. At 3 months follow-up, 2 of 6 patients, who underwent NMA excision for oncological reasons (2 of the NMAs resected were in the right pRG; 1 in the left pRG, 2 in the right SMG, 1 in the left SMG), had bimanual coordination and fine finger movement deficits. No focal deficit was found at clinical follow-up when the NMAs were preserved (15 patients, 71.4%).	median extent of resection (EOR) of TTV was 82.42% (range 12.60–100%)	A total of 22 cortical TMA was obtained; specifically 1 TMA was recorded during stimulation of the pars opercularis (OpG), 8 TMAs when stimulating the prerolandic gyrus (preRG), 12 TMAs during stimulation of the sensorimotor gyrus (SMG), and 1 TMA after stimulation of the SMA. The tumor was located in the right hemisphere in 9 patients and in these patients we obtained 10 cortical TMAs; in detail: 4 TMAs were registered in the preRG (40%), 5 in the SMG (50%), and 1 TMA was registered while stimulating the SMA (10%). Regarding the preRG, as written, 4 TMAs were registered (40%): 2 were motor arrests, 1 was associated with dysarthria and oral contractions, 1 with contralateral upper limb contraction. In the SMG 5 TMAs were obtained (50%): 1 associated with dysarthria, 1 with speech arrest, 3 without others responses. 7 responses were observed during subcortical stimulation in the right hemisphere: 4 contralateral arm contractions after stimulation of the right corona radiata (CR), 1 TMA after stimulation of the FST, 1 paresthesia on the TCF, and 1 alteration at the RME test, when stimulating the right SLF. The left hemisphere was involved in 12 cortical TMAs: 4 TMAs (33.3%) were recorded in the preRG, 1 TMA in the OpG (8.3%), and 7 TMAs (58.3%) were recorded when the SMG was stimulated. In 2 cases the threshold was found in the preRG (16.6%), in 5 cases it was found in the SMG (41.6%), and in other 5 cases in the OpG (41.6%). In detail, speaking about preRG, as mentioned, 4 TMAs were elicited: 1 of which paired with dysarthria and 1 with oro-buccal apraxia. At the OpG level we found 1 TMA and 4 speech arrests; in 6 cases no responses were found applying DES on the left OpG. When DES was applied on the left SMG, 7 TMAs were elicited; 1 case of phonemic paraphasia and 1 case of dysarthria were registered at the same level; no alteration was apparent in 2 cases. It should be noted that stimulating the left AG, 3 patients manifested anomia (25%) alone, while in 1 case the anomia was associated with phonemic paraphasia; similarly, 4 patients (33.3%) showed paraphasia without anomia caused by left STG and left AG stimulation. At a subcortical level 9 responses were obtained. In 2 cases, contractions were elicited by stimulating the left CR; alteration at the PPTT was observed in 1 case stimulating the left SLF and in other 2 cases stimulating the IFOF. Anomia was induced in 2 cases by left ILF and MdLF stimulation: furthermore, stimulation at the level of the left IFOF induced paraphasia in 1 case.	presence of a wide NMA involving the medium and inferior third of the preRG that seems to have a precise somatotopic organization mimicking Penfield’s homunculus. The finding of a TMA after the stimulation of the SMG and the OpG is in line with what is described in the literature. Furthermore, we registered in 36.4% of cases an extension of the negative cortical motor network to the middle third of the preRG: this location is undoubtably more cranial compared to the one classically described, adjacent to the areas associated with the primary motor cortex related to the face and upper limb. In 9.5% of cases, upon stimulation of the preRG we found movement interruption limited to a body segment according to a somatotopic distribution. There is an association between the resection of NMAs (for oncological reasons) and clinical outcome: In detail, we observed bimanual coordination and fine finger movements deficits at 3 month follow-up in 2 of 6 patients who underwent to NMA resection; on the other hand, no focal deficit was found at clinical follow-up when the NMA was preserved.	AG: Angular Gyrus; DES: Direct Electrical Stimulation; FST: Fronto-Striatal tract; OpG: Inferior Frontal Gyrus pars opercularis; preRG: PreRolandic Gyrus; SLF: Superior Longitudinal Fascicle; SMA: Supplementary Motor area; SMG: Sensory-Motor Gyrus; TCF: thalamocortical fascicle; TMA: Total Motor Arrest;
Cattaneo et al. (2020)	17	Mean 62.59 years (39–79)	M:F = 10:7	R:L = 17:0	Tumors	LH:RH = 9:8	Motor output condition from PPC	Asleep	N/A	HF (monopolar) + dual strip stimulation (and monitoring)	Direct electrical cortical stimulation was applied to the precentral gyrus (test stimuli) via a 6-contacts strip electrode and to the parietal cortex by means of a 6-contacts or an 8-contacts strip electrode. Test stimuli were delivered with trains of the minimal duration required to elicit a stable MEP in the ABP; intensity of test stimulation was set to obtain a MEP from the thenar muscle of around 500 mV peak-peak amplitude. Stimulation of the PPC alone does not produce any measurable output: we used therefore stimulation intensity that was verified to activate corticofugal pathways in the individual patient. To standardize timing precision between the conditioning and the test stimuli between all patients, the conditioning stimuli were always delivered in a short train of 2 stimuli at 250 Hz and of 0.5 ms duration at the same intensity as that of test stimuli. The ISI was considered as the interval between the last stimulus of the conditioning train and the last pulse of the test train based on human data, we would expected interactions to start around 4 ms, hence our choice of ISIs. Every block contained at least 15 repetitions of the same dual stimulation. Cortical and subcortical stimulation were performed using a monopolar probe referenced to Fz (To5, pulse duration 0.5 ms, ISI 2 ms at 1 Hz repetition rate). Cortical stimulation was anodal while subcortical was cathodal.	Motor, seizures, apraxia, ataxia, visual	N/A	N/A	In all participants it was possible to stimulate at least one conditioning spot, with a variability of 3–6. We observed both inhibitory and excitatory effects of conditioning stimuli at different ISIs. Subject variations inherent in the mapping technique were reflected in the variability of the ISIs at which conditioning stimuli exerted a significant effect on corticospinal excitability which ranged from 4 ms to 16 ms. 6 participants showed only inhibitory effects, 3 showed mixed effects, 4 showed faciliatory effects and 4 did not show any effect of conditioning stimuli on corticospinal excitability. Each patient was stimulated with conditioning stimuli in 2–5 pairs of stimulating electrodes. Active spots were localized all along a rostral region of the PPC immediately posterior to the post-central sulcus; in addition, in the few participants in which the conditioning stimulus strip reached the central sulcus, we observed a small cluster of active spots corresponding to the hand motor cortex. The polarity of the effect was spatially organized: -inhibitory effects of conditioning stimuli applied to the SPL and AIP; -excitatory effects from conditioning stimuli applied to the IPL. (most patients showed only facilitatory or inhibitory effects in all stimulation dipoles; in two patients (#1 and #10) we observed a change in polarity of the effect from inhibitory to facilitatory moving the stimulating electrode ventrally and rostrally).	We identified several cortical spots in the posterior parietal cortex that exert a short-latency effect on the excitability of the corticospinal pathway to the upper limb. Combining spatial distribution and polarity (excitatory or inhibitory) of the conditioning effects, we identified 2 distinct regions: a ventral region, corresponding to the part of the supramarginal gyrus immediately posterior to the inferior postcentral sulcus, extending ventrally to the parietal opercular region, where excitatory effects are clustered and a dorsal region comprising in the superior parietal lobule adjacent to the postcentral sulcus, where inhibitory effects cluster. The two clusters are significantly separated in space.	AIP: Anterior IntraParietal area; IPL: Inferior Parietal Lobule; ISI: Inter-Stimuli Interval; PPC. Posterior Parietal cortex; SPL: Superior Parietal Lobule;
Rech et al. (2020)	117 (100)	mean 39 ± 10 years	M:*F* = 48:52	R:L = 82:12 + 3	LGG	LH:RH = 53:47	double task with both contralateral arm movement and naming	awake	coordination?	LF (bipolar)	A language and motor assessment was performed during the corticosubcortical mapping. Patient was performing an object naming task (DO 80) at the same time with a motor task (alternative flexion and extension of the contralateral upper limb at 0.5 Hz frequency). A site was considered functional if the stimulation led to an impairment followed by a normalization of the behavior at the cessation of the stimulation, three times in a non-sequential manner. A DNMR site was defined by a speech arrest and a NMR of the contralateral upper limb at the same time.	N/A	N/A	N/A	On the right hemisphere, DNMR were located over a large surface of the preCG. One site was on the dorsal bank of the post central gyrus and another over the caudal part of the SFG (areas which presented the higher probability were located on the lateral part of the preCG, between the limit of the sylvian fissure and the SFS, the dorsal part of the preCG, above the limit of the SFS, did not harbor any DNMR. On the left hemisphere, DNMR were identified at the same location over the lateral part of the preCG than on the right hemisphere. Again, the most dorsal part of the preCG, just caudally to the SFG, was not involved. Three sites generated DNMR on the postCG, caudally to hand knob whereas two others elicited DNMR on the ventral part of the postCG. One single site generated a DNMR over the pars opercularis.	DNMR seems to be a frequent phenomenon. DNMR over the PMd, in accordance with previous findings, suggest a link between the semantic representation and the motor system. Interestingly, it was also possible to elicit DNMR involving speech and upper limb at the level of the PMv where classically only the face motor system is considered as being linked with language and speech networks. It is interesting to note that the left PMv is widely connected to both preSMA supporting its cognitive role in movement and language beyond speech production or face motricity.	DNMR: Double Negative Motor Response; PMd: PreMotor dorsal; PMv: PreMototr ventral; postCG: postCentral Gyrus; preCG: preCentral Gyrus; SFG: Superior Frontal Gyrus; SFS: Superior Frontal Sulcus; SMA: Supplementary Motor Area;
Rossi et al. (2021)	69	N/A	N/A	N/A	almost exclusively gliomas	N/A	N/A	asleep	PMR	HF (monopolar)		Fewer patients in the awake group had preoperative motor deficits (88% had 5/5 MRC grade deficit vs. 78% in the asleep group), but more patients in the asleep group had poor seizure control than patients in the awake group (44%] vs. 10%) or had received radiotherapy (16% vs. 5%) or undergone previous surgery (51% vs. 24%. Other clinical factors, and particularly the presence of preoperative hand apraxia, were comparable.	Perioperative morbidity was low and comparable between the two groups Immediately after surgery, the rates of apraxia were comparable between the two groups (12% of awake and 19% of asleep, whereas the proportion of patients with severe strength deficit was greater in the asleep group (28% of patients had ≤3/5 MRC grade deficit) than the awake group (14%). SMA syndrome developed in 2 (18%) patients in the asleep group and 2 (14%) patients in the awake group. Most deficits resolved at 1–3 months after surgery, and the proportion of patients with permanent strength deficits (4% of awake group vs. 0% of asleep group) or apraxia (6% of awake group vs. 12% of asleep group) were comparable. However, patients with tumor larger than 30 cm3 involving the praxis network who underwent asleep motor mapping had a higher rate of permanent apraxia (18.75%). Rates of postoperative seizure control were also similar, and most patients were seizure free (Engel class I). A greater proportion of patients in the awake group had abnormalities on immediately postoperative DWI scans than patients in the asleep group (48% vs. 16%): most of these alterations were small (< 1 cm3), were located in both eloquent and noneloquent areas, and resolved on subsequent MR images.	similar between groups (mean EOR 94% for the awake group vs. 96% for the asleep group. However, RTV was larger in patients in the awake group than in patients in the asleep group (mean 3.7 cm3 vs. 1.16 cm3), which is consistent with the fact that preoperative tumor volume was greater in the awake group. Subtotal resection was documented in 36% of patients in the awake group and 18% of patients in the asleep group.		Surgery for tumors located near the eloquent area for motor control is feasible, and when an appropriate mapping strategy is applied, has a low incidence of postoperative motor and praxis deficits. Asleep motor mapping with an HF paradigm is preferable for patients with lesions close to or involving the central sulcus and/or patients with preoperative strength deficit and/or history of previous treatment; when, instead, the patient has no motor deficit or previous treatment and has a lesion extending to or involving the praxis network, awake motor mapping is preferable.	
	66	N/A	N/A	N/A		N/A	HMt	asleep-awake-asleep	praxis	HF + LF	A specific tool was used for the purpose. It consists of a small cylindrical handle (∅2 and length 6 cm) inserted inside a fixed rectangular base (3 × 3 cm and 9 cm of length) by means of a wormscrew. The rectangular base was kept stable close to the patient’s hand along the armrest of the operating table, while the patient sequentially grasped, held, rotated, and released the cylindrical handle continuously with the thumb and the index finger, using a precision grip. The proximity between the hand and the cylindrical handle allowed the patients to perform the movement using just the fingers, avoiding any reaching movement. Each patient was opportunely trained the day before surgery to perform the HMt at and to report any perceived task-related difficulties, including somatic sensation possibly evoked by LF-DES. The task was performed with the highest regularity paced by an internally generated rhythm without any external cue or visual information about the hand or the cylindrical handle movement. During the procedure, a trained neuropsychologist performed real-time monitoring of the patients’ HMt behavioral outcome, reporting any impairment in task performance and/or any somatic sensation reported by patients. In order to achieve the main aim of the study, an offline analysis of the EMG data recorded during HMt execution was performed. At the beginning of the HMt session, the patient was asked to start the performance at his/her own rhythm to achieve a rhythmic, regular and stable task execution, assessed by online inspection of the behavioral outcome and of the ongoing EMG activity. Once this condition was achieved, LF-DES stimulation of the cortical areas of interest was delivered, randomly during HMt execution, by the surgeon. Stimulations were spaced by 3–4 s to avoid dragging effects.				N/A		
Fornia et al. (2023)	34	mean age 46 ± 12.5 years (25–75)	N/A	R:L = 34:0	19 HGG, 14 LGG, 1 other	LH:RH = 34:0	HMt	asleep-awake-asleep	praxis	N/A	A specific tool was used for the purpose. It consists of a small cylindrical handle (∅2 and length 6 cm) inserted inside a fixed rectangular base (3 × 3 cm and 9 cm of length) by means of a wormscrew. The rectangular base was kept stable close to the patient’s hand along the armrest of the operating table, while the patient sequentially grasped, held, rotated, and released the cylindrical handle continuously with the thumb and the index finger, using a precision grip. The proximity between the hand and the cylindrical handle allowed the patients to perform the movement using just the fingers, avoiding any reaching movement. Each patient was opportunely trained the day before surgery to perform the HMt at and to report any perceived task-related difficulties, including somatic sensation possibly evoked by LF-DES. The task was performed with the highest regularity paced by an internally generated rhythm without any external cue or visual information about the hand or the cylindrical handle movement. During the procedure, a trained neuropsychologist performed real-time monitoring of the patients’ HMt behavioral outcome, reporting any impairment in task performance and/or any somatic sensation reported by patients. In order to achieve the main aim of the study, an offline analysis of the EMG data recorded during HMt execution was performed. At the beginning of the HMt session, the patient was asked to start the performance at his/her own rhythm to achieve a rhythmic, regular and stable task execution, assessed by online inspection of the behavioral outcome and of the ongoing EMG activity. Once this condition was achieved, LF-DES stimulation of the cortical areas of interest was delivered, randomly during HMt execution, by the surgeon. Stimulations were spaced by 3–4 s to avoid dragging effects.	Selected patients showed a normal score for the upper limb apraxia (De Renzi test), no basic sensory and motor deficits (neurological examination) and scored 57 (the highest score) in the Action Research Arm test (ARAT)	N/A	N/A	The analysis showed that LF-DES applied in 111 out of 280 stimulated sites in the parietal lobe significantly decreased the aCC of the investigated muscles during HMt execution. These sites were categorized as effective sites, while the sites failing to show significant changes on the aCC value were categorized as ineffective sites. Among the effective sites, two main EMG-interference patterns emerged: -task-arrest patterns (n = 51 sites recorded in 23 patients), where stimulation evoked a complete abolishment of the EMG pattern required by HMt execution, occurring in all muscles and associated to an abrupt arrest of the ongoing task execution; -task-clumsy patterns (n = 60 sites recorded in 23 patients), where stimulation evoked a partial disruption of the EMG pattern required by HMt execution and associated to a clear impairment of finger coordination and/or movement slowdown and loss of contact with the object. The most “eloquent” sectors fell in the PCG fingers representation (BA1/2, primary somatosensory cortex) and within PPC at the junction between intraparietal and postcentral sulcus, involving areas around the anterior intraparietal cortices (aIPC, mainly hIP2 and PFt). 3 different clusters where identified based on EMG activation: -PCG clusters: Cluster 1 hosted prevalently task-arrest patterns falling in the medial hand-finger somatosensory representation, while cluster 2, more lateral, hosted a prevalence of task-clumsy patterns; -PPC cluster: located within aIPC hosted with higher probability task-arrest patterns (although within PPC task-clumsy pattern were not absent, their occurred preferentially within aSMG).	within PCG the medial BA1/2 and aIPC, preferentially associated to task-arrest pattern (PCG cluster 1 and aIPC), might be part of neuronal substrates closely implicated in the shaping of the voluntary motor output to muscles. Differently, the lateral BA1/2, preferentially associated to task clumsy pattern (PCG cluster 2), might act more indirectly respect to the motor output. In this light clumsy pattern might reflect a problem in the sensorimotor integration required by HMt execution. In PCG the LF-DES during task evoked variable levels of muscles recruitment, ranging from the subtle muscle activity during the suppression effects to the more evident muscle recruitment effects associated to an overt involuntary hand movements. Differently, within aIPC, the muscle activity during the suppression effect was comparable to a rest condition. This result suggests that different parietal sectors might synergically shape the mo tor output to hand-muscle by balancing inhibitory and facilitatory inputs.	aIPC: anterior Intra-Parietal cortex; aSMG: anterior SupraMarginal Gyrus; BA: Broadmann area; DES: Direct Electrical Stimulation; HMt: Hand Manipulation task; PCG: PostCentral Gyrus;
Viganò et al. (2022)	34	mean 42 ± 10.6 years (22–64)	M:F = 17:17	R:L = 30:4	gliomas	LH:RH = 15:19	HMt	asleep-awake-asleep	praxis	LF + HF	A specific tool was used for the purpose. It consists of a small cylindrical handle (∅2 and length 6 cm) inserted inside a fixed rectangular base (3 × 3 cm and 9 cm of length) by means of a wormscrew. The rectangular base was kept stable close to the patient’s hand along the armrest of the operating table, while the patient sequentially grasped, held, rotated, and released the cylindrical handle continuously with the thumb and the index finger, using a precision grip. The proximity between the hand and the cylindrical handle allowed the patients to perform the movement using just the fingers, avoiding any reaching movement. Each patient was opportunely trained the day before surgery to perform the HMt at and to report any perceived task-related difficulties, including somatic sensation possibly evoked by LF-DES. The task was performed with the highest regularity paced by an internally generated rhythm without any external cue or visual information about the hand or the cylindrical handle movement. During the procedure, a trained neuropsychologist performed real-time monitoring of the patients’ HMt behavioral outcome, reporting any impairment in task performance and/or any somatic sensation reported by patients. In order to achieve the main aim of the study, an offline analysis of the EMG data recorded during HMt execution was performed. At the beginning of the HMt session, the patient was asked to start the performance at his/her own rhythm to achieve a rhythmic, regular and stable task execution, assessed by online inspection of the behavioral outcome and of the ongoing EMG activity. Once this condition was achieved, LF-DES stimulation of the cortical areas of interest was delivered, randomly during HMt execution, by the surgeon. Stimulations were spaced by 3–4 s to avoid dragging effects.	no preoperative motor deficits, no long-term history of epilepsy	11 patients out of 34 experienced transient postoperative MRC deficits at 5 days from surgery, completely recovered at the 1-month follow up	In 23 patients a supratotal resection and in 11 a total resection was performed (The significant cluster overlapped only with the arrest pattern density map and corresponded with the dorsal white matter region enclosing mainly SMA-projections and the superior fronto-striatal tracts). Only one patient had a pathological score in the De Renzi test in the immediate postoperative phase, which fully recovered at the 1-month follow-up.	Within the stimulated area, the arrest pattern (aCC = 0) was found in 36 sites (54%; 27 in right and nine in left hemisphere), while the clumsy pattern (aCC40) was found in 30 sites (46%, 15 in right and 15 in left hemisphere). In 11 patients, both patterns were observed at different sites. When effective sites were stimulated with high frequency DES (To5) up to 10 mA of intensity, no upper-limb motor evoked potentials were never elicited, suggesting a distance of at least 10 mm to the M1-CST. The two patterns were partially overlapped mainly below the middle frontal gyrus, however they showed a preferential dorso-ventral distribution: (i) the arrest pattern occurred bilaterally in the white matter below the dorsal premotor region; and (ii) the clumsy pattern occurred bilaterally in the white matter below the ventral premotor region, and, only in the left hemisphere, 4 sites out of 15 were reported in the middle anterior cingulum below the pre-SMA. White matter tracts most often recruited included: -association fibers: short U-shaped precentral tracts (mid-U-shaped), the SLF (I, II, and III branches), the FAT, the AF; -projection fibers: the superior and inferior FSTs, M1-CST, dPM-CST, vPM-CST and SMA-CST; and -callosal fibers (although these were not further analyzed). mid-U-shaped were exclusively associated with the arrest pattern, while inferior FST and the SLFIII were uniquely associated with the clumsy pattern. Despite the significant structural segregation, a set of common pathways were associated to both effects, including the superior FST, CST, FAT, AF, SLFI and II.	The existence of two different white matter regions associated to distinct aspect of task-related motor output implementation: the two interference patterns showed that, although they overlapped below the MFG, the arrest pattern occurred preferentially during stimulation of white matter below a dPM region anterior to the precentral hand-knob, whereas the clumsy pattern occurred preferentially within white matter below vPM. The arrest pattern may reflect the disruption of a network closely involved in motor output implementation, while the clumsy pattern may reflect the perturbation of a network possibly involved in sensorimotor computations required for task execution. Short range premotor mid-U-shaped fibers were only associated with the arrest pattern, while iFST and the SLFIII were uniquely associated with the clumsy pattern; moreover the sFST, FAT, AF, SLFI and II were correlated to both effects. The preservation during surgery of dorsal white matter surrounding the SMA is crucial to preserve upper-limb movement integrity in the immediate postoperative phase while in the ventral region no motor deficit was detected. On the other hand, the FAT, AF, SLF and the iFST were commonly resected without any motor disturbances; resection of the latter tracts might rather be associated to higher sensorimotor disorders, such as ideomotor apraxia (although not clearly evinced from present study).	AF: Arcuate Fasciculus; dPM: dorsal PreMotor cortex; FAT: Frontal Aslant tract; iFST: inferior Fronto-Striatal tract; MFG: Middle Frontal Gyrus; SLF: Superior Longitudinal Fasciculus; SMA: Supplementary Motor Area; vPM: ventral PreMotor cortex;
Tomasino et al. (2022)	57	40.52 ± 13.15 years	M:*F* = 22:35	N/A	precentral or postsomatosensory areas tumors	N/A	finger tapping or hand strength tasks	awake	N/A	N/A	For resections in precentral and postsomatosensory areas, we first applied DES to map motor and sensory functions. We then administered the Real Time Neuropsychological Testing: as resection progressed, sequences of test (or RTNT runs) were continuously repeated; neurophysiological monitoring accompanied resection by administering finger tapping or hand strength trials. Runs: i) Handedness decision task (HDT), considered a test monitoring sensorimotor representations as subjects need to imagine reproducing picture position and posture, in order to determine its laterality; ii) Florida Praxis Imagery Questionnaire (FPIQ), testing the general motor imagery ability; iii) Action verb naming (AVN) task; iv) Conceptual knowledge of actions: the Kissing and Dancing Test (KDT), patients were presented with a probe word and had to decide which of 2 verbs corresponded to it. v) Buccofacial praxis and ideomotor praxis (BP and IMP), patients were presented verbally with a gesture description and asked to generate it. vi) STM and WM, monitoring short-term verbal memory (digit span, both forward and backward).	native Italian speakers, having normal or corrected-to-normal vision, and no history of psychiatric disease or drug abuse. We excluded patients with developmental language problems or learning disabilities or with a family history for such disabilities.	N/A	The mean EOR was 91.15% ±17.45	A motor response was detected in 84.21% of the cases for the central area, while in precentral and postsomatosensory areas, cognitive mapping with DES elicited a response in 17.5% of the cases. For the FPIQ task, the maximum lesion overlay included the right precentral and postcentral gyrus, the supplementary motor area, and the superior and inferior parietal lobe. For the HDT, the maximum lesion overlay included the right cingulum/supplementary motor area and left superior and inferior parietal lobe and medial precuneus.	RTNT provides new information on the patients’ action imagery processing. The RTNT approach enabled us to report a decrease in performance of certain cognitive tests, confirming that these areas are involved in action imagery related processing. Considering the 2 tasks showing a higher variability in performance the lesion volume analysis showed an involvement of different areas: -For the FPIQ, the right preCG and postCG, SMA, SPL and IPL; -for the HDT, the right cingulum/ supplementary motor area and left SPL, IPL and medial precuneus.	IPL: Inferior Parietal Lobule; preCG: preCentral Gyrus; postCG: postCentral Gyrus; SMA: Supplementary Motor Area; SPL: Superior Parietal Lobule; RTNT: Real Time Neuropsychological Testing:;
Bennett et al. (2022)	40	mean 40.1 years (18–72)	N/A	N/A	supratentorial gliomas (29LGG, 11HGG)	N/A	N/A	awake craniotomy	N/A	LF (bipolar)	All patients underwent excision surgery with awake craniotomy. Cortical mapping was performed using bipolar stimulation at 60 Hz and a biphasic wave starting at 2 mA, without exceeding 8 mA. Intraoperative mapping findings were documented with photographs. Afterward, 3D fMRI with superimposed cortical vessels was compared with the intraoperative photographs, and confusion matrices were created: considering activation registered during awake surgery as the gold standard, true positive, true-negative, false-positive, and false-negative numbers were summarized for each HCP area.	N/A	N/A	N/A	There was no difference in sensitivity (100% in both locations) and only a 7% difference in specificity (71% in the precentral gyrus and 78% in the postcentral gyrus). Notably, the negative predictive value of motor fMRI was 100% (all cases with negative fMRI in the pre- or postcentral gyrus had negative surgical mapping in these locations), while positive fMRI mapping was not as accurate, and was better in the precentral gyrus (85%) than in the postcentral gyrus (50%). Considering all language areas, sensitivity was worse in specific language fMRI protocols than in motor tasks and was better when activation of any protocol was considered (88%), but with lower specificity (62%). When evaluating specific areas with each protocol, sensitivity was variable (50–100%), as was specificity (20–100%). Notably, areas usually considered eloquent (PSL/STV) showed high specificity with the pseudoword and verb generation protocols and the negative predictive value was 100% in all protocols.	In accordance to existing literature, the concordance of fMRI with DES is generally good for motor mapping, with sensitivities of 71–100% and specificities of 68–100%. High concordance of areas detected using fMRI with specific HCP parcels, such as SFL and 55b (which are not always detected by DES), leads us to rethink the role of cortical mapping in relation to fMRI. While fMRI appears to provide a global vision of the language network in each patient, surgical mapping detects which components of this network behave as hub areas (critical noncompensable nodes), which can be more variable.	DES: Direct Electrical Stimulation; SLF: Superior Longitudinal Fasciculus;
Fornia et al. (2023)	79	49.5 ± 14.8 years (19–76)	M:*F* = 55:24	R:L = 79:0	Supratentorial intra-axial lesions (almost exclusively gliomas)	LH:RH = 79:0	HMt	Asleep-awake-asleep	Praxis	LF + HF	A specific tool was used for the purpose. It consists of a small cylindrical handle (∅2 and length 6 cm) inserted inside a fixed rectangular base (3 × 3 cm and 9 cm of length) by means of a wormscrew. The rectangular base was kept stable close to the patient’s hand along the armrest of the operating table, while the patient sequentially grasped, held, rotated, and released the cylindrical handle continuously with the thumb and the index finger, using a precision grip. The proximity between the hand and the cylindrical handle allowed the patients to perform the movement using just the fingers, avoiding any reaching movement. Each patient was opportunely trained the day before surgery to perform the HMt at and to report any perceived task-related difficulties, including somatic sensation possibly evoked by LF-DES. The task was performed with the highest regularity paced by an internally generated rhythm without any external cue or visual information about the hand or the cylindrical handle movement. During the procedure, a trained neuropsychologist performed real-time monitoring of the patients’ HMt behavioral outcome, reporting any impairment in task performance and/or any somatic sensation reported by patients. In order to achieve the main aim of the study, an offline analysis of the EMG data recorded during HMt execution was performed. At the beginning of the HMt session, the patient was asked to start the performance at his/her own rhythm to achieve a rhythmic, regular and stable task execution, assessed by online inspection of the behavioral outcome and of the ongoing EMG activity. Once this condition was achieved, LF-DES stimulation of the cortical areas of interest was delivered, randomly during HMt execution, by the surgeon. Stimulations were spaced by 3–4 s to avoid dragging effects.	pre-operative absence of pathological score for ideomotor apraxia (De Renzi global score > 53) pre-operative absence of any clinically observable deficit during object prehension-manipulation (ARAT global score = 48)	post-operative MRC upper-limb score ≥ 4. post-operative absence of severe sensory (tactile and visual) deficit. post-operative absence of language comprehension deficits. 19 out of 79 patients reported a lower score in the early post-operative score at the ARAT, post operative 1-month ARAT global score significantly improved compared to the early post-operative phase with no significant difference with the pre-operative phase. 11 out of 79 patients De Renzi global score fell below the cut-off 6 patients were borderline in the early post-operative period; post operative 1-month De Renzi global score significantly improved compared to the early post-operative phase with no significant difference with the pre-operative phase.	N/A	Significant responsive clusters in the PostCG (somatosensory fingers representation), the phAIP47 and, more marginally, the anterior PF/PFt within the IPL Within the PPC, DES effect on HMt ranged from an abrupt arrest (task-arrest) mainly reported within phAIP, to a lack of finger coordination (task-clumsy) mainly reported within anterior IPL (PF), both associated to different degree of muscle suppression. 16 effective sites were localized in the white matter below the fundus of rostral IPS and postcentral sulcus, broadly corresponding to the white matter below phAIP and PF/PFt. Task-arrest (n = 7) responses were mainly found below AIP while task-clumsy (n = 9) were adjacent to the white matter below PF coherently with cortical distribution. 1) Within rostral IPS, the intraoperative manipulation-sites clustered within the anterior part of phAIP, while praxis-related voxels at the transition between phAIP and DIPSA; 2) Within the rostral IPL, despite the lower level of probability, intraoperative manipulation-sites clustered in anterior PF, while praxis-related voxels at the transition between PF and PFm; 3) The matching obtained at cortical level was specular at subcortical level; 4) The anterior IPS was associated to both meaningless and meaningful gestures, while anterior IPL was associated to meaningful gestures. Parallel to this distinction, the manipulation-sites within rostral IPS (phAIP) and IPL (PF) showed different features of motor impairment induced by DES during HMt, task-arrest and clumsy, respectively.	Present results showed a functional dissociation between dorso-dorsal and dorso-ventral streams and within the dorso-ventral one. First, it emerged the existence of a parietal dorso-lateral functional continuum subserving the transition from transitive object-oriented actions (dorso-dorsal pathway) to intransitive praxis gestures (dorso-ventral pathway), with specific rostral IPS sectors possibly working as convergent zone and regulating the flow of information between streams. Moreover, within the dorso-ventral stream our results showed a further dissociation between the role played by rostral IPS (mainly phAIP/DIPSA) and rostral IPL (mainly PF) in the type of gesture to be imitated (meaningless vs. meaningful), to same extent mirroring the anatomo-functional distinction between object-manipulation and object (tool)-use. Notably, the DES applied to these parietal regions evoked different type of motor impairments during the HMt execution, furthermore suggesting that these sectors may subserve distinct pathways for gesture imitation (direct vs. indirect) via different hand-related somatomotor process.	AIP, Anterior IntraParietal area; DES, Direct Electrical Stimulation; DIPSA, dorso-anterior intraparietal sulcus; IPL, Inferior Parietal Lobule; IPS, IntraParietal Sulcus; phAIP47, putative human homolog of monkey AIP; PostCG, post central gyrus; PPC, Posterior Parietal cortex.

N, number; EOR, extent of resection; LH, left hemisphere; LR, right hemisphere; LGG, lower grade gliomas; LF, low frequency; HF, high frequency.

## 4 Discussion

### 4.1 Query 1: Non-human primates

#### 4.1.1 Anatomical substrates of lateral grasping network in monkeys

The classical paradigm for monkey prehension network (namely the “lateral grasping network”) was initially framed on parallel cortico-cortical pathways connecting neurons of the posterior parietal (PPC) and frontal cortex exhibiting similar architecture and functional responses. The ventral premotor area F5 is one of the most relevant areas in object motor grasping and receives inputs from the inferior parietal lobule (IPL) areas, namely AIP, PF, PFG and SII, projecting to the primary motor cortex (F1) and spinal cord (especially F5p; di Bono et al., 2017). On the medial frontolateral convexity, the rostral part of area F2 (F2vr) directly integrates grasping control, specifically during wrist rotation and orientation toward a target. However, it is also involved in reaching, although weaker evidence is available (Dum and Strick, 2002; Gamberini et al., 2009; Hecht et al., 2013). Imaging-based studies investigating the role of F3 (proper-SMA in monkeys) and F6 (pre-SMA area) in primates are less reported: primary evidence from single-neuron studies advoked the involvement of F6 in learning, execution of sequential motor behaviors and the initiation of conscious action. Accordingly, F6 neurons were reported preferentially activated while learning a sequence of actions compared to subsequent execution of the learned scheme in a behavioral study on monkeys (Nakamura et al., 1998). Based on these findings, it is widely accepted that area F6 controls action time and appropriateness.

Several prefrontal cortex areas are connected to the premotor and parietal cortex and areas 46 (46dr and 46d, 46vr and 46v) and area 12r are the most represented on dorsal and ventral banks of the principal sulcus (Gerbella et al., 2013; Saleem et al., 2014; Bufacchi et al., 2023). In a recent study, Luppino et al. reported that prefrontal areas might be subdivided into three caudo-rostral strips according to their primary output connectivity. From rostral to caudal, the first strip carries intrinsic lobar connections within the prefrontal cortex, connecting the frontal pole and orbital prefrontal areas (Borra et al., 2011; Saleem et al., 2014). The caudal part shows significant connections with the lateral intraparietal cortex (LIP), frontal eye field (FEF), supplementary eye field (SEF) and other subcortical nodes involved in gaze control, while the intermediate cluster is mostly interconnected with premotor, parietal areas and subcortical structures involved in grasping and reaching movements (Gerbella et al., 2013; Saleem et al., 2014; Borra et al., 2017). The ventral prefrontal areas (VLPF) share a common connectivity with the temporal lobe, suggesting a major role in processing object semantic features, while the dorsal prefrontal cortex (DLPF) is strongly connected to the parietal and premotor areas, as area 46 is involved in motor and behavioral control of grasping movements (Goldman-Rakic, 1987; Hecht et al., 2013).

The most reported parietal nodes of the grasping network are the anterior infraparietal cortex (AIP) and the adjacent area, PFG, both dense in neurons encoding grasping movements execution (Hyva¨rinen, 1981; Taira et al., 1990). Dorsally, MIP and the dorsal part of area V6A (V6Ad) were later attributed to reaching control (Andersen et al., 1997). The former have dense interconnections, except PFG, which only shows selective connectivity with AIP. The latter also receive input from higher-order associative visual areas in the temporo-occipital cortex (Rozzi et al., 2006). Overall, this sector mediates visuomotor transformation for reaching and grasping, processing target features (size, orientation, position and shape) and activating appropriate potential motor actions. This process, called “affordance extraction, “elicits objects’ visual properties transformation and projection through the dorsal stream (Sakata et al., 1997).

Finally, the inferior temporooccipital and the posterior occipital regions define the “ventral visual stream” involved in visual information processing, object identification and semantic recognition (Tanaka et al., 1996). Nelissen et al. identified cortical activations during action observation at the level of the upper (superior temporal polysensory area or STP) and lower banks (Tea/m sector) of the superior temporal sulcus (STS). STP is a high-order multisensory sector integrating information encoded in multiple sensory modalities, also populated by visual neurons encoding self-produced and external motion features connected to the PFG area in parietal lobe (Baylis et al., 1987). Tea/m sector was identified as a ventral visual node specialized in three-dimensional object and action processing (Orban and Caruana, 2014). Information encoded in STS is projected through Tea/m to AIP and the mirror system in the parietal lobe, providing input of action goal performed by others, intrinsic information to identity the target object as a substrate for affordance extraction, but also along STP-PFG pathway to elaborate the intention and the goal of the observed action (Jellema et al., 2000). An additional area in the parietal operculum, SII area, hosts visual-responsive neurons firing during external action observation, suggesting that it might interact with temporoparietal projections as a multisensory integrating node for motor control and action recognition (Hihara et al., 2015).

#### 4.1.2 Functional modeling of the grasping network in monkeys

The dorsolateral and dorsomedial pathways of the lateral grasping network are essential for sensorimotor integration (i.e., planning and online control) during reaching, grasping, and gaze control (Rizzolatti et al., 1998; Andersen and Buneo, 2002). According to this classical model, a dorsolateral pathway encodes grasping and different grip features, while a dorsomedial pathway encodes reaching and control of the transport/lifting phase (Jeannerod et al., 1995; Caminiti et al., 1998; Culham et al., 2003).

The dorsolateral pathway comprehends AIP (Murata et al., 2000) and subareas F5p/F5c of the ventral premotor cortex (PMv; Murata et al., 1997; Rizzolatti et al., 1998; Nishimura et al., 2007; Nelissen and Vanduffel, 2011; Nelissen et al., 2018). Similarly to AIP, PFG area responded to hand movements observed but even without a target or the presentation of 3D objects, implying a pivotal role in grasp motor scheme planning. Of note, an additional neural category responding to passive viewing of actions performed by others and peri-personal space awareness in PFG (I.e. “mirror neurons”) was characterized (Rozzi et al., 2008; Hopkins et al., 2010; Hecht et al., 2013; Fiave et al., 2018; Nelissen et al., 2018). A core description of the extended mirror network is beyond the aim of the current review (for a review, see Rizzolatti and Craighero, 2004).

AIP was proposed as primer of grasping response by activating visual-dominant neurons, which extract 3D object characteristics and propagate them to F5 visuomotor neurons encoding congruent motor representations for the affordable object. To integrate the modulation of the prefrontal cortex to the intended behavior, Arbib and co-workers proposed the “Fagg–Arbib–Rizzolatti–Sakata (FARS) model” (Fagg and Arbib, 1998; Arbib and Mundhenk, 2005). AIP produces multiple motor representations of object affordances to F5, while modulated by prefrontal inputs, encoding the goal of the individual in affording the target object; this whole process selects the most appropriate motor execution program. The robustness of this model relies on the fact that F5 is not directly interconnected with the inferotemporal cortex but receives dense connections from the ventral prefrontal cortex, which in turn receives input from inferior and posterior temporal areas, as previously described.

The dorsolateral pathway encoded the transformation of intrinsic target properties into appropriate behavioral and motor commands comprehending hand pre-shaping, force adjustment and type of grip during visually-aided grasping (Jeannerod et al., 1995) through visuomotor neurons in AIP, PFG and F5, with significant activation while observing a graspable object or performing a grasping task (Bonini et al., 2014). Lesion studies within this pathway clarified how lesions within AIP and F5p affected hand pre-shaping and wrist orientation, leaving object-reaching ability mostly unaffected. These findings were consistent only during precision grip of small objects, while whole hand prehension showed no deficit, confirming the crucial role of AIP and F5p during complex sensorimotor control or pinching of small objects (Ehrsson et al., 2001; Cavina-Pratesi et al., 2007; Grol et al., 2007; Begliomini et al., 2007a, 2014; Renzi et al., 2013; Monaco et al., 2015). Neighbor area F5c (F5 subarea) lesion, despite having the same visuomotor properties as other F5 subareas, was not responsible for any grasping impairment (Fogassi, 2001; Bonini et al., 2014).

The selection of the object’s meaning also relies on connections from prefrontal area 12r, while the behavioral response based on the overarching goal could be appointed to prefrontal area 46v, which is densely connected with posterior parietal and premotor cortex. Eventually, the affordance selection elicits F5 motor representation, activating F1(the primary motor area). Once affordance is selected and hand shaping programmed, additional features modulate arm-hand movement and grip characteristics. SII region, for example, was activated during movement, especially object grasping and different hand configurations, object orientation and passive view: it is plausible to consider this area as a fundamental sensorimotor integration node for object’s physical and visual properties during reaching and grasping, receiving feedback information used in F5, AIP and PFG for online monitoring and update grasping motor scheme (Hihara et al., 2015).

The dorsomedial pathway connects the PPC, among all V6a (Bosco et al., 2010), VIP and MIP (Johnson et al., 1996) with F2 anteriorly, within the primate dorsal premotor cortex (PMd), and associative visual areas posteriorly (Nishimura et al., 2007; Nelissen and Vanduffel, 2011; Fiave et al., 2018; Nelissen et al., 2018). MIP and AIP are part of both ventrolateral and dorsomedial pathways, confirming the integration and overlap of the two pathways. Visual areas V1–V4 are strongly connected to V6A and, in particular V1 and V2 subareas, are involved in the representation of peripheral visual field showed the most substantial convergence onto V6A, with similar attributes toward peripheral vision and stimulus position in craniotopic coordinates encoding (Daniel and Whitteridge, 1961; Galletti et al., 1999). Despite previous modeling including the precuneus in the dorsomedial stream only, a substantial dorsomedial and dorsolateral pathways overlap was later identified, whose direct function in reaching and grasping remains only partially understood. Tracer studies in monkeys revealed projections to both area F5 and V6A, suggesting a role in coordinating reaching and grasping, in addition to the well documented activation during bimanual performance, complex tasks and (Johnson et al., 1996) as a functional hub in the default mode network (DMN) in monkeys (and humans); however, this area of grasping-reaching overlapping does not seem to correspond with the DMN activation sites in fMRI (Johnson et al., 1996; Fransson and Marrelec, 2008; Mantini et al., 2011). Of note, the frontal eye field (FEF, area 8 m) area is also strongly connected to the precuneus, suggesting a role during sensory guidance of limb and hand movements as much as a hand-eye coordination node (Thompson et al., 1996).

Overall, an integration gradient on the functional level between the dorsomedial and ventrolateral streams seems consistent. Although the ventrolateral stream is primarily integrated with somatosensory areas and the dorsomedial stream the has more robust interconnections to the visual cortex, several areas within each pathway are strongly interconnected. Grasp planning and execution revealed significant activation in the IPL convexity areas posterior to AIP, comprehending area PF, PG, and particularly area PFG, having direct connections to F5 (Bonini et al., 2011, 2014). Similarly, neural activity during grasping was recorded in V6A and PMd subareas (i.e., area F2vr; Fattori et al., 2010, 2012). Conversely, subpopulations of F5 and AIP showed reaching-related coding in additional studies (Lehmann and Scherberger, 2013), supporting an overlapping architecture of the lateral grasping network (Battaglini et al., 2002).

#### 4.1.3 Cortico-subcortical loops integrated with the lateral grasping network in monkeys

The cerebellum and the basal ganglia play a role in motor cognitive tasks execution and control ranging from sensorimotor to complex behavioral integration (Strick et al., 2009; Bostan et al., 2013; Caligiore et al., 2013). They fulfill a functional architecture of input projections from a wide range of cortical areas, despite earlier findings supported the idea that these connections were anatomically and functionally segregated according to the function being modulated (Middleton, 2000; Baldassarre et al., 2013). Hoshi et al. reported a strong bisynaptic connection between the striatum and the thalamus, while Bostan, Dum and colleagues showed that the subthalamic nucleus has similar connections to the cerebellar cortex through the pontine nuclei (Hoshi et al., 2005; Bostan et al., 2010). Tracer studies in monkeys clarified that efferent connections from the cerebellar nuclei are projected to M1 but also on premotor, prefrontal and parietal areas (Percheron et al., 1996). The output streams to M1 and premotor areas within the dorsal dentate nucleus are clustered in a circumscribed “motor” domain while a ventral region of the nucleus showed connections to the prefrontal (non-motor) areas (Shinoda et al., 1992; Dum and Strick, 2003). This functional segregation in the dentate nucleus has a counterpart in the cerebellar cortex. The dorsal “motor” dentate is majorly connected to the anterior cerebellar lobe (lobules III–VI) and paramedian lobule (HVIIB and HVIII), while the ventral dentate is connected with the posterior vermis and the cortex are not involved in motor control, as consistently documented also in imaging studies (Kelly and Strick, 2003). The cerebellar cortex elicits activation of the dentate nucleus, and this functional loop allows cortical areas (i.e., prefrontal, premotor and F1) to interconnect with the dentate nucleus via the pontine nuclei while areas not projecting to the cerebellum showed no major connections from the cerebellum (i.e., area 46v, 12 and TE, for example). This series of closed-loop circuits might represent the anatomical substrate of a multi-level modulation system involving the cerebellum as a significant player. If so, cerebellar output directly influences multiple non-motor cortical areas (i.e., premotor and parietal cortex), suggesting an essential role in various cognitive tasks (Koziol et al., 2014). Similarly, motor and non-motor subregions of STN showed a similar projection onto the motor and non-motor cerebellar cortex (Bostan et al., 2010), while Chen et al. demonstrated that the cerebellum is responsible for a short-latency direct modulation of the striatum (Chen et al., 2014). Although the growing evidence hereby reported, the physiology of cerebellar-striatal interconnections is still poorly understood in contexts other than motor functioning, and further research is prompted.

### 4.2 Query 1: Human primates

#### 4.2.1 Anatomical and functional substrates of the object-oriented hand manipulation network

The anatomical and functional substrates of dexterous motor behavioral responses, complex non-verbal communicative gestures and abstract manipulative tasks in humans—comprehending those abilities defined as “praxis”—represents the translational evolution of the pre-existing dual streams frontoparietal network (i.e., lateral grasping network) defined in the macaques. With a certain amount of approximation, we can refer to it as “praxis representation network” (PRN) in the human model.

The premotor and prefrontal areas play a significant role in the dorsoventral and dorsomedial pathways for reaching and grasping performance within the network. The human ventral premotor area (PMv or hPMv) has been proposed as the homolog of the rostral part of the nonhuman primate F5 area, with which it shares direct or indirect control of hand movements and mirror-like properties (Cerri et al., 2003, 2015; Ehrsson et al., 2007; Maranesi et al., 2012; Fornia et al., 2018). A recent rTMS study highlighted PMv features by reproducing an impairment of current finger position during precision grip, confirming its involvement in such a task (Davare et al., 2009). As mentioned above about area F2 in monkeys, the dorsal premotor area (PMd) is involved in grasping and reaching tasks, confirming that the dorsomedial frontoparietal circuit serves both grasping and reaching encoding (Raos et al., 2006; Errante and Fogassi, 2019). Indeed, the inactivation of PMv impaired hand pre-shaping, while that of PMd corresponded to interference in object lifting with disruption of grasping-lifting coupling, conforming a different but convergent contribution of these areas in controlling hand/arm performance during grasping, as previously demonstrated in monkeys (Davare et al., 2006; Fornia et al., 2020b). Comparable with animal findings, additional areas involved in hand motor control are the supplementary motor areas (proper- and pre-SMA) and ventrolateral and dorsal prefrontal cortex (VLPFC and DLPFC, respectively). Several imaging studies investigating planning vs. online control of hand motion toward a target attributed a relevant inhibition and behavioral switching control to pre-SMA, DLPFC (Spraker et al., 2009) and VLPFC (Mostofsky et al., 2003; Aron, 2009; Glover et al., 2012; Tabu et al., 2012), comprehending force strength adaptation and dynamic grip modulation (Neely et al., 2011) to grasped objects (Kuhtz-Buschbeck et al., 2001; Holmström et al., 2011). Most of the studies we reviewed on healthy humans collected evidence on dominant hand grasping execution in right-handed subjects with a skew lateralization of cortical activity measured during handgrip only for the involvement of contralateral sensorimotor cortex (and ipsilateral superior cerebellum). Nevertheless, most studies consistently reported bilateral activation of PMv, PMd, SMA, cingulate motor cortex, IPL, insular, vermis and both superior/inferior cerebellar hemispheres, despite previous reports left-lateralized cortical activations in right-handers humans but a widespread bi-hemispheric sensorimotor, premotor and SMA activations in left-handed candidates performing grasping tasks with the dominant and—even more evident—non-dominant hand (Grafton et al., 1996; Ehrsson et al., 2000, 2001; Kuhtz-Buschbeck et al., 2001; Ward and Frackowiak, 2003; Cavina-Pratesi et al., 2007; Vaillancourt et al., 2007; Matsuda et al., 2009; Kurniawan et al., 2010; Fiehler et al., 2011; Hong and Jang, 2011; Martin et al., 2011; Neely et al., 2011; Glover et al., 2012; Makuuchi et al., 2012; Nathan et al., 2012b; Begliomini et al., 2014; Fabbri et al., 2014, 2016; Plata et al., 2014; Monaco et al., 2015; Leo et al., 2016; Przybylski and Króliczak, 2017; Ariani et al., 2018; Cavina-Pratesi et al., 2018; Styrkowiec et al., 2019; Marneweck and Grafton, 2020; Sulpizio et al., 2020; Bencivenga et al., 2021; Errante et al., 2021b; Michalowski et al., 2022). These results must be interpreted in light of several methodological and biological factors: Milner et al., for example, reported that younger participants showed greater deactivation of ipsilateral M1 during hand grip compared to elderly volunteers in both dominant and non-dominant limbs (Milner et al., 2007).

The parietal lobe comprehends areas specialized in encoding and modulating reach- and grasp-related fine hand movements: human medial parietal sulcus (mIPS) and superior parieto-occipital cortex (SPOC) have been proposed as homologs for V6 area complex found in macaques (Pitzalis et al., 2013). Recent studies confirmed the specific role of SPOC area in visually-aided reaching movements (Gallivan et al., 2009; Cavina-Pratesi et al., 2010): its activation during reaching subserves subject hand preferences and stimulus location in peripersonal space. Indeed, left-handers commonly employ both hands when asked to reach and grasp a target object, while right-handers showed a segregation toward a dominant hand use. This preference is also extended to the peripersonal workspace within range of action: right-handers respond with higher SPOC activation when the object is presented within range of their dominant limb, while left-handers exhibit response to objects within range of both sides. Irrespective of hand preference, bilateral SPOC and left precuneus showed visual field polarization, activating most when the object was presented in the inferior visual field. The anterior precuneus (aPCu) is significantly activated during both visually-aid and reaching in the dark, suggesting it does not act as an associative visual area but modulates both visuomotor and sensorimotor input transformation for reaching execution (Filimon, 2010; Gatti et al., 2015). Within this dorsomedial parietal region, the presentation of affordable objects in different visual field sectors elicit reproducible selective activations, not discordant from what is observed in animals: mIPS—which is strongly interconnected with caudal PMd through the dorsomedial stream, is more activated during object reaching execution within the central visual field, while a wider bilateral perieto-occipital junction (POJ) area is elicited during peripheral vision engagement. The latter showed selective connectivity to the rostral PMd (Prado et al., 2005).

In homology with animal models, ventrolateral parietal areas are involved in grasping encoding and performance, namely the rostral part of the lateral bank of IPS (aIPS) and the supramarginal gyrus (SMG; Frey et al., 2005; Culham and Valyear, 2006; Filimon, 2010). An additional area in the superior parietal lobule (SPL) seems related to grasping encoding but overlaps with SPOC, precuneus and mIPS areas. All these share activation signals with PMd in fMRI studies (Tunik et al., 2008; Gallivan et al., 2011b; Fabbri et al., 2014) and co-activate during complex 3D object haptic manipulation (either with and without proper grasping task), together with the right SPL, aIPS, anterior SMG (aSMG) and area SII in the parietal operculum (Jancke, 2001). The parietal opercular region comprehends peculiar associative somatosensory areas, namely OP1 and OP4, as extensive components of the “human grasping/praxis network” (Eickhoff et al., 2006, 2007). According to fMRI data, OP1 and OP4 are concurrently activated during tactile hand stimulation, motor execution and are related to fine object manipulation for target recognition with and without visual aid (Eickhoff et al., 2007; Burton et al., 2008). Further TMS evidence confirmed their involvement in haptic working memory during object identification in the darkness and grasping motor scheme programming. In light of the previous evidence, it has been proposed that OP1 and OP4 are the human homologs for parietal area SII in macaques (human SII).

A connectivity gradient is present in the parietal areas related to the object manipulation network: more dorsal cortical areas – showing higher connectivity with posterior visual areas – might be involved in special processing of visual input integration for action planning when the target object is presented outside the range of action. On the contrary, parieto-ventral areas activation might imply remapping of motor behaviors encoding peripersonal space affordance depending on other than visual sensorial feedback, confirming the consistency with the lateral grasping network model described in monkeys and its dual streams partial segregation (Gallivan et al., 2011a; Renzi et al., 2013; Rossit et al., 2013; Monaco et al., 2017).

The cortico-cortical connections between frontal nodes and PPC within the network consist of long-range association bundles, namely the superior longitudinal fasciculus (SLF) and the arcuate fasciculus (AF) bilaterally. Both injective tracers in monkeys and non-invasive imaging techniques in both species described the anatomical substrate of this densely interconnected network. Probabilistic DTI tractography, among all, permitted an accurate virtual definition of the dorsal stream bundles anatomy, comprehending the classification of its subdivisions (SLF I, II, III) in the living human brain. According to Martino et al., SLF can be anatomically split into three independent fascicles: SLF I connects the superior frontal gyrus and anterior cingulate cortex to SPL and precuneus posteriorly (Martino et al., 2013). SLF II binds the posterior part of the superior and middle frontal gyri and the caudal part of IPL (aIPS and AG). Finally, SLF III connects the inferior frontal gyrus (IFG-BA44, comprehending vPM) to aIPS and IPL. In the interest of simplification, it might be assumed that SLF I represents the cortico-cortical connections of the so-called dorsomedial frontoparietal pathway.

In contrast, the SLF II and SLF III represent the subcortical pathway of the ventrolateral circuit. Finally, the recent description of an oblique frontal white matter bundle, namely the frontal aslant tract (FAT) was proposed as a substrate for the interconnectivity between PMv/IFG and pre-SMA/proper SMA, which are consistently associated with activation related to high-order motor cognition and motor response inhibition (Nachev et al., 2008; Catani et al., 2012; Rojkova et al., 2016).

The temporo-occipital regions in humans and macaques carry structural and functional discrepancies, which complicate a direct comparison between the species, as a direct topographical correspondence is unreliable, given the greater representation of highly associative temporo-occipital areas in the evolution of *Homo sapiens* compared to primates. Nevertheless, several fMRI studies located probable homologs: the lateral occipital cortex (LOC) is located ventral to the human homolog motion-sensitive middle temporal area (MT) within the posterior part of the inferior temporal (ITG) and fusiform gyrus (FG). LOC is activated during visual processing of shape, faces, action identification and object dimensions relevant to grasping goals among other functions (Monaco et al., 2011); the latter proves LOC to be the human homolog of area TEa/m and surrounding sectors in macaques (Malach et al., 1995; Bell et al., 2009). FMRI studies described the co-activation of AIP, PMv and LOC during object-oriented action planning, with specific temporospatial patterns of activation correlated to hand activation schemes in MVPA analyses (Gallivan et al., 2013). Moreover, LOC elaborates visual and haptic object representations, defining a multimodal object identity, which is further processed in hAIP (Verhagen et al., 2008).

The human homolog of macaque area STP is rostral to MT and dorsal to LOC in humans: it comprehends a broad region within the posterior superior temporal sulcus (pSTS) and middle temporal gyrus (MTG; Allison et al., 2000). While LOC activates more during visualization of different object configurations, pSTS/MTG sector is more related to the kinematic features of target actions and is implicated in the human mirror network as a visual action information processing node (for additional information, see Caspers et al., 2010; Rizzolatti et al., 2014).

Similarly, the exploration of unusual object shapes activates foveal cortex even when shapes have been explored with haptic feedback in the dark, while retinotopic V1 cortex activates when shapes are visualized, eliciting neural activity within the cortical location of the target in the visual field during observation. These findings imply that storage of object perception depends on sensorial modality employed to explore the object itself at the first exposure. Still, tactile hand exploration consistently reactivates early visual cortex (EVC), LOtv, aIPS and PMd irrespective of the sensory modality implemented during the first exploration. This suggests that these areas collect an abstract representation of the object of interest, recalled even when the task is performed in complete darkness (Monaco et al., 2017). Several authors concord that action imagery cannot explain these results, and further studies are necessary to elucidate these results.

The associative temporo-parieto-occipital fibers of the IPL to the posterior STG, MTG and ITG in the human brain are abundant and represented mainly by the posterior segment of the AF or the posterior vertical segment of SLF according to the description by Catani and colleagues. However, an additional pathway interconnecting SMG and STG is carried by the middle longitudinal fasciculus (MdLF; Catani et al., 2005; Martino et al., 2013; Makris et al., 2017). The latter might reproduce the counterpart Tea/m-AIP and STP-PFG connectivity previously described in macaques, confirming the role of these areas in the grasping/action recognition network.

The long-range direct prefrontal-inferotemporal connectivity wire is still a matter of debate. In humans, the inferior fronto-occipital fasciculus (IFOF) might be responsible for direct connectivity between prefrontal areas (i.e., MFG) and LOC, resembling the inter-connectivity documented in macaques between VLPF and the inferotemporal region. Notably, the internal long direct segment of the AF described by Catani et al. represents an additional inferotemporal-prefrontal dorsal connection with no homolog in primates’ brains (Catani et al., 2005; Rilling et al., 2008). Despite evidence from diffusion imaging studies and intraoperative findings during awake surgery attributing this role to the IFOF, there is no such unequivocal evidence to exclude the involvement of AF.

#### 4.2.2 Cortico-subcortical connections and network nodes in humans

Beyond cortico-cortical connections elicited during motor programming and performance of reach-to-grasp and manipulative actions generally included in the network definition, additional subcortical nodes are recognized to play a relevant modulating role. The striatum represents the primary output node within the basal ganglia, and its activation has been measured during hand movements: it receives afferents from frontal, parietal and temporal cortex areas and relays them on the thalamus and brainstem and onto the pallidum for backpropagation to the cortex (Haber, 2003). The frontal lobe connections constitute an ensemble of segregated functional fields with a high degree of overlap documented in humans. Evidence from previous investigations highlighted that the orbitofrontal cortex (OFC) and medial prefrontal cortex (MPFC) – involved in motivational content information for decision-making purposes-connect with the ventral striatum (Kurniawan et al., 2010), while DLPFC projections were found within the central striatum overlapping with OFC-PCFC fields and the dorsolateral portions of putamen and caudate, interconnected with premotor and motor areas (Draganski et al., 2008). The ventral striatum is involved in motivational context analysis for motor performance, while the dorsolateral striatum in pure motor control with substantial overlapping relays among these two nodes (Alexander et al., 1990; Francois et al., 1994; Saleem et al., 2002; Pope et al., 2005; Lehéricy et al., 2006; Vaillancourt et al., 2007).

Furthermore, basal ganglia are co-activated during motor tasks, not strictly requiring a reach or grasp goal but only manipulation of tridimensional objects under visual or haptic sensory guidance. FMRI investigations proved that the Putamen, the Caudate nucleus (CdN), Globus Pallidus (GP) and STN are involved in performing complex hand tasks requiring a precision grip compared to whole hand movements. Marangon et al. found a specific bilateral activation of the putamen and GP during such tasks (with more intense activation peaks in the contralateral hemisphere to the performing hand, however), as later confirmed by Errante and Fogassi during the execution of a skilled manipulation task compared to simple finger tapping (Marangon et al., 2016; Errante and Fogassi, 2019). The subthalamic nucleus (STN) has been investigated with single unit activity recording in patients undergoing deep brain stimulation (DBS) surgery, confirming the involvement of this nucleus in grip force control, suggesting that the basal ganglia might modulate grip properties during grasping tasks, in line with fMRI findings (Vaillancourt et al., 2004; Grafton, 2010; Grafton and Tunik, 2011). The basal ganglia, co-activated with cortical sites, may prosecute the spatiotemporal representation of the hand during object manipulation. Moreover, basal ganglia control movement sequence programming and are involved when a specific sequence of manipulative movements has to be coordinated for a goal-oriented action (Lehéricy et al., 2005; Garr, 2019).

The ventral thalamic nuclei work as relay units for basal ganglia output pathways following a semi-segregated topographical and hierarchical structure discussed before (Hoover and Strick, 1993; Matelli and Luppino, 1996). As reported for frontostriatal connectivity, an analog convergence among thalamic output fibers toward the frontal cortex was demonstrated: medial dorsal (MD) nuclear projections, for example, are primarily directed toward MPFC and OFC areas while ventral anterior (VA) nuclear projections to PMv and PMd, with a coherent overlapping field of nuclear areas projecting to both MPFC-OFC and premotor areas and similarly to both premotor areas and M1. An integrative role for thalamic nuclei has been postulated in the past, especially considering that cortico-thalamic connections outnumber the thalamocortical projections by several orders of magnitude, making it counterintuitive that ventral thalamic nuclei merely relay information back to the cortex (Sherman and Guillery, 1996; Darian-Smith et al., 1999). This hypothesis is also supported by the identification of direct projections between the thalamus and striatum, as proposed by McFarland and Haber and later reproduced in diffusion tractography. The latter confirms the dual (relay-overlay) role of ventral anterior and ventrolateral thalamic nuclei through their vast connections with cortical, striatal and pallidal regions (Behrens et al., 2003; Johansen-Berg et al., 2005). According to this anatomical evidence, it has been proposed that they project but also integrate motor information with dorsal striatal output, which in turn receives direct input from a larger spectrum of cortical areas, contributing to modulate features of motor programming and online control during dexterous motor tasks (i.e., limb transport, grasp and fine object manipulation).

Finally, the cerebellum plays an adjunct role in motor control of reach-to-grasp movements and hand manipulation tasks in humans: it contains a somatotopic motor map of the hand within lobules IV, V and V mainly, and several virtual lesion studies described disturbance of either reaching, grasping or object manipulation (Nitschke et al., 1996; Rand et al., 2000; Grodd et al., 2001; Zackowski et al., 2002; Holmström et al., 2011). Milner et al. and Errante and Fogassi reported similar activation in the anterior and posterior cerebellar cortex within lobules V, VI and VIII-VIIIb during complex hand manipulation tasks with the dominant hand, confirming previous evidence by Schmamann et al. about the somatotopic representation of both distal arms in the cerebellum for hand manipulation performance (Milner et al., 2007; Errante and Fogassi, 2019; Stoodley et al., 2021). Recent imaging studies collected evidence of reported activation within the dorsal and ventral sectors of the dentate nucleus (DN), the main cerebellar output node to the thalamocortical pathway (projecting to parietal and premotor areas (Dimitrova et al., 2006): DN might play a role in voluntary movement correction, irrespective of the presence of sensory (visual, haptic and others) feedback (Weeks et al., 2000). Similarly to what was demonstrated in non-human primates, anteroposterior cerebellar segregation in the nucleus interpositus (IN) and DN regarding the hand-arm representation seems conceivable, with hand skills encoding being activated more anteriorly while limb transport and lift encoding (i.e., reaching) are more posteriorly represented (Mason et al., 1998).

### 4.3 Query 2: Intraoperative translation

The description of standard DES protocols for motor and cognitive mapping is beyond the aim of the current review (for additional information, see Duffau, 2021; Rossi et al., 2021).

Brain mapping has a centenarian history, starting more than a century ago with the pioneering studies on monkeys by Sherrington, who first described the organization of the Rolandic cortex using DES (Sherrington, 1906). About 30 years later, Penfield and Boldrey demonstrated a somatotopic segregation of the sensory-motor system in humans during awake surgeries in patients with brain tumors (Penfield and Boldrey, 1937). The stimulation of the sensorimotor cortex showed the existence of a “body shape-like” distribution of motor responses for the face, upper and lower limb (i.e., the “Penfield homunculus”) from lateral to medial near the central sulcus. Later evidence suggested a revisited somatotopic organization of the precentral gyrus with different stimulation paradigms: Roux et al. confirmed a medio-lateral gradient for positive motor sites in patients with intact motor systems (Roux et al., 2020). He demonstrated a substantial inter-subject consistency for eliciting simple or stereotyped movement of wrist, hand, global or individual fingers other than oro-facial muscles (evaluated as behavioral responses with no EMG recording) applying a low frequency bipolar DES on the precentral gyrus. These findings corroborate what described by Fornia and colleagues, who described an organized medio-lateral somatotopy in highly elicitable sectors within M1 but a more heterogeneous distribution of positive sites in the premotor cortex with longer response latencies and overall reduced excitability during high frequency DES and EMG recording. Accordingly, the authors presented evidence for a “transition oro-hand zone” localized in the ventrolateral premotor cortex, where output contraction of multiple muscles was more represented compared to M1 (Fornia et al., 2018). A similar concept has been proposed for the somatosensorial cortex and sensorimotor pathway: however, these conclusions are still a matter of debate (Dejerine, 1895; Duffau et al., 2003; Roux et al., 2018). Relevant similarities across the species have been documented also in the motor system sub-structure. Rathelot et al. first described a segregation within primary motor area in macaques according to intrinsic characteristics in terms of excitability during direct electrical stimulation; indeed, M1 is clustered in a caudal sector exhibiting higher excitability (“new M1”) compared to its rostral sector (“old M1”). Most corticomotoneuronal fibers project from new M1 and are fast-conducting projections, while old M1 originate a smaller proportion of corticospinal fibers with lower conductive properties (Rathelot and Strick, 2009).

Viganò et al. reported heterogeneous responses during DES motor mapping over hand knob area in patients undergoing surgery for brain tumors; a rostrocaudal gradient within the hand-knob region was described (the caudal sectors showed higher excitability when compared to the rostral ones; Viganò et al., 2019). This suggests a distinct contribution of these areas to the corticospinal tract. The rostral hand-knob might correspond to the monkeys’ old-M1 (however, this interpretation cannot be supported by architectonical data in the same patients) or, alternatively, to a motor transition area between M1 and PMd, explaining its lower excitatory profile and coherently with fMRI data showing a partial overlap of the premotor cortices and M1 on the convexity of the PreCG.

Even though the consistency of the previous evidence, the continuous somatotopic homunculus has been questioned since then and several methods suggested that M1 is interrupted by regions spreaded around isolated effector-specific (foot, hand, mouth) motor areas with a distinct connectivity pattern, structure and function. Gordon and colleagues recently described the coexistence of an effector-specific circuit constituted by concentric M1 sectors for precise, isolated movements of tongue, fingers and toes for dexterous movement and speech, and a second integrative system, namely the “somato-cognitive action network (SCAN),” interconnecting the motor effector sites and the cingulo-opercular network (CON) for whole body movement planning, neurovegetative preconditioning control, arousal, error correction and pain response among others (Gordon et al., 2023). According to these assumptions, the regions for foot, hand and mouth fine motor movements are, in fact, somatotopically-oriented with concentric architecture (distal appendices at their core, and proximal structures along the perimeter), while the inter-effector sectors coordinates motor-specific areas with the CON to execute whole-body performances.

Exploring behavioral responses during DES stimulation of premotor areas, Penfield and Jasper identified sites (“negative motor areas”) of motor arrest without loss of consciousness (negative motor responses, NMRs) located in the posterior part of IFG (likely PMv) and pre-SMA (Luders et al., 1987; Penfield, 1954). Several modern studies investigated the functional cortical and subcortical anatomy of sites responsible for NMRs (Schucht et al., 2013; Rech et al., 2014, 2019; Monticelli et al., 2020). The superior frontostriatal tract (sFST), running from SMA to the caudate head, might play a role in the motor control network, together with the frontal aslant tract (FAT), which connects pre-SMA and IFG; the latter, however, would be more relevant in the face and speech motor initiation and control, following the rostrocaudal somatotopy within the pre-SMA area (Fontaine et al., 2002; Catani et al., 2012; Rech et al., 2016, 2019, 2020). Confirming the essential role in motor control (comprehending initiation and inhibition to move) within the dorsomedial stream of object-oriented hand manipulation network, Rech and colleagues proved that the preservation of these cortico-subcortical motor connections could prevent permanent motor and hand-coordination deficits, even though transitory speech and motor disturbances were experienced in the early postoperative period (Rech et al., 2014). The transient neurological impairment was reported as motor and language initiation dysfunction, bimanual coordination (Rech et al., 2014) and fine movement deficits during the first weeks after surgery (Rech et al., 2017). From a functional point of view, the elicitation of negative motor responses bears a significant limitation, as the net effect of DES on cortical surface is not entirely understood (Borchers et al., 2012). Indeed, some authors attribute to DES an inhibitory role on motor performance, while others assume that the behavioral inhibitory response is an epiphenomenon given by the perturbation of a positive ongoing motor scheme. The real contribution of SMA/pre-SMA to motor scheme execution during reach/grasp or dexterous hand manipulation tasks and the mechanism underneath is still debatable. This area has also been studied in terms of compensatory reserve after nearby resection for brain tumor removal: Rosenberg et al. reported that patients with solid preoperative fMRI activation within lesioned SMA during a motor task were less likely to experience transient disruption during DES in this area as a consequence of a possible “higher functional reserve” in the adjacent regions able to compensate for the resected portion (i.e., suggesting SMA area could be resected entirely; Rosenberg et al., 2010). Also, they found higher connectivity of the lesioned SMA with other ipsilateral and contralateral cortical regions during fMRI acquisition, ipsilateral M1 and contralateral SMA, among others. When such coupling was absent, patients were more likely to experience transitory functional deficits during DES (suggesting SMA was still functional and wide resection would have induced severe SMA syndrome), driving surgical considerations on extending the resection. However, contralateral SMA activation might also be a clue of lost transcallosal inhibition from the damaged frontal lobe or even indicate that an insufficient compensatory mechanism is in act and some marginal deficit is already present preoperatively. Although contralateral SMA recruitment is associated with functional compensation and faster recovery in stroke patients, SMA functional reorganization in patients with brain tumors and DES-induced SMA functional disruption during awake mapping is poorly predictable based on fMRI data and further studies are demanded (Shimizu et al., 2002; Krainik et al., 2004; Rosenberg et al., 2010).

Altogether, the previous results find a sufficient analogy to animal studies, suggesting that the phenomena occurring during DES in awake conditions are results of perturbating those very functional substrates homologs implicated in object-oriented complex hand motor tasks in monkeys. The previously mentioned DES protocols are widely accepted as the gold standard in motor mapping, with the potential benefit of preserving functional integrity in patients undergoing awake or asleep surgery according to the technique employed. Nevertheless, motor cognition is frequently affected after surgery in the perirolandic area, with a prevalence of post-operative ideomotor apraxia around 30% (Rossi et al., 2018) but specific mapping techniques are far from becoming the standard of care in the neurosurgical practice. Rossi et al. recently proposed a newly developed ecological intraoperative task, namely the hand manipulation task (HMt; Rossi et al., 2018): it consists of a hand-object interaction trial with a small cylindrical handle mounted on a rectangular base resembling a worm screw. The patients were asked during awake motor mapping to grasp the handle with thumb and index finger, hold, rotate and release the object with no visual clue (i.e., haptically driven task), while DES was intermittently applied on regions of interest under EMG monitoring. The authors reported peculiar behavioral and electrical response patterns consistent with previous knowledge of the frontoparietal hand manipulation network (i.e., praxis representation network): DES applied over M1 elicited a tonic hand muscle activation and cessation of handle rotation, while stimulation on S1 caused clonic activations and release of the object. DES produced over SMG and vPM were responsible for complete movement arrest without muscle activation, and a disruption of online awareness of motor execution was documented (vPM; Fornia et al., 2020a). The authors reported a lower incidence of ideomotor apraxia irrespective of the hemisphere at 5 days (28.4% vs. 71.1%) and 1–3 months (8.8% vs. 47.4%) after surgery in patients undergoing intraoperative HMt compared to those undergoing “standard” motor protocol with no significant impact on the extent of resection. The residual percentage of patients affected by long-term apraxia were affected mainly by superior parietal lobule tumors, suggesting a lower task efficiency for mapping posterior parietal sites within the frontoparietal praxis network (especially along the dorsomedial pathway). As the parietal lobe is responsible for multisensorial integration during action programming and execution, plural sensory input might be necessary to elicit an intraoperative response; in this view, haptically-driven HMt might underperform during awake mapping of SMG compared to a visual-aid dexterity task.

Moreover, Fornia et al. provided quantitative evidence of the effect of DES on premotor areas through EMG recording during the execution of the HMt (Fornia et al., 2020b). They reported a complete arrest of movement during HMt when DES was applied over vPM, characterized by a complete and sharp arrest of motor scheme execution and muscle suppression in several or even all muscles involved, especially in the dorsal vPM sector. Several sites of DES on vPM elicited also a partial impairment with execution failure described as “clumsy-like,” characterized at the EMG by a partial and variable impairment of muscle contraction. Interestingly and consistent with its direct kinematic control role, DES on dPM elicited first an early suppression followed by a progressive muscle recruitment. This response was not registered in any other premotor sector. Viganò et al., in the dorsalmost aspect of dPM corresponding to the anterior hand knob sector, reported mixed responses in terms of EMG response patterns: these ranged from complete muscle suppression to mixed suppression-recruitment phenomena with segregated responses between distal and proximal muscles (Figure 7; Viganò et al., 2019).

The authors suggested that the arrest pattern might be related to the perturbation of neural areas directly implicated in motor output execution, both in vPM and dPM. In contrast, the perturbation of vPM alone might disrupt sensorimotor integration for online control of hand movements, causing a clumsy-like behavior. Vigano et al. later reviewed this evidence to identify those subcortical frontal connections involved in the dexterous hand motor control during the performance of the HMt in awake patients (Viganò et al., 2022). The author concluded that transient perturbation of short-range premotor mid-U-shaped fibers, SLF I and II within the dorsomedial stream, the sFST and corticospinal projections of dPM and SMA was related to the complete arrest pattern during DES. At the same time, stimulation on the inferior frontostriatal tract (iFST), arcuate fasciculus and SLF III within the dorsolateral stream was preferentially associated with the clumsy response pattern. The arrest pattern in the more dorsal subcortical stream might reflect an inactivation of nodes more proximal to the motor output: in fact, SMA and dPM have direct projections to the spinal cord and both direct/indirect connections with M1 through local premotor-motor U fibers and longer striato-thalamo-cortical loops. Moreover, this hypothesis might justify the presence of short-term post-operative upper limb motor deficits after resection of the dorsal but not of the ventral frontal region.

Similar response patterns were described during mapping of the posterior parietal cortex; a medial cluster of activations sites elicited complete motor arrest as previously reported in the frontomedial cortical region, while a more lateral cluster of stimulation points evoked a clumsy-like behavioral response during HMt. Again, the arrest pattern might represent the temporary disruption of a hierarchically higher node of the frontoparietal praxis network directly (or more directly) involved in the computation of motor output. On the contrary, clumsy response pattern might indicate a disruption in sensorimotor integration necessary for any dexterous hand movement requiring haptic control. Notably, the medial cluster comprehended areas connected with the angular gyrus and SPL, while the lateral cluster with the anterior SMG (Fornia et al., 2023). Finally, the majority of stimulation sites were identified within hAIP and, secondarily, the adjacent dorsal sector in the anterior intraparietal sulcus (DIPSA), corresponding to the anterior motor-dominant and posterior visuo-dominant homologs of monkey AIP (Figure 7; Orban, 2016).

Rolland and colleagues collected additional evidence on a series of patients harboring tumors adjacent to the right IPL (Rolland et al., 2018). In their series, patients were asked to perform simple repetitive movements of the left upper limb (flexion of the arm, wrist, and fingers, then the extension of the arm with the open hand and fingers for 4 s), together with an additional test (naming test, non-verbal semantic, visuospatial etc.). Awake mapping allowed testing of complex movements, motor control during action and even bimanual coordination. The authors reported transient fine motor impairment in one patient only, with complete recovery within 3 months after surgery, achieving total or subtotal resection in 13 cases. In line with previous experiences, Rolland et al. confirmed the vital importance of preserving cortical and subcortical somatosensory pathways detected by intraoperative DES in awake conditions to prevent pure motor deficits and perirolandic fronto-parietal areas to prevent complex motor behavior impairment.

In asleep setting, a similar conclusion was drawn by Cattaneo and colleagues in a cohort of 17 patients undergoing brain tumor surgery in the parietal lobe (Cattaneo et al., 2020). They applied dual strip DES to the hand-M1 area and the parietal cortex, recording abductor pollicis brevis (APB) MEP responses after posterior parietal cortex conditioning stimulation (additional details on stimulation paradigm are available in Cattaneo et al. (2020). Their results suggest the existence of a direct parietal-motor functional connection with short-latency modulating properties, with two distinctive clusters according to their effect on motor output: a ventral region corresponding to the part of SMG immediately posterior to the inferior postcentral gyrus to the parietal operculum eliciting an excitatory effect on MEPs, and a dorsal cluster within the SPL responsible of an overall inhibitory effect on MEP output. These findings, however, are not directly comparable to those previously discussed in awake patients, as motor responses obtained in the setting provided by Cattaneo et al. in patients administered with propofol regimen depend on mono- and oligo-synaptic connections only, with no clue on the actual effect of multi-synaptic connections on the functioning of these regions, which are suppressed by anesthesia.

Although our elaborated review focused on complex hand motor behaviors, it is understood that encoding and performance of object-oriented dexterous tasks come together with additional high hierarchical cognitive outputs (the definition of a goal, the social context surrounding the hand motor act being computed, the elaboration of the environmental effect of such action among all). During any identifiable task, a spatiotemporal integration of extensive but significantly specialized networks occurs through a dynamic interaction responsible for the continuous redefinition of equilibrium states and the definition of complex behavioral responses. The theory behind these assumptions, the so-called “meta-networking theory,” would represent one of the reasons for the interindividual behavioral variability we can assess in humans, but also explain the attitude to acquire complex abilities compared to non-human networks and neuroplastic phenomena after injury (Herbet and Duffau, 2020). Compared to previous assumptions related to object-oriented hand dexterous skills, a direct translation of evidence from non-human primate models would be of limited use. Identifying appropriate protocols to address such complexity intraoperatively is still under investigation and is beyond the aim of the current study.

The real-time neuropsychological testing (RTNT) has been proposed as a complementary tool to DES during surgical resection to continuously monitor the patient’s overall neuropsychological status throughout the resection phase. RTNT consists of sequential runs of awake neuropsychological assessments performed during surgery: Tomasino et al. proposed a tailored RTNT protocol for resection in the perirolandic and posterior parietal cortex within the frontoparietal praxis network including the Handedness decision task (HDT), the Florida Praxis Imagery Questionnaire (FPIQ), the Action verb naming (AVN) task, the Kissing and Dancing test (KDT) and the Buccofacial praxis and ideomotor praxis (BP/IMP) tests (additional information is available in Tomasino et al. (2022). This approach’s primary goal is to compensate for the risk of so-called negative mapping during DES (previously discussed); the absence of DES-induced transitory perturbance, in fact, might not accurately predict the “non-functionality” of a specific area. RTNT identified a significant decrease in performance during mental rotation of body parts (HDT) and action imagery (FPIQ) testing: for the latter, resection adjacent to the right postcentral gyrus, SMA, SPL and IPL were accounted, while decreased performance in HDT was more likely experienced during resection of the right cingulum, SMA left SPL, left IPL and medial precuneus. No alterations in the remaining cognitive domains were identified during RTNT in areas within the frontoparietal praxis network, suggesting the presence of a wider compensatory distribution. Compared to previous studies investigating the exact cognitive domains with no RTNT, Tomasino et al. reported no long-term motor cognitive deficits, suggesting RTNT allows online monitoring of sensory-motor cognition in favor of a weighted oncofunctional balance (i.e., mean EOR was 91 ± 17%).

#### 4.3.1 Limitations

In this study, we reviewed decades of pioneering investigations through a systematized selection of imaging-based studies, which we believe are more intelligible to a clinical reader—whom we hereby addressed—compared to invasive studies. However, our systematic review has several limitations to be discussed. First, we focused on the evidence provided by fMRI and PET investigations to characterize the extended grasping network in primates and humans. However, a larger body of literature involving invasive and noninvasive stimulation experiments has been discarded during our systematized search according to the selected query design. Second, a more comprehensive narrative approach was preferred in reviewing all these details in the discussion section, including referenced studies beyond those set during our PRISMA literature search to make our review as exhaustive as possible. However, we cannot guarantee that all highly relevant notions about this extended network’s cortical and subcortical structures have been included in the current manuscript. Third, we grouped all non-human primate species to draw conclusions from animal studies: we are aware of peculiarities differentiating monkeys and apes regarding brain structure and functioning. However, given the limited number of animals selected by our query and the purposes of the qualitative narration, a critical discussion of differences between them was deemed unnecessary.

From a methodological point of view, the heterogeneity of the studies discouraged any quantitative analysis: several authors investigated healthy primates and humans with a variety of behavioral contrasts and both uni- and multivariable statistical models, undermining any general comparison among them all and preventing a meta-analysis from being computed. Finally, the intraoperative mapping protocols present their peculiarities, restraining methodological comparisons to be formulated. Further studies are necessary to standardize the intraoperative brain mapping of complex motor behaviors.

## 5 Conclusion

We provided an updated overview of the current understanding of the extended frontoparietal object-oriented hand manipulation and complex motor behavior network, with a specific focus on the comparative functioning in non-human primates, healthy humans and how the latter knowledge has been implemented in the neurosurgical operating room during brain tumor resection. The anatomical and functional correlates we reviewed highlighted some consistencies, among several relevant differences, in the evolutionary continuum from monkeys to humans, paving the way for a cautious but practical implementation of such evidence in intraoperative brain mapping investigations. Integrating the previous results in the surgical practice might help preserve complex motor abilities, prevent long-term disability and poor quality of life and allow the maximal safe resection of intrinsic brain tumors.

## Data availability statement

The original contributions presented in the study are included in the article/supplementary material, further inquiries can be directed to the corresponding author.

## Author contributions

LT: Conceptualization, Data curation, Formal analysis, Investigation, Methodology, Visualization, Writing – original draft. LM: Data curation, Methodology, Writing – original draft. LV: Conceptualization, Investigation, Methodology, Supervision, Validation, Writing – review & editing. MGal: Data curation, Writing – review & editing. MGam: Writing – review & editing. TS: Writing – review & editing. LG: Writing – review & editing. MC: Writing – review & editing. AG: Writing – review & editing. GC: Supervision, Writing – review & editing, Resources. LB: Resources, Supervision, Writing – review & editing. MR: Resources, Supervision, Writing – review & editing, Conceptualization, Methodology.

## Footnotes

^1^ https://nitrc.org/projects/inia19/
