# Supplementary material for: Object-oriented hand dexterity and grasping abilities, from the animal quarters to the neurosurgical OR: a systematic review of the underlying neural correlates in non-human, human primate and recent findings in awake brain surgery
Source: Front Integr Neurosci. 2024 Feb 15;18:1324581. doi: 10.3389/fnint.2024.1324581 (PMC10902498; doi:10.3389/fnint.2024.1324581)
Supplement: Supplementary file 1 [file Table_1.docx]

Supplementary Table 1: **Main findings, subgroup “Visually-aided grasping” (human primates):** *Abbreviations are reported in the main table.

| **Reference** | **Imaging technique** | **Sample (N)** | **Age (years)** | **Gender** | **Handedness** | **Target** | **Contrast (i.e. grasp > rest)** | **Category** | **Grasp type** | **Details** | **Cortical areas involved** | **Principle findings** | **Abbreviations** |
| --- | --- | --- | --- | --- | --- | --- | --- | --- | --- | --- | --- | --- | --- |
| Begliomini et al (2014) | 1.5 T fMRI | 18 healthy humans | 20–31 years | M:F= 6:12 | Right | Whole brain | Reach-and-Grasp (GL) > Reach (RL) (large stimulus); Reach-and Grasp (GS) > Reach (RS) (small stimulus); Reach (RL) > Grasp (GL) (large stimulus); Reach (RS) > Grasp (GS) (small stimulus) | Grasp (Visually-guided) | precision grip (thumb and index); whole hand grip | Participants were requested to perform two different kinds of movement:  -reach toward and grasp the stimulus;  -reach the stimulus with the hand in a fist posture.  toward either a small or a large stimulus (respectively with a precision and a whole-hand grip) . A Finite Impulse Response (FIR) model was adopted to monitor activation patterns from stimulus onset for a time window of 10 s duration.  Therefore the design (factorial 2 × 2) included four experimental conditions:  -reach to grasp toward a small stimulus (GS); -reach to grasp toward a large stimulus (GL); -reaching only toward a small stimulus (RS);  -reaching only toward a large stimulus (RL). | **GL > GS:**  left aCC, bilateral PreCG (BA 4);   **GS > GL:**  contralateral IPL and aIPS, left aIPS;   **GS *>* RS:** IPL bilaterally (BA 40), right aIPS and pIPS, left aIPS, bilateral aCC, bilateral MFG;   **GL > RL:**  contralateral PreCG (BA 4) and MFG;   **RL > RS:**  contralateral aIPS and pIPS; **RL > GL:** left aIPS, pIPS, right IPS, left mCC | **AIP seems to code for: - type of movement at the planning rather than execution processes; -stimulus dimension at the execution rather than the planning phase.  aCC seems to be engaged in the processing of “accuracy” at the very early stages of action planning; mCC seems to be more devoted at a later stage to spatial coding and the matching between effector and target.  While switching from planning to execution, the left MFG is significantly more alerted for the GL condition.** | aCC: anterio Cingulate Cortex; mCC: middle Cingulate Cortex; IPS: IntraParietal Sulcus; MFG: Middle Frontal Gyrus; PreCG: Pre Central Gyrus; |
| Begliomini et al (2007) | 3 T fMRI | 19 healthy humans | 19-30 years | M:F=7:12 | Right | Whole brain | PG > WHG;  natural > costrained grasp | Grasp (Visually-guided) | precision grip; whole hand grip | Six experimental conditions:  1) Natural reach-and-grasp a small stimulus with PG (PGS) ;  2) Constrained reach-and-grasp a small stimulus with WHG (WHGS);  3) Constrained reach-and-grasp a large stimulus with PG (PGL);  4) Natural reach-and-grasp a large stimulus with WHG (WHGL) ;  5) Reach toward a small stimulus (RS);  6) Reach toward a large stimulus (RL).   Subjects were requested to perform three different actions towards either the small or the large stimulus:  (i) grasping the stimulus independently from its size with a PG;  (ii) grasping the stimulus independently from its size with a WHG;  (iii) reaching the stimulus and touch it with the knuckles of the hand, maintaining it in a closed fist (the fist posture was similar for both small and large objects). | **PG > WHG:**   left AIP;   **natural > costrained grasp**:   dPMC bilaterally and left M1 | **Hand configuration in space: left AIP.  Goal-related hand movements and motor error control: bilateral dPMC (F2vr) .  Motor unit recruitment: left M1** | AIP: anterior intraParietal cortex; dPMC: dorsal PreMotor Cortex; M1: primary Motor cortex. |
| Begliomini et al (2008) | 3 T fMRI | 19 healthy right-handed humans; 15 healthy left-handed humans | RH: mean 24,7 years;  LH: mean 26,1years;  ( 21–35) | Righ handed M:F=7:12;  Left-handed M:F=5:10; | Right and Left | Whole brain | Handness:  (right-handers/ RH+right-handers/LH) > (left-handers/RH+left-handers/LH);  Performing hand (RH):  (right-handers/RH+left-handers/RH) > (right-handers/LH+lefthanders/ LH);  Performing hand (LH):  (right-handers/LH+left-handers/LH) > (Right-handers/RH+Left-handers/RH);  Interaction of handness by performing hand: (righthanders/ RH-right-handers/LH)- (left-handers/RH-left-handers/LH). | Grasp (Visually-guided) | precision grip; | Participants were requested to perform two types of action: grasping the object with a precision grip through the opposition of thumb and index finger (G), or to simply reach it (R), touching it with the back of the hand by using a closed fist posture. Participants were requested to perform the movements with either the right (RH) or the left (LH) hand. The stimulus consisted of a spherical plastic objects of 3 cm diameter presented at a constant distance of 30 cm. Stimulus dimension was chosen in order to elicit a PG type of prehension which considers the opposition of the pulpar surface of the index finger with the thumb. | **Handness: (right-handers/ RH+right-handers/LH) > (left-handers/RH+left-handers/LH):**  M1 bilaterally.   **Performing hand (RH): (right-handers/RH+left-handers/RH) > (right-handers/LH+lefthanders/ LH):** left M1.   **Performing hand (LH): (right-handers/LH+left-handers/LH > (Right-handers/RH+Left-handers/RH):**  bilateral M1 and right dPMC.   **Interaction of handness by performing hand: (righthanders/ RH-right-handers/LH)- (left-handers/RH-left-handers/LH) :** right dPMC (greater for left- than right-handers when the left hand was used to perform the task); cerebellum (6th lobule); bilateralAIP (AIP and cerebellum were significantly more activated in right-handers in respect to left-handers when the performing hand was the right). | **right dPMC monitors the configuration of fingers when precise prehensile movements are performed by either the right and the left hand an this role becomes particularly evident when the hand less-skilled to perform such action is utilized.  Both right- and left-handers prefer the right hand when precise grasp has to be performed.  Eidence in humans of the connections between the cerebellum and AIP.** | AIP: anterior bank and borders of the intraparietal sulcus; dPMC: dorsal PreMotor Cortex; M1: Primary Motor Cortex; . |
| Begliomini et al (2007) | 3 T fMRI | 12 healthy humans | 19–30 years | M:F=4:8 | Righ | Whole brain | GS > RS;  GL > RL;  PG > WHG | Grasp (Visually-guided) | precision grip; whole hand grip | Action toward the object (reaching or grasping) and object dimension (small or large) were manipulated as within-subjects variables, creating four different conditions: grasping small (GS), grasping large (GL), reaching small (RS) and reaching large (RL). The stimuli consisted of two spherical plastic objects (large object, 6 cm diameter; small object, 3 cm diameter). The large object would normally be grasped with a WHG (between the thumb, the surface of the palm and all other fingers). The small object would normally be grasped with a PG (between the index finger and thumb). | **GS > RS:**  left PostCG ; left PreCG; left aIPS; right cerebellum (culmen).   **GL > RL:**  left PostCG and PreCG (both more dorsally than with PG); right cerebellum (culmen).   **PG > WHG:**  left aIPS. | **aIPS is involved in the execution of grasping, however its involvement varies depending on the type of grasp performed: we observed a reliable aIPS activation only during the performance of PG tasks, whereas activation for WHG tasks was absent at the adopted significance threshold.** | aIPS: anterior intraparietal sulcus; PreCG: Pre Central Gyrus; PostCG: PostCentral Gyrus; |
| Begliomini et al (2015) | 1.5 T fMRI | 18 healthy humans | mean 24.7 years (19–30) | M:F=7:11 | Right and Left (Right-handed) | Whole brain | precision grip with RDH + precision grip with LNH > rest | Grasp (Visually-guided) | precision grip | The adopted stimulus consisted of a spherical plastic objects of 3 cm diameter presented at a constant distance of 30 cm.  Participants were requested to grasp the object with either the Right Dominant Hand (RDH) or the Left Nondominant Hand (LNH) using a precision grip.  For each participant eight different models, considering eight different connectivity hypothesis were tested with anatomical models consisting of volumes of interest (VOIs) with reciprocal connections between them (DCM-A matrix) | Grasping with both hands significantly modulated the selected input regions (AIP).  Left hemisphere: The connections AIP-vPMC and vPMC-dPMC appeared as significantly modulated.  The connections AIP-vPMC as well as vPMC-dPMC are significantly modulated, similarly to the left hemisphere.  Connection between AIPs appears to be significantly modulated in the L → R direction but not viceversa.  Concerning dPMC, the connection appears to be modulated in both directions. | **AIP: left to right inter-hemispheric modulation; dPMC: bilateral cross-talking inter-hemispheric modulation** | AIP: anterior bank and borders of the intraparietal sulcus; dPMC: dorsal PreMotor Cortex. |
| Bello et al (2014) | 3 T fMRI | 31 healthy humans | average 26.1years (SD=4.5). | M;F=14:17 | Right | Whole brain | HD execution > control; LD execution > control;  HD execution > LD execution; LD execution > HD execution;   HD observation > control; LD observation > control;  HD observation > LD observation | thumb index opposition (Not specified, more likely no vision) | precision grip | Participants were classified into two groups: ‘‘High dexterity’’ (HD) and ‘‘Low dexterity’’ (LD).  Conditions: -during the execution run, participants were asked to perform the index to thumb opposition task with the right hand; -during the observation run, they watched videos with the aforementioned movement; -during the control condition, they watched static photographs of the same hand with the same perspectives and open and with the fingers in a neutral position.  A multiple regression analysis was performed to look for a linear relationship between brain activity during execution or observation of finger–thumb opposition task and the degree of finger dexterity.  A regression analysis was also performed for selected volumes of interest (VOIs). | **HD execution > control**: right cerebellum; leftITG; left PostCG; left STG; right IFG; left MedFG; right SFG  **LD execution > control**; left PostCG; right cerebellum; right insula; left insula; left PreCG **HD execution > LD execution**; left IFG; right IFG;left MFG; right ITG;   **LD execution > HD execution**: right insula; right PreCU **HD observation > control**; left MOG; left STG; left MTG; right MTG; right ITG; right IPL; right PostCG;  **LD observation > control**; right MOG; right IOG; left ITG; left MOG; left STG; left insula; **HD observation > LD observation:** right STG; right MTG; right ITG; left cerebellum; right PreCG; right MTG | **Although activity in several regions belonging to the MNS showed an increase during both execution and observation conditions, the conjunction analysis revealed that only the left IPL presented shared voxels of activity for both conditions. Therefore, the main activity of the MNS during the execution and the observation of a precision grasping pantomime seem to be located in the IPL and not in premotor areas.  Unlike familiarity, the degree of motor dexterity only seems to modulate brain activation in the MNS during action execution and not during action observation, so motor dexterity may not be a modulator of the MNS.** | IFG: Inferior Frontal Gyrus; IPL: Inferior Parietal Lobule; IFG: Inferior Frontal Gyrus; ITG: Inferior Temporal Gyrus; IPL: Inferior Parietal Lobule; IOG: Inferior Occipital Gyrus; MedFG: Medial Frontal Gyrus MOG: Middle Occipital Gyrus; MTG: Middle Temporal Gyrus; PreCG: PreCentral Gyrus; PostCG: PostCentral Gyrus; PreCU: PreCuneus; SFG: Superior Frontal Gyrus; STG: Superior Temporal Gyrus; |
| Bencivenga et al (2021) | 3 T fMRI | 25 healthy humans | mean age 26.5, s.d. 3.4 | M:F=3:22 | Right | Whole brain | Imagined Grasping > fixation;  Pantomime Grasping > fixation;  Pantomime Grasping > Imagined Grasping | Grasp (Not specified) | finger grip; whole hand grip | Participants underwent an fMRI exam during which they were asked to execute a pantomimed grasping (real condition) or imagine the same movement (imagined condition): -in the “real ”condition, participants were instructed to move the fingers and the wrist of their right hand simulating the grasping of the object, as if it was located in the proximity of their hand.  -in the “imagined ”condition, participants had to imagine and plan the same pattern of movements without actually performing them. In both subjects saw the picture of an object, randomly chosen from a set of 36 black-and-white photographs of commonly used objects. | **Imagined Grasping > fixation:**  PMd; PMv; SMA; pIPS; SPL; IPL; sMG; aIPS (only result on left hemisphere reported)  **Pantomimed Grasping > fixation**:  PMd; PMv; SMA; pIPS; SPL; IPL; sMG; aIPS; M1; S1 (only results on left hemisphere reported)  **DCM analysis matrix A (baseline):** M1 and SMA had a reciprocal negative connection; SMA had excitatory connection to PMv;  PMd had excitatory connections on M1 and SMA, inhibitory on PMv;  PMv had excitatory connection on M1,inhibitory on aIPs and PMd.  **DCM analysis matrix B (modulatory)**: -In the real condition, the major positive modulatory effect of executed grasping propagated from aIPs to PMv, followed by the strong inhibitory feedback exerted by PMv to aIPs.  PMv exerted a positive influence on M1, PMd and, to a lesser extent, on SMA. In turn, only PMd had a negative influence on PMv. -In the imagined condition, a positive influence from aIPs to PMv and from SMA to PMd emerged; moreover, there was an inhibition of PMv exerted by PMd.  **Pantomimed > Imagined Grasping**: aIPs had excitatory connection to PMv; PMd had an inhibitory connection to PMv; | **During real grasping all the forward connections among traditional grasping-related areas (aIPs, PMv, PMd, M1) are excitatory on the contrary, all the feedback couplings are inhibitory. Once the network is activated through aIPs the visuo-spatial representation of the object is conveyed to PMv to select the motor program (e.g., hand posture) appropriate to the object and to encode the timing of the intrinsic hand muscle recruitment.  As the next step of the information flow PMd and SMA both receive an excitatory influence by PMv (both may have a key role in integrating differ ent aspects of the grasping movement, processing low-level features of the movement at high-level processing stages) Since we did not find a direct connection of SMA and PMd to M1 it is still unclear how these areas update the motor execution encoding for low-level motor features. Moreover, our results show that, differently from PMd, SMA does not exert a negative feedback on PMv.** | aIPs: anterior IntraParietal Sulcus; IPL: Inferior Parietal Lobule; M1: Primary Motor cortex; pIPS: posterior IntraParietal Sulcus; PMv: PreMotor ventral; PMd: PreMotor dorsal; S1: Primary Somatosensory cortex;  SMA: Supplementary Motor Area; SmG: supraMarginal Gyrus SPL: Superior Parietal Lobule; |
| Cabinio et al (2010) | 3 T fMRI | 20 right-handed and  20 left-handed healthy humans | RH: mean 28.3 years (20–47) LH mean 29 years (19–53) | RH M:F= 8:12  LH M:F=12 | Dominant hand (both right and left-handed) | Whole brain | RH observe > rest AND RH move >rest;  LH observe > rest AND LH move >rest; | Grasp (Not specified) | whole-hand grip | Every subject underwent 4 fMRI runs: in two runs, subjects were asked to observe and in the remaining two to execute grasping movements.  -In the observation runs, all subjects observed movies of a right (condition A) or a left hand (condition B) grasping different objects.  -In the motor runs, subjects viewed pictures of objects oriented in order to be grasped with the right or the left hand and were verbally instructed to use the corresponding hand; they were asked to execute the grasping movement appropriate to the shape of the object either with their right (condition A) or left hand (condition B). In addition subjects were instructed to execute the movement as if the object would be close to their hand (avoiding reaching).  In both observational and motor runs, during rest periods subjects observed a picture of a still hand (right or left, condition C). | **RH observe > rest AND RH move >rest**: PreCG; BA6; STG; BA40; BA9; PostCG; BA5; BA7; BA37 [the Conjunction-Right analysis -i.e. dominant hand- revealed an activation of premotor BA6, parietal BA5–BA7 almost exclusively in the left hemisphere, together with a bilateral but significantly left-lateralized pattern involving the other frontal (BA9), parietal (BA2, BA40) and extrastriate BA37 areas.  The Conjunction-Left analysis (nondominant hand) showed a similar though more bilateral pattern, with the additional activation of left inferior frontal (BA44), revealed exclusively in this condition. Remarkably, temporal areas (BA20–22–42) were activated exclusively in the right hemisphere, in both Conjunction-Right and Conjunction-Left).  **LH observe > rest AND LH move >rest**: PreCG; BA6; STG; BA40; BA9; PostCG; BA5; BA7; BA37 (the Conjunction-Left analysis -i.e. dominant hand- revealed a bilateral activation, with only a slight right dominance in the activation of the prefrontal cortex (BA6, BA9). The conjunction-Right analysis showed a very similar – with the exception of the left BA44 – but more bilateral pattern of activation. Differently from RH subjects, in LH subjects the temporal extrastriate BA37 revealed a bilateral pattern of activation in both conjunctions. Similarly to RH subjects, in both conditions (Conjunction-Left and-Right) the activation of the temporal areas (BA22–42) in LH subjects was lateralized on the right).   **Lateralization index**: confirmed the results of the fMRI analysis: -RH subjects were always more left lateralized (strictly lateralized for Conjunction-Right, a little less in Conjunction-Left) than LH subjects, while  -LH subjects were almost perfectly bilateral in the Conjunction-Right (frontal and parietal lobes) and slightly right lateralized in the Conjunction-Left conditions. | **The activation of the premotor (BA6) and the parietal (BA40) cortex is strongly lateralized to the left hemisphere in right-handed subjects when observing or moving the right hand, and also, but to a lesser degree, when observing or moving the left hand.  In left-handed subjects, observing and moving either the left or the right hand, the same brain areas were activated, but more bilaterally distributed.  In addition to the classical MNS areas, other areas were identified by the conjunction analysis in both right- and left-handed subjects: the primary somatosensory cortex (BA2 and 3), as well as frontal (BA9), posterior parietal (BA5 and BA7) and occipito-temporal (BA37) areas.** | BA: Broadmann area; MNS: Mirror Neurons System; PreCG: PreCentral Gyrus; PostCG: PostCentral Gyrus; STG: Superior Temporal Gyrus; |
| Cavina-Pratesi et al (2007) | 4 T fMRI | 10 healthy humans | 22–33 years | M:F=4:6 | Right | Whole brain | Size discrimination > baseline; Pattern discrimination > baseline; Reach > baseline; Grasp > Baseline; Grasp > Reach;  Size > Pattern discrimination; | Reach; Grasp (Visually-guided); | precision grip | A set of geometrical nonfunctional plastic rectangles variable in lenght were used as stimuli; the face of each object was covered by a pattern.   In experiment 1, two stimuli were simultaneously presented and partecipants were asket to: -grasp (G) one of the two objects;  -reach toward (R) one of the two objects;  -discriminate between the sizes of the two objects; -discriminate between the patterns of the two objects.  In experiment 2, only one stimulus was presented and partecipants were asked to: -Grasp (G); -Reach (R); or -Passively View one of the presented objects (V). | **Size discrimination > baseline, Pattern discrimination > baseline, Reach > baseline, Grasp > Baseline:** bilateral AIP; (left M1 and left S1 in reach and grasp only); left PostCS; left hIPS; left PO cortex and early visual cortices (V) **Grasp > Reach:**  bilateral AIP; left M1; left S1; left PostCS; left hIPS; left PO cortex and early visual cortices (V).    **Size > pattern discrimination:**  activation in left LO, pIPS, right SMA and the right IPL. | **Although visually-guided grasping of 3D objects requires processing of object size, that computation appears to rely on different neural mechanisms than those involved in the perceptual discrimination of size. AIP showed a response during both size and pattern discrimination, consistent with its activation during passive viewing, but there was no differential response between these two conditions.  Conversely, left LO showed a response during both grasping and reaching, but no difference in the magnitude of this response, consistent with its activation to the visual presentation of any object.  Taken together these results support a dual representation of objects for the purposes of action and perception in neurologically intact human subjects.** | AIP: anterior bank and borders of the intraparietal sulcus; hIPS: Horizontal segment of the IntraParietal Sulcus; IPL: Inferior Parietal Lobule; M1: Primary Motor Cortex; PO: Parieto-Occipital cortex; PostCS: PostCentral Sulcus; S1: Primary Somatosensory cortex; SMA: Supplementary Motor Area; sPCS: Superior PostCentral Sulcus; |
|  |  |  |  |  |  |  | View > baseline; Grasp > Reach |  |  |  | **View > baseline:** bilateral AIP; left PostCS; left hIPS; left PO cortex and early visual cortices (V)  **Grasp > Reach**: bilateral AIP; left M1; left S1; left PostCS; left hIPS; left PO cortex and early visual cortices (V). |  |  |
| Chapman et al (2002) | 1.5 T fMRI | 9 healthy humans | age 18-38 years | M:F=5:4 | Right | Whole brain | Reach-and-Grasp (one to three stimuli) > Reach-and-Grasp (one to three stimuli) | Grasp (Visually-guided) | precision grip | The subjects were told prior to the start of the practice session that any combination of one, two or three stimuli would emerge from the display.  There were five conditions:  (i) three stimuli condition;  (ii) two stimuli condition;  (iii) one stimulus, three possible locations condition;  (iv) one stimulus one location condition;  (v) view condition.   Subjects were instructed to reach-to-grasp (but not hold) the designated target stimulus using only their right-hand thumb and index finger | **Reach-and-Grasp (three stimuli) > Reach-and-Grasp (one stimulus one location):**  left PO sulcus; left SPL; right IPS; right pre-motor and inferior occipital cortical areas.   **Reach-and-Grasp (three stimuli) > Reach-and-Grasp (one stimulus three locations):** left PO sulcus; right IPS;   **Reach-and-Grasp (one stimulus three locations) > Reach-and-Grasp (one stimulus one location):** left SPL; right IPS | **visual selection and action planning mechanisms are processes distributed and modulated within and over different parietal areas spanning from the SPL to the adjacent parts of the IPs.** | IPS: INtraParietal sulcus; PO sulcus: Parieto-Occipital sulcus SPL: Superior Parietal Lobule. |
| Chapman et al (2007) | 1.5 T fMRI | 15 healthy humans | aged 18–38 years | M:F=8:7 | Right | Whole Brain | Reach-and-Grasp (target and non-target stimuli) > Reach-and-Grasp (target stimuli) | Grasp (Visually-guided) | precision grip | Patients had to completa a reach-to-grasp actions towards a designated target stimulus using only the right-hand thumb and index finger. In the present study we compared brain activity during reach-to-grasp movements to a single target with brain activity during reach-to-grasp movements toward a target presented amongst two other objects (a visual cue signalled to the participant the to-be-grasped object). | **Reach-and-Grasp (target and non-target stimuli) > Reach-and-Grasp (target stimuli):** left M1; left Pcu; right mIPS; | **there is specialised neural processing for selecting targets amongst non-target objects** | M1: Primary Motor cortex; Pcu: PreCuneus; mIPS: middle IntraParietal sulcus. |
| Culham et al (2003) | 4 T fMRI | 7 healthy humans | 22-33 years | M:F=4:3 | Right | Whole brain | Grasp > Reach;  Object perception > Image perception | Grasp (Visually-guided) | precision grip | We used functional magnetic resonance imaging (fMRI) to determine whether grasping (compared to reaching) produced activation in dorsal areas, ventral areas, or both. To eliminate activation due to visual and motion stimulation, the subject remained in the dark except for a brief illumination of the target. Just prior to a trial, the cylinder would rotate to place the next object facing the subject. In the grasping condition (G), subjects grasped the long axis of the rectangular target object using a precision grip with the index finger and thumb. In the reaching condition (R), subjects transported the arm to the target location, but rather than forming a grip, they touched the object with the knuckles. | **Grasp > Reach:**  left PostCS; left AIP; right AIP; left thalamus; right thalamus; left FEF; right FEF; left M1; left PO cortex; left posterior cingulate; Visual areas   **Object perception > Image perception:**  left LOC; right LOC; left STG; left posterior Sylvian; right MTG; | **although object grasping and object perception both involve processing of object properties such as shape, size and orientation, they rely on different underlying neural substrates: -Parietal area AIP is activated more strongly by grasping, when object information is required to preshape the hand, but does not respond to images of objects in the absence of an action.  -Temporal area LOC is activated more strongly by objects than scrambled control images, but shows no enhanced activity when real objects are the targets for grasping compared to reaching.** | AIP: anterior IntraParietal area FEF: Frontal Eye Fields; LOC: lateral Occipital cortex; M1: Primary Motor cortex; PO cortex: Parieto-Occipital cortex; |
| Di Bono et al (2017) | 1.5 T fMRI | 23 healthy humans | mean 24.8 ± 4.6years (20-42) | M:F=7:16 | Right | Whole brain | Reach-and-Grasp (individual) vs Reach-and-Grasp (social interaction) | Grasp (Visually-guided) | N/A | Participants were asked to reach toward, grasp an object and put it in a concave base (i.e., individual condition), or pass it to a co-agent (i.e., social condition).  Thee action was divided into a reach-to-grasp and a place phase. The social aspect of the action was manipulated by considering two experimental conditions:  -(i) Individual, in which eeach participant was asked to reach towards, grasp the stimulus (reach-to-grasp phase), and put it in a concave base (place phase);  -(ii) Social, in which each participant was asked to reach towards, grasp the stimulus and pass it to another agent whose hand was placed on top of the concave base (place phase), so that the target location was identical to that of the individual condition in both phases. | **Reach-and-Grasp (individual) vs** **Reach-and-Grasp (social interaction):** Classifier results showed that it was possible to decode the social intention of the action from all the selected ROIs (bilateral IFG, IPL, mPFC and MTG) but not the control ROI (bilateral PCC). Correlation results indicate that the mPFC and the IFG do interact. The decoding accuracy from the mPFC (MS) was positively correlated to that from the IFG (pMNS): the more informative was the activity pattern encoding action intention within the mPFC, the more informative was that within the IFG. | **Areas belonging to the pMNS and the MS are involved in encoding different representational codes for the social and the individual intention of the action: mPCF could interact with the IFG, through a translation of the simulated action code provided by the IFG (i.e., the “what" and the “how” of the action) into an action code equipped with a social meaning (i.e., the social “why” of the action) that could eventually be translated into an appropriate motor command.** | IFG: Inferio Frontal Gyrus; IPL: Inferio Parietal Lobule; mPFC: medial PreFrontal cortex; MTG: Middle Temporal Gyrus; MS: Mentalizing System; pMNS: putative Mirror Neuron System; PCC: Posterior Cingulate cortex; |
| Errante et al (2021) | 3 T fMRI | 16 healthy humans | mean 24.6 years (18–27) | M:F=7:9 | Right | Whole brain | Grasp (Hook open) > Rest;  Grasp (Hook close) > Rest;  Grasp (Precision open) > Rest;  Grasp (Precision close) > Rest;   Observation (Grasp - Hook open) > Rest; Observation (Grasp - Hook close) > Rest; Observation (Grasp - Precision open) > Rest; Observation (Grasp - Precision close) > Rest; | Grasp (Visually-guided) | precision grip; hook grip (i.e with flexed digits 2-5) | Participants performed two tasks:  (a) observation of four different types of actions, consisting in reaching-to-grasp a box handle with two possible *grips* (precision, hook) and two possible *goals* (open, close);  (b) action execution, in which participants performed grasping actions similar to those presented during the observation task.  Thus, the resulted actions included the following conditions:  a) grasping the handle with hook grip to open the box ( *Hook_Open* );  b) grasping the handle with hook grip to close the box ( *Hook_Close* );  c) grasping the handle with precision grip to open the box ( *Precision_Open* );  d) grasping the handle with precision grip to close the box ( *Precision_Close* ). | **Observation (Grasp - Hook open/close + Precision open/close) > Rest:**  pMTG; ITG; PMd; PMv; SPL; IPL; IPS (clusters largely symmetrical, although some of them were more extended in the left hemisphere, such as the IPS and the PMv); right cerebellar lobules VI and VIII (some clusters were present in both hemispheres, although later alized in right one, such as the lobule VIII for Precision_Close actions; clusters peaks were mainly located in right lobule VI).   **Observation (Precision) > Grasp:** bilateral IPL   **Grasp > Rest:**  left M1; left S1; bilateral MCC; bilateral IPL; bilateral IPS; bilateral SPL; bilateral PMd; bilateral PMv; bilateral IFG; bilateral SMA; bilateral putamen and globus pallidus; right cerebellar lobules V-VI, Crus I, and VIII. (activation peaks were at the level of cerebellar vermis and, in the right lateral cerebellar cortex, in lobules VI and VIII.   **Grasp (Precision open) > Grasp (Hook open):** left PMv, left PMd, Left IPL, Left IPS, Left SPL and Right Cerebellar Lobule VI.   **Observation + Grasp > Rest:**   bilateral IPL; bilateral IPS; bilateral SPL; bilateral pMTG; bilateral ITG; bilateral PMd; left PMv; right cerebellar lobules VI and VIII. | **Observation of reaching-grasping actions recruits both dorsal and ventral areas of MNS, irrespective of the final goal of the action or the grip used to perform it.  In addition also cerebellum (lobules VI and VIII) is strongly activated.  Direct contrast between observation conditions did not reveal areas selective for the processing of grip or action goal, nevertheless the ROI analysis showed that:  (a) multiple areas including left PMd, PMv, SPL, IPS, bilateral IPL and right cerebellar lobule VI activate stronger during observation of Precision vs Hook actions;  (b) among the areas revealed only the left IPL shows a modulation of activity for the interaction between grip and action goal.  Interestingly, these results have also been extended using MVPA, that reveals a significant decoding accuracy for grip type, not only in the same areas described in the univariate ROI analysis, but also in additional ROIs, such as Left S1 and Right cerebellar lobule VIII.  The MVPA results also confirm the specific role of left IPL in decoding the final goal of the action, independently from the grip used for its execution.** | IFG: Inferior Frontal Gyrus IPL: Inferior Parietal Lobule; IPS: IntraParietal Sulcus; ITG: Inferior Temporal Gyrus; M1: Primary Motor cortex; MCC: MidCingulate cortex; PMd: PreMotor dorsal; PMv: PreMotor ventral; pMTG: posterior Middle Temporal Gyrus; S1: Primary SomatoSensory cortex; SMA: Supplementary Motor Area; SPL: Superior Parietal Lobule; |
| Fabbri et al (2016) | 3 T fMRI | 12 healthy humans | mean 24 years | M:F=4:8 | Right | Whole brain | Grasp (Precision two digit)> Passive view;  Grasp (Precision five digit)> Passive view;  Grasp (Course palm grasp)> Passive view. | Grasp (Visually-guided) | precision grip; whole hand grip | Participants directly viewed a series of 18 objects, a factorial combination of six shapes and three sizes, and performed one of four tasks: - In the first grasping task, participants used the index finger and thumb to precisely grasp the object (Precision 2 condition).  - In the second grasping task, participants used all five digits to precisely grasp the object (Precision 5 condition).  - In the third grasping task, participants grasped the object coarsely in a whole-hand grasp using all five digits (Coarse 5 condition).  - Passive viewing All grasps were performed with the right hand and in direct view of the stimulus.  To investigate the processing of visual dimensions related to object properties from the motor aspects related to the specific hand configuration, we orthogonally varied object properties (shape, size, and elongation) and task. | **Grasp > Passive View:** M1; S1; PMd; PMv; aIPS; mIPS; pIPS; pMTG; SPOCs; LOC; V1. **Elongation coding:**  V1, aSPOC, pSPOC.   **Digit coding:**  M1, S1, PMv.   **Elongation + digit coding:**  aIPS, pMTG, LOC, pIPS, mIPS, PMd, FEF.   **Object size:** PMd, pMTG.   **Precision:** pMTG. | **We found that object elongation is the most strongly represented object feature during grasping and is coded preferentially in the primary visual cortex as well as the anterior  and posterior superior-parieto-occipital cortex. By contrast, S1, M1 and PMv cortices coded preferentially the number of digits while ventral-stream and dorsal-stream regions coded a mix of visual and motor dimensions. The representation of object features varied with task modality, as object elongation was less relevant during passive viewing than grasping.  To summarize, this study shows that elongation is a particularly relevant property of the object to grasp, which along with the number of digits used, is represented within both ventral-stream and parietal regions, suggesting that communication between the two streams about these specific visual and motor dimensions might be relevant to the execution of efficient grasping actions.** | aIPS: anterior IntraParietal Sulcus; aSPOC: anterior Superio Parieto-Occipital cortex; FEF: Frontal Eye Fields; LOC: lateral Occipital cortex; M1: Primary Motor cortex; mIPS: middle IntraParietal Sulcus; pIPS: posterior IntraParietal Sulcuds; pMTG: posterior Middle Temporal Gyrus; PMd: PreMotor dorsal;  PMv: PreMotor ventral; pSPOC: posterio rSuperio Parieto-Occipital cortex; S1: Primary Somatosensory cortex; V1: Primary Visual cortex; |
| Gallivan et al. (2011) | fMRI 3T | 26 healthy humans | rh mean 28years | rh M:F=6:7; | 13 right | whole brain | [3 × (NRpv) vs. (NLpv + FRpv + FLpv)]  reach > NRpv; reach > NLpv;  reach > FRpv;  reach > FLpv.  grasp > NLpv;  grasp > FRpv;  grasp > FLpv. | grasp visually guided; | precison grip | On the majority of trials, participants were simply required to passively view a single object placed at one of four locations (near right: NRpv, near left: NLpv, far right: FRpv, and far left: FLpv). Object location NL was placed within reach by the participant’s left hand and NR was placed by the experimenter at a symmetric, equally distant location (with respect to the participant) at an unreachable location for the left hand, but typically actable by the right hand; locations FR and FL were placed 20 cm outside of reach of the participant on the right and left respectively, at symmetric, equally eccentric locations as the two near locations.  The fixation pointbehind the far conditions so that all objects were positioned in either the participant’s lowerleft (NLpv, FLpv) or lower-right (NRpv, FRpv) visual field.  On action trials participants were required to perform grasping (using a precision grip with the thumb and index finger) or reaching (manually touch the object with the knuckles, without hand preshaping) to an object located along the arc of reachability with their left hand, at the point corresponding to the participant’s sagittal midline. Target stimuli were of two sizes (large and small) allowing objects at different depth locations to provide the same retinal image size. The six trial types (Grasp, Reach, NRpv, NLpv, FRpv, and FLpv) were pseudorandomly intermixed. | **ROI analysis,  tasks in right handed**  **[3 × (NRpv) vs. (NLpv + FRpv + FLpv)]:** left SPOC;  **reach > NRpv / reach > NLpv / reach > FRpv / reach > FLpv / grasp > NLpv / grasp > FRpv / grasp > FLpv with right hand**: left SPOC  **2 × 2 RFX ANOVA at the group level (object distance (near vs. far) × side of space (left vs. right)) using the four PV**: left SPOC for laterality | **The activation within a brain area implicated in reaching, SPOC, is linked with the hand preferences subjects have for acting within particular regions of space: lefthanders, in contrast to right-handers, use both hands equally when reaching out to grasp objects.  Consistent with this, in SPOC we find that right-handers show enhanced responses to near right locations, the workspace within range of their dominant limb (and where they typically act upon objects); whereas, left-handers show enhanced responses to bilateral locations, the workspace within the range of either their right or left limb.** | SPOC: Superior Parieto-Occipital cortex; |
|  |  |  | lh mean 27years | lh M:F=6:7 | 13 left |  | NRpv > FRpv; NRpv > FLpv; NLpv > FRpv; NLpv > FLpv. [(NRpv + NLpv) > (FRpv + FLpv)];  ([3 × (NLpv) >(NRpv + FRpv + FLpv)] and [3 × (NRpv) > (NLpv + FRpv + FLpv)];  reach > NRpv; reach > NLpv;  reach > FRpv;  reach > FLpv.  grasp > NLpv;  grasp > FRpv;  grasp > FLpv. |  |  |  | **ROI analysis,  tasks in left handed**  **NRpv > FRpv / NRpv > FLpv:** left SPOC  **NLpv > FRpv / NLpv > FLpv**: SPOC  **[(NRpv + NLpv) > (FRpv + FLpv)]**; left SPOC  **([3 × (NLpv) >(NRpv + FRpv + FLpv)] and [3 × (NRpv) > (NLpv + FRpv + FLpv)]**: left and right SPOC  **2 × 2 RFX ANOVA at the group level (object distance (near vs. far) × side of space (left vs. right)) using the four PV**: left SPOC for distance |  |  |
| Grafton et al (1996) | PET, 1.5 T fMRI | 9 healthy humans | mean 24years (19-43) | M:F=7:2 | Right | Whole brain | Grasp > Passive view;  Grasp + Point > Passive view;  Grasp > Point; Passive view > Grasp | Grasp (Visually-guided) | N/A | Partecipants performed three visually guided motor paradigms during PET imaging: grasp, point, and control: -In the grasp task, subjects reached and grasped cylindrical targets repetitively for the duration of the 90 second PET scan; then, they were told to reach and grasp the sides of the illuminated dowel using a precision grasp.  -In the point task, the same target array was used, subjects were instructed to point over the top of the dowels with their right hand using a natural pointing hand position with the index finger extended and all others flexed in a comfortable fist.  -In the control task (passive view), subjects were told to leave the right hand at rest on the chest and to simply look at each target as it was illuminated. | **Grasp > Passive view:** left sensorimotor cortex; left cingulate cortex; right inferior temporal cortex; bilateral anterior cerebellum; **Movement (Grasp or Point) > Passive view;**  Contralateral M1, S1, SPC, SMA, CMA. Bilateral posterior parietal cortex, inferior temporal and occipital cortex (PO and cuneate cortex).   **Grasp > Point:**   left lateral PO (S2).   **Passive view > Grasp**: right PPC | **there is increased activity whether a subject is reaching and pointing toward or reaching and grasping simple cylindrical objects at  -the genu of the central sulcus, i.e arm and hand motor area;  -nearby precentral suicus and near the superior frontal suicus, i.e premotor areas; -in superior parietal cortex, i.e visuomotor control area.** | CMA: Cingulate Motor Areas; M1: Primary Motor cortex; PO: Parietal Operculum; PPC: Posterior Parietal cortex; S1: Primary Somatosensory cortex; S2: Sec ondary Somatosensory cortex; SMA: Supplementary Motor Area; SPC: Superior Parietal cortex; |
| Grèzes et al (2003) | 2 T fMRI | 12 healthy humans | 19–39 years | M:F=12:0 | Right | Whole brain | Observation (all) > Rest;  Observation (gesture) > Observation (object); Observation (object grasped) > Observation (object);  Grasp (object) > Observation (object);  Grasping (pantomime) > Observation (grasping action);  Grasping (pantomime grasping gesture toward object) > Observation (grasping gesture toward object);  Grasping (pantomime) > Grasping (pantomime grasping gesture toward object); | Grasp (Visually-guided) | precision grip; powe grip (i.e between the thumb, the surface of the palm, and all other fingers). | Stimuli consisted of video recordings of objects, grasping pantomimes, and objects being grasped.   In the observation context, subjects - observed an object (OO),  -observed a grasp (OG), or  -observed an object being grasped (OGO);  -in the baseline condition they observed a stationary background (OB).   In the execution conditions, subjects -executed the grasp appropriate for the object that they viewed (EO),  -imitated the pantomime they viewed (EG), or  -imitated the hand grasping an object (EGO);  -in the baseline condition subjects executed the same grasp (power grip) on all trials while viewing a stationary background (EB).   This forms a 2 x 2 x 2 factorial design (execute vs observe, presence vs absence of object, presence vs absence of gesture).   Two different objects were used:  -one had large opposition axes and would normally be grasped with a power grip (between the thumb, the surface of the palm, and all other fingers); -the other object was small and would normally be grasped by a precision grip (between the index finger and the thumb).  The two types of grasps and their associated object size were not distinguished in the analysis. | **OO > OB AND EO > EB**: left PMv  **OG > OB AND EG > EB**: bilateral PMd; left PMv; bilateral IPS; right S2; bilateral STS;   **OGO > OB AND EGO > EB**: bilateral PMd; bilateral IPS; right S2; bilateral STS;  **(EO > EB) - (OO > OB)**: IFG-pT;   **(EG > EB) – (OG > OB)**: IFS;  **(EGO > EB) – (OGO > OB)**: PMv; IFG-pT;  **(EG > EB) – (EGO > EO)**: PMv; IFG-pT; BA44; | **There was activation in the intraparietal and ventral limbs of the precentral sulcus when subjects observed objects and when they executed movements in response to the objects (canonical neurones).   There was activation in the dorsal premotor cortex, the intraparietal cortex, the parietal operculum (S2), and the superior temporal sulcus when subjects observed gestures (mirror neurones).   Finally, activations in the ventral premotor cortex and inferior frontal gyrus (area 44) were found when subjects imitated gestures and executed movements in response to objects.   We suggest that in the human brain, the ventral limb of the precentral sulcus may form part of the area designated F5 in the macaque monkey: it is possible that area 44 forms an anterior part of F5.** | IFG: Inferior Frontal gyrus; IFG-pT: Inferior Frontal gyrus-pars triangularis; IFS: Inferior Frontal Sulcus IPS: IntraParietal Sulcus; PMd: PreMotor dorsal; PMv: PreMotor ventral; S2: Secondary somatosensory cortex; STS: Superior Temporal Sulcus; |
| Gutteling et al (2015) | 7 T fMRI | 6 healthy humans | mean 25.8 years | M:F=2:4 | Right (5 right-handed, 1 left-handed) | Whole brain | **Grasp > Pointing/Rest** | Grasp (Visually-guided) | precision grip | Subjects performed three tasks in two separate fMRI sessions: -a grasping and pointing task to oriented bars (when pointing, they hed to point to the center point of the bar object; when instructed to grasp, thumb and index fingers were to be placed at the short sides of the bar);  -a passive viewing orientation Task.   During the grasping/pointing task subjects performed four grasping and four pointing blocks alternatively, every block started with an instruction of the action to-be performed in the upcoming block (grasping or pointing) indicated by a color cue; after 3.5– 4.5 s (random interval), an auditory cue instructed to perform the action (“go” cue); shortly afterward the movement that was prepared, had to be withheld (“no-go” cue). This resulted in six possible events per trial: -grasping instruction;  -grasping go;  -grasping no-go;  -pointing instruction; -pointing go; and  -pointing no-go.  Subjects also engaged in a standard retinotopic mapping protocol. | **Grasp > pointing**: superior parietal areas, extending into the IPS and in the occipital lobe, along the calcarine sulcus, lateral occipital areas and the parieto-occipital junction.  **go condition**: aIPS; V1; V2; V3.  **no-go condition**:  V1, V2, and V3. | **grasping preparation modulates orientation selectivity in the early visual areas, enabling action-modulated perception effects that improve processing of action relevant visual features.** | aIPS: anterior IntraParietal Sulcus; IPS: IntraParietal Sulcus; V1: Primary Visual cortex; V2: Secondary Visual cortex; V3: Third Visual cortex. |
| Chapman et al (2011) | 3 T fMRI | 15 healthy humans | mean 26.2 years | M:F=8:7 | Right | Whole brain | Planning > Rest (Wrist posture x obstacle position);  Grasp > Planning (Wrist posture x obstacle position); | Grasp (Visually-guided) | precision grip | Participants were required to grasp the target object using one of two wrist postures (thumb-front or thumb-side) and obstacles could be in one of three configurations (not present, to the side or behind the target object), leading to 6 total conditions in a 2 x 3 design.  All grasps used only the index finger and thumb placed on opposing surfaces of the target object.  To isolate areas critical to visuomotor planning, we used a slow event-related paradigm consisting of three distinct phases:  -Preview,  -Plan and  -Execute.  Although participants knew the object to be grasped during the Preview phase, only in the Plan phase did they have all the information necessary to prepare the upcoming movement. | **Plan > Preview:**  a large network extending from early visual areas in the occipital lobe (where activity is bilateral) dorsally along the left IPS and into both left motor and medial frontal areas including premotor cortex.  The left PMC, visual and IPS cortex show a definitive rise and fall of activation across the Plan phase but left M1 remains relatively flat, instead showing a gradual ramping up of activity prior to movement. More importantly, both the left visual and IPS cortex show activity above baseline during the Preview phase.  **Execute > Planning**:  One area in visual cortex (with activation that spread bilaterally, but with the peak voxel and center of mass in the right hemisphere) showed an overlap with that defined during the Preview phase and resulted in larger differences due to Obstacle-Position (side location of the obstacle) at the early Execute time points.  Left aIPS showed a significant activation during the Execute phase only for the thumb-side wrist posture compared to other conditions. | **Within a specified network of areas involved in planning, a group voxelwise analysis revealed that one area in the left pIPS and one in early visual cortex were modulated by the degree of obstacle interference, and that this modulation occurred prior to movement execution. This is the first concrete evidence that the planning of a grasping movement can modulate early visual cortex and provides a unifying framework for understanding the dual role played by the IPS in motor planning and attentional orienting.** | aIPS: anterior IntraParietal sulcus; M1: Primary Motor cortex; PMC: PreMotor cortex; pIPS: posterior IntraParietal Sulcus; |
| Hinkley et al (2009) | 1.5 T fMRI | 14 healthy humans | N/A | N/A | Right | Whole brain | Saccades > fixation Reach-and-Grasp > Rest | Grasp (Visually-guided) | N/A | Subjects participated in three corresponding conditions in a single scan session:  1) visually directed saccadic eye movements towards stimuli appearing at different locations (during control period they maintained fixation while preparing for the target stimuli to appear). 2) visually guided reaching to grasp of target object (tennis ball) suspended above their midline and grasp it with their right hand (during control period they performed a motor-control task in which they held the target object with the right hand, lifted and squeezed it while their eyes were closed). 3) manual shape discrimination in which patients manipulated hand-sized geometric plastic blocks with their right hand only, these blocks were the same weight and texture and differed only in surface configuration (during control period they were presented with similarly sized amounts of clay that had no well-defined structure during motor control blocks and were asked to manipulate this clay with the same hand in a manner similar to that in the experimental block). | **Saccades > fixation**: bilateral rSPL; bilateral lSPL; bilateral junction of the caudal IPS + PO;  **visually guided reaching to grasp > motor control comparison**: left mePoCS; left rostral IPS; left caudal SPL; right medial IPS; right caudal IPS;  **manual shape discrimination > motor control:** bilateral junction of the IPS + PoCS; bilateral medial SPL; | **Multiple regions of the PPC were active during visual-manual behaviors.  Response patterns were complex, with some areas most active in response to a single condition, whereas the majority were active during multiple conditions. The gross areal organization of human PPC is likely similar to the pattern previously described in nonhuman primates, including multifunctional regions and asymmetric processing of some manual abilities.** | IPS: IntraParietal sulcus; MIP: medial intraparietal area; PPC: Posterior Parietal cortex; PO:Parieto-Occipital sulcus; PoCS: PosteCentral Sulcus; SPL: Superior Parietal Lobule; |
| Knights et al (2021) | 3 T fMRI | 19 healthy humans | mean 23 ± 4.2 years (18–34) | M:F=10:9 | Handness N/A / Right hand | Whole brain | Typical > Atypical tool actions; Tool > Non-tool actions; Non-tool > Tool actions; Actions > baseline; small vs large grip size; | Grasp (Visually-guided) | precision grip | Participants were scanned in complete darkness using a head-tilted configuration that allowed direct viewing of the workspace and 3D stimuli.  Tool and nontool object categories were designed [three common kitchen tools (knife, spoon, and pizzacutter) and three nontool control bars].  During the ON-block, the object was briefly illuminated for (0.25 s) five consecutive times (within 2 s intervals) cueing the participant to grasp with a right-handed precision grip along the vertical axis.  Between actions, participants returned their hand to a “home” position with their right hand closed in a fist on their chest. Crucially, since the tool handles were always oriented rightward, the right and left tool trials involved grasping tools either by their handle (typical) or functional end (atypical), respectively. On the other hand, grasping nontools did not involve a typical manipulation but only differed in grasp direction with right versus left grasps | **Typical > Atypical tool actions:** LOTC (hand); IPS (hand); **Tool > Non-tool actions:** PMv (tool); SMG (tool);  **Non-tool > Tool actions**: PMd (tool);  **Actions > rest:** pFs (body); LOTC (body);  **Reapeted measures ANOVA with ROI (hands vs tool selective) and object category (tool vs nontools) as within factors**: for IPS-hand, grasp-type decoding was significantly higher for tools than non-tools, but not for IPS-tool;  **all Grasps > baseline using a left somatosensory cortex (SC) ROI**: grasp-type decoding in SC was significantly greater than chance for both tool and non-tools;  **small vs large grip size**: IPS-hand; EVC; LOTC-object; pFs; SMG; PMv; PMd | **parietal and occipital visual regions specialized for representing hands were found to encode information about the functional relationship between the grasping hand and a tool, implicating hand-selective cortex in motor control.  These findings raise novel questions about the possibility that overlapping hand- and tool-selective regions are functionally distinct and begin to uncover which brain regions evolved to support tool use, a defining feature of our species.** | EVC: Early Visual cortex; IPS: IntraParietal sulcus; LOTC: Lateral Occipito Temporal cortex; pFs: posterior fusiform sulcus; PMd: PreMotor dorsal; PMv: PreMotor ventral; S1: Primary Somatosensory cortex; SC: sensoryMotor cortex; SMG: SupraMarginal Gyrus; |
| Frey et al. (2015) | fMRI 3T | 18 healthy humans | mean 24.4 years (18.7–39.5) | M:F=6:12 | right | whole brain | premovement reach/grasp and reach/grasp>rest; | grasp (visually guided) | N/A | The workspace was placed across the participant’s lap, participants viewed a live video of the workspace. A five-button response pad was positioned at a comfortable distance on the midsagittal plane. At the onset of each trial, a visual instructional word cue (“Reach” or “Grasp”) indicated which movement would be involved, this was followed by a variable delay interval: the **premovement phase** statred with the onset of the instructional cue and included the subsequent delay. Next, an execution phase began with the onset of the live video stream of the workspace which signaled participants to initiate their movements and included the subsequent period: -In the **Reach** condition, the fingertips remained together throughout the movements; on each trial participants released the start button, reached forward and touched the 6-mm radius circle located on the top of the target block with the fingertips, moved the hand laterally to the circular opening, returned to the start position, and depressed the button.  -In the **Grasp** condition, participants released the start button, reached toward and grasped the target object with the fingertips, transported it laterally, and dropped it into the circular opening before returning to the start location and depressing the button. | **premovement grasp/reach>rest**:  occ; SPL; vPMC; dPMC.    **execution grasp/reach>rest**: bil PPC; PMC; cerebellum; BG.   **execution grasp>reach**:  aIPS; lateral postCG; CS; rostral bank of the preCG | **premovement reach/grasp>rest: significant increases within the occipital cortex, extending dorsally into the medial superior parietal lobule, as well as in left vPMC and dorsal premotor cortices. Bilateral activity at the TPJ (ventral attention network). In the left hemisphere, this cluster of increased activity extended into the caudal left middle temporal gyrus (cMTG).   execution reach/grasp>rest: bilateral posterior parietal and premotor cortex and subcortical regions (cerebellum and BG).  execution reach>grasp: contralateral aIPS, this cluster extended rostrally along the lateral convexity of the postcentral gyrus, through the central sulcus, and onto the rostral bank of the precentral gyrus** | aIPS: anterior IntraParietal Sulcus; BG: Basal Ganglia; CS: Central Sulcus. dPMC: dorsal PreMotor cortex; PMC: PreMotor cortex; PPC: posterior Parietal cortex; postCG: postCentral Gyrus; preCG: preCentral Gyrus; SPL: Superior Parietal Lobule; vPMC: ventral PreMotor cortex; |
| Frey et al. (2005) | fMRI 1,5T | 14 healthy humans | 20-25 years | M:F=9:5 | right | whole brain | grasp > point | grasp (visually guided) | precision grip (here referred as pincer grip) | Each epoch consisted of three blocked conditions presented in the following fixed order: point, grasp, and rest. Each block began with the subject lying motionless, eyes closed.  The experimenter tap the subject on the right thigh and subsequently the subject open his eyes and begin the task: -During the point condition, subjects used the right index finger to contact the center of the advanced stimulus object and push it back to the vertical plane. -During the grasp condition, the right thumb and index finger were used to pincer grip the object, remove it from the spindle, and drop it.  -In the rest condition, subjects remained motionless with their eyes closed after receiving the tap. | **grasp > point**:  intersection of IPS with PostCS;a site spanning left Syl fissure and includes parietal operculum (putative S2) | **existence of a functional homologue in the human brain of macaque area AIP.  As in the macaque, aIPS is located near the junction of the anterior IPS and postcentral sulcus; in both species this region appears to play a key role in visuomotor transformations involved in grasping and object manipulation.** | IPS: IntraParietal Sulcus; PostCS:PostCentral Sulcus; S2: Secondary Somatosensory cortex; |
| Gallivan et al. (2009) | fMRI 4T | 10 healty humans | mean 28 years | M:F=4:6 | right | whole brain | passive viewing of reachable>unreachable; grasp / reach > passive view | grasp (visually guided) + lift; grasp (visually guided) with a tool; | precision grip; | Participants were required to perform action trials (grasping and reaching) and passive viewing trials (control) toward target stimuli placed at different spatial locations. An event-related paradigm was used in which each trial lasted consisted of a cue period, a preparation period, an action period and a final intertrial interval. Participants were instructed to always maintain their gaze on a fixation point (situated along the sagittal midline at a visual angle of 16° above the participants’ natural line of gaze). -grasp trials involved using a precision grip to manually pick-up (grasp and lift) the object at the medium location; -reach trials required reaching toward the object at the medium location without pre-shaping the hand and manually touch the stimuli with the knuckles; -passive viewing (“look”) condition involved simply viewing the location of the illuminated object whilemanteining the fixation point.  Participants performed actions with the right hand and had the right upper arm braced. | **passive viewing of reachable > unreachable**:  SPOC (superior end of POS) ; medial OL above CalS;  **grasp / reach > passive view**:  SPOC (superior end of POS); medial OL above CalS; | **SPOC was more active for objects within reach than objects beyond reach even when no action was required.  The focus of activation was highly consistent across experiments despite methodological differences in the locations tested.** | IPL: Inferior Parietal Lobule; OL: Occipital Lobe; POS: Parieto-Occipital Sulcus; PreCU: PreCuneus; SMG; SupraMarginal Gyrus; SPOC: Superior ParietoOccipital cortex |
|  | fMRI 4T | 14 healty humans | mean 26 years | M:F=10:4 | right | whole brain | passive view NR > MR + ML+ MR+ FR; passive view with tool NR > NL + MR + ML+ MR+ FR; |  |  | During odd-numbered runs, which constituted experiment 2a, participants used the hand to reach and grasp objects on some trials while on other trials they simply passively viewed objects at a variety of locations.  -For action trials participants were required to perform the grasping  and reaching to an object located along the arc of reachability, at the point corresponding to the participants sagittal midline.  -During passive viewing conditions, they were instructed to attend to one of five other object locations: two near locations [near right (NRpv) and near left (NLpv) locations] positioned in the right and left visual fields, equally eccentric from fixation (such that only the NRpv location was within range of the participant’s reach); two middle locations on the left and right [medium left (MLpv) and medium right (MRpv) locations], both positioned at a unreachable further distance; and a far location [far right (FRpv) location] that was positioned in the right visual field far beyond reach of the hand.  During even-numbered runs, which constituted experiment 2b, paticipants used a tool to reach and grasp objects in some trials; on othertrials, they simply passively viewed objects at the same locations as experiment 2a while holding the tool in hand.  -On action trials participants performed grasping and reaching with a set of large, plastic tongs to objects at a tool (T) location (along the arc of reachability with the tool, located along the participant’s sagittal midline but further than the location in 2a).  -On passive viewing trials, participants kept the tool in hand at the starting position while objects were presented at one of the samelocations as experiment 2a (NRpv, NLpv, MRpv, MLpv, FRpv), but in this case the range of space reachable with the tool now encompassed passive viewing locations (NRpv, NLpv, and MRpv) | **passive viewing of reachable > unreachable**:  SPOC (superior end of POS); bilateral PreCu;  **passive view NR > MR + ML+ MR+ FR**: bilateral PreCu; much weaker activation of bilateral SMG; right IPL; left OL;  **passive view with tool NR > NL + MR + ML+ MR+ FR**: SPOC (superior end of POS);  **grasp / reach > passive view**: SPOC (superior end of POS); |  |  |
| Glover et al. (2012) | fMRI 3T | 21 healthy humans | N/A | N/A | right | whole brain | P > O; P > C; P + C > C;  C > O; C > P; P + C > P; | grasp (visually guided); | precision grip (thumb and forefinger) | Participants began each trial withtheir eyes closed and were then played each of the tones corresponding to of the four types of trials used: (i) Observation (O), i.e merely observe the target while remaining motionless;  (ii) Planning (P), i.e prepare a right-handed reaching and grasping movement towards the target but remain still;  (iii) Control (C), i.e reach out with the right hand, pick up the target using the thumb and forefinger of their right hand, place it down again, and then return hand to the starting position;  (iv) Planning + Control (P + C), i.e plan thereaching to grasp movement while the corresponding tone played, but then execute it immediately once the tone changed.   During all conditions, the set-up was such that the right hand was visible to the participant as soon as they opened their eyes and remained visible throughout each trial regardless of trial type, whereas the left hand was out of sight at all times. | **P > O: l**eft PMC; bilateral insula; pre-SMA; **P > C:** bilateral insula; pre-SMA; left DLPFC; right STG; left MIP;  **P + C > C:** right STG; left MIP; right PMP; right LOC; **C > O**: bilateral SM1; bilateral SPL; left SMA; left SMG; bilateral OCC; mid Cereb; right Cereb; left thal;  **C > P**: bilateral SM1; bilateral SPL; left SMA; left SMG; bilateral OCC; mid Cerebellum; right Cerebellum; left thalamus;  **P + C > P**: bilateral SM1; bilateral SPL; left SMA; left SMG; bilateral OCC; mid Cerebellum; left thalamus; | **Activity related to pre-movement planning was observed in the STS, pre-SMA, MIP, PMP, SPOC, PMC  and insula.  In contrast, activity related to online control actions was found in an entirely separate, non-overlapping set of regions including the sensorimotor cortex, cerebellum, SMA, SMG and SPL.   These results confirm the existence of separate neural systems for the planning and control of reaching and grasping.** | Cereb: cerebellum; DLPFC: DorsoLateral PreFrontal cortex; MIP: middle IntraParietal sulcus; OCC: Occipital lobe; pre-SMA: pre Supllementary Motor Area; PMC: PreMotor cortex;  PMP: posterior medial Parietal area; SM1: Primary SensoryMotor cortex;SMA: Supplementary Motor area; SMG: SupraMarginal Gyrus; SPL: Superior Parietal Lobule; STG: Superior Temporal Gyrus; |
| Grol et al. (2007) | fMRI 3T | 20 healthy humans | mean 25 ± 4 years | all males | right | whole brain | small > large handle grasp | grasp (visiusally guided) | N/A | During the task, in each trial subjects had to grasp the object at either the large (LARGE) or the small (SMALL) part, remove the object from the rail to put it into one of the two slots, and finally reposition the object in the supporting rail.  When the LED switched on, subjects had to leave the home key as soon as possible, make the appropriate object manipulation (based on LED colour), and return to the home key before the LED switched off. | **SMALL > LARGE**:  forward connection between V3A and V6A, from V3A to AIP, from AIP to PMv | **Within the dorsomedial circuit, grasping movements toward both the SMALL and LARGE objects increased the coupling from V3A to V6A significantly, whereas the connection from V6A to PMd (dorsomedial circuit) was enhanced significantly during LARGE only.  In contrast, within the dorsolateral circuit, the couplings, from V3A to AIP and from AIP to PMv (dorsolateral circuit), were significantly enhanced only when the SMALL object was grasped. These findings suggest that the specification of prehension parameters involves different portions of the parieto-frontal network.** | AIP: anterior IntraParietal area; PMd: PreMotor dorsal; PMv: PreMotor ventral; V3A: Third Visual cortex A; V6A: dorsomedial area A; |
| Hamzei et al. (2016) | fMRI 3T | 102 healthy humans | mean 25.7 years | N/A | right | whole brain | IMI>RE  AO>RE  GR>RE  IMI>AO  IMI>GR  AO>GR  GR>AO | grasp (visually guided and no vision); | Precision grip (here refferred as pinch grip) | The following fMRI conditions were applied in a block design in 1 session:  -REST, volunteers fixated a cross in the middle of the screen; -Action observation (AO), videos of a hand grasping objects were presented with the right hand from the first person position; -Imitation of object grasping (IMI), partecipantss observed a video while a hand grasped an object and were requested to imitate the observed movement with their right hand according to the videos; -Grasping objects (GR), objects were grasped with the right hand while fixating a cross in the middle of the screen. | **IMI>RE**:  vPM; dPM; IFG-pT  **AO>RE**:  IFG-pT; dPM  **GR>RE**: vPM  **IMI>AO**:  vPM; dPM  **IMI>GR**:  vPM; dPM; IFG-pT  **AO>GR**:  dPM; IFG-pT  **GR>AO**:  vpM;  **conjunction IMI and AO**:  dPM; IFG-pT; PPC (SPL; 7PC); AIP; VTG; and the visual cortex | **Action observation with Imitation are mainly a function of the dorsal stream centered on dorsal part of BA44, but also involve BA45, which is dorsally and ventrally connected to the same postrolandic regions.  The current finding suggests that BA45 is the crucial part where the MNS and the dual-loop system interact.** | AIP: anteriorIntraParietal area; IFG-pT: Inferior Frontal Gyrus pars Triangularis; dPM: dorsal PreMotor; PPC: Posterio Parietal cortex; SPL: Superior Parietal Lobule; vPM: ventral Premotor; VTG: ventral temporal gyrus 7PC: area 7 Post-Central; |
| Knights et al. (2022) | fMRI 3T | 19 healthy humans | mean 23 ± 4.2 years (18–34) | M:F=10:9 | N/A | whole brain | [typical (right) versus atypical (left) grasps of tools] - [right versus left grasps of control non-tools] | grasp (visually aiuded) | precision grip | A block began with an auditory instruction (‘Left’ or ‘Right’) and participants grasped the object during the ON-block while it was briefy illuminated using a right-handed precision grip along the vertical axis. Te 3D-printed kitchen tool and biomechanically matched non-tool bar objects were mayched for dimensions, such that variability was minimized and kinematic requirements were as similar as possible between diferent grasps (i.e., lef vs. right and small vs. large). | **[typical (right) versus atypical (left) grasps of tools] - [right versus left grasps of control non-tools]**:  anterior left STG and MTG extending into PHG;  right FG; right aSPOC; | **The anterior temporal cortex processes conceptual knowledge that is feature invariant (i.e., generalises across exemplar identities) like the typical way tools are handled for use (i.e., grasp tool by its handle).** | aSPOC: anterior Superior Parieto-Occipital Cortex. FG: fusyform Gyrus; MTG: anterior Middle Temporal Gyrus; PHG: ParaHippocampal Gyrus; STG: Superior Temporal Gyrus; |
| Kròliczac et al. (2007) | fMRI 4T | 10 healthy humans | mean 27 years (22-32) | M:F=5:5 | right | whole brain | grasp>reach; pantomime grasp>pantomime reach; real>pantomime; grasp/reach/pantomime grasp/pantomime reach/passive view>baseline; | grasp (visually guided) | precision grip | We used a slow event-related design with trials. Each trial began with a brief illumination of the object; before target illumination, a colored fixation point indicated which of the tasks a participant was to perform on the upcoming trial -GRASP, i.e grasping the object with the index finger and thumb along its long axis. -REACH, i.e reaching toward the object and touch it with the knuckles, without preshaping the hand. -PANTOMIME GRASP, i.e the index finger and thumb were to be scaled to the size of the object and the wrist adjusted according to object orientation, but, instead, the fingers touched the surface of the drum in an adjacent location to the right of the target object. -PANTOMIME REACH, i.e adjusting the wrist according to the object orientation, but touching the nearby surface to the right of the object with the knuckles only.  In addition, in the last six of ten participants, a PASSIVE VIEWING condition was added. | **grasp > reach**:  bilateral AIP; left M1; leftS1; left thalamus; right cerebellum; right amIPS, right IFS/PreCS (close to pars opercularis); left STG.  **pantomimed grasping > pantomimed reaching**: right SPL; right amIPS; bilateral SMG, right SMA complex; right TPJ; right lsPO; right pmIPS; right insula; left thalamus; left cerebellum.  **pantomimed>real**:  right MTG/STS; right ITS | **in the anterior intraparietal area, there was significantly higher activation during real grasping than that during real reaching. However, the activation difference between pantomimed grasping and pantomimed reaching did not reach statistical significance.  There was also no effect of pantomimed grasping within the ventral stream, including an object-selective area in the lateral occipital cortex. Instead, we found that pantomimed grasping was mediated by right-hemisphere activation, particularly the right parietal cortex.   These results suggest that areas typically invoked by real actions may not necessarily be driven by “fake” actions. Moreover, pantomimed grasping may not tap object-related areas within the ventral stream, but rather may rely on mechanisms within the right hemisphere that are recruited by artificial and less practiced actions.** | AIP: anterior IntraParietal area; IFS: Inferior Frontal Sulcus; IPS: IntraParietal Sulcus; lsPO: lateral superior Parieto-Occipital sulcus;  M1: Primary Motor cortex; mIPS: middle IntraParietal Sulcus; MTG: Middle Temporal Gyrus; PreCS: PreCentral Sulcus; S1: Primary Somatosensory cortex; SMA: Supplementary Motor Areas; SMG: SupraMarginal Gyrus; SPL: Superior Parietal Lobule; STG: Superior Temporal Gyrus; STS: Superior Temporal Sulcus. TPJ: Temporo-Paietal Junction; |
| Leo et al. (2016) | fMRI 3T | 9 healthy humans | mean 25 ± 3 years | M:F=4:5 | right | whole brain | kinematic synergy > individual digit / muscle synergy models | grasp (visually guided) | N/A | Participants were instructed to lift and shape their right hand as to grasp a visually-pre- sented object.  Stimuli presentation was organized into trials in which pictures of the target objects  were shown on a computer screen for three seconds and were followed by an inter-stimulus pause), followed by an auditory cue that prompted the grasping movements.   In each trial, subjects were requested to grasp objects as if they were going to use them, and to place their hands in the resting position once the movement was over. | **kinematic hand synergies**: left M1; left SMA; left PMC; bilateral SPL; bilateral aIPS; | **Hand postural information, encoded through kinematic synergies, were represented in cortical areas devoted to hand motor control and successfully discriminated individual grasping movements, significantly outperforming alternative somatotopic or muscle-based models. Importantly, hand postural synergies were predicted by neural activation patterns within primary motor cortex.** | aIPS: anterior INtraParietal Sulcus; M1: Primary Motor cortex; PMC: PreMotot cortex; SMA: Supplementary Motor Area; SPL: Superior Parietal Lobiule; |
| Livne et al. (2022) | fMRI 3T | 15 healthy humans | mean 26.5 years | M:F=8:7 | right | whole brain | grip > baseline; extend > baseline; pinch> baseline; shake > baseline;  Grip, Extend, Pinch vs. Shake | grasp (visually guided) | precision grip | Partecipant underwent a block design hand-movement task in which they were instructed by a visual word cue to move their right hand repeatedly for 10 s in one of four movements followed by a variable rest period: -Grip, i.e finger flexion occuring during a grasping from a mid-opening hand position; -Extend, i.e i.e finger extension occuring during a heand releasing from a mid-opening hand position; -Pinch, i.e thumb-index opposition; -Shake, i.e adduction/abduction movement of the fexed wrist (not frequent and not ecological). | **grip / extend / pinch /shake > baseline**: Motor ROI | **The spatial pattern of activation for ecological movements (grip, extend, pinch) were more like each other than the control (Shake) movement.  Notably, the mean (variance) of the motor response was similar across all four movements and comparable across subjects hence unlikely to drive the classifcation.  Resting state patterns in human motor cortex were more like activation patterns for ecological movements than for the shake movement.  Diferences in rest-task similarity patterns between hand movements were not detected in area V1.** |  |
| Makuuchi et al. (2012) | fMRI 3T | 16 healthy humans | M mean age was 22.4 years (19-27); F mean age was 22.3 years (19-24). | M:F=9:6 (?) | right | whole brain | PRE1>baseline; POW1>baseline; POW1>PRE1; diff>same | pantomime grasp | precision grip; power grip (i.e whole hand grip squeezing); | Partecipants were instructed to mime right hand grasping movements as if the everyday use object projected onto the screen was close to their right hand (so that they did not need to reach for it); they were also advised to recall/imagine the object physical properties and how to use it. Subjects pantomimed grasping either by pinching with the thumb and index finger (precision grip) or by gripping it between the palm and all the fingers and thumb and squeezing (power grip): First Precision Grip Condition (PRE1): object presented for the first time; First Power Grip Condition (POW1) same ad for PRE1 but with power grip; Second Precision Grip Condition (PRE2same): object already presented; Second Power Grip Condition (POW2same): object already presented; Precision Grip With Preceding Power Grip Condition (PRE2diff): object already presented in power grip; Power Grip With Preceding Precision Grip Condition (POW2diff): object already presented in precision grip | **PRE1/POW1 > rest**:  PMv, AIP, M1, PMd, S1, IPL, pITG, SMA, thalamus and cerebellar hemispheres.  **POW2same-POW1/POW2 - diff-POW1/PRE2same-PRE1**:  decrease in in the PMv, DLPFC, and AIP along with other cortices lining the intraparietal sulcus   **PRE2diff - PRE**: increase in the PMv, DLPFC, and AIP along with other cortices lining the intraparietal sulcus  **diff > same (GRIP CHANGE)**:  The main effect was in the PMv, AIP and in (only left) pITG;  **same > diff**:  right LG;  **PRE > POW**:  left LOC, bilateral dorsal visual stream, and the right BG. | **The increased selection demands enhance the interaction between the AIP and pITG, and drive the converging causal influences from the AIP, pITG, and DLPFC to the PMv.   These results suggest that the dorsal and ventral visual areas interact in the pantomiming of grasping, while the PMv integrates the neural information of different regions to select the hand posture.** | AIP: anterior IntraParietal areas; BG: Basal Ganglia; DLPFC: dorsolateral PreFrontal cortex; IPL: Inferior Parietal Lobule; M1: Primary Motor cortex; PMd: PreMotor dorsal; PMv: PreMotor ventral; LG: lingual Gyrus; LOC: Lateral Occipital cortex; pITG: posterior Inferior Temporal Gyrus; S1: Primary Somatosensory cortex;SMA: Supplementary Motor Area; |
| Marneweck and Grafton (2020) | fMRI 3T | 48 healthy humans | median 21 years (18-37) | M:F=24:24 | right | whole brain | collinear grasp and lift left COM vs collinear grasp and lift right COM;  non-collinear grasp and lift left COM vs non-collinear grasp and lift right COM; | grasp (visually guided) | precision grip | subjects were asked to reach, precision grasp, and lift an inverted T-shaped object with a CoM on the left and right, respectively: the goal was to minimize tilting the object.  The extent to which grasp configuration and lift force varied between left and right CoM conditions was manipulated by instructing subjects to grasp the object collinearly (requiring a non-collinear force distribution) or to grasp the object non-collinearly (requiring a more symmetrical force distribution) | **CoM main effect**: IFG-pT, PMv, PMd, SMA; S1, OP1, AIP, SPL7; cerebellum.  **Grasp main effect**:  M1, SMA; PSC, OP1, AIP, IPL, SPL7; LOC.  **CoM x Grasp**:  IFG-pT, PMv | **that grasping and lift force are not represented by spatially separate functionally specialized regions, but by the same regions at differing time points.   The coordinated grasp to lift effort is shown to be under dorsolateral (PMv and AIP) more than dorsomedial control, and under SPL7, somatosensory PSC, ventral LOC and cerebellar control.** | AIP: Anterior IntraParietal area; IFG-pT: Inferior Frontal Gyrus - pars Triangularis; IPL: Inferior Parietal Lobule; LOC: lateral Occipital cortex; OP1: Parietal Operculum area 1; PMv: PreMotor ventral; PMd: PreMotor dorsal; S1: Primary Somatosensory Cortex; SMA: Supplementary Motor Area; SPL7: Superior Parietal Area 7; |
| Martin et al. (2011) | fMRI 1,5T | 14 healthy humans | mean 26 years (19–45) | M:F=7:7 | left | whole brain | hand left>baseline hand right>baseline tool left>baseline tool right>baseline | grasp and grasp with a tool (visually guided) | precision grip (here refferred to as a 2 fingered grip wight thumb and forefinger); power grip; | participants were trained to grasp an object that was presented in various orientations in a two fingered precision grip: this was undertaken with the hands (using the thumb and forefinger) and with a novel mechanical tool that 50 cm in length from handle to jaw which differed from the hand in two critical ways: 1) participants used a power grip to cause the two jaws of the tool to open or close in a “two- fingered” precision grip; 2) the opposition axis of the tool's prongs was rotated 90° relative to that of the hand.  Visual stimuli were computer-generated images of spheres with each side shaded to create the appearance of an indented fingerhold indicating where the sphere should be grasped; spheres were presented in 24 different orientations. Grasping task where completed with both the dominant left hand and nondominant right hand. | **all > rest**: vPMC; dPMC; aIPS and caudal cIPS extending to the POS and into the SPL; Lateral cerebellum; pre-SMA; left rMFG.  **2 (effector: hand vs. tool)× 2 (side: left vs. right) repeated measures ANOVA**: no main effect detected | **we found consistent bilateral increases in both the aIPS and vPMC activity regardless of the side (left, right) or effector (hand, tool) involved.  A direct comparison with right-handers revealed equivalent increases in left vPMC for both groups, suggesting that the functional contributions of this region to grip selection are unrelated to handedness.  By contrast, only left-handers showed significant increases in right vPMC activity, and aIPS activity for both groups was greater in the motor dominant hemisphere.** | aIPS: anterior IntraParietal Sulcus; cIPS: caudal IntraParietal Sulcus; dPMC: dorsal PreMotor cortex;  POS: ParietoOccipital Sulcus; pre-SMA: pre Supplementary Motor Area; rMFG rostral Middle Frontal Gyrusv; rMFG rostral Middle Frontal Gyrus; PMC: ventral PreMotor cortex; rMFG rostral Middle Frontal Gyrus; |
| Matsuda et al. (2009) | fMRI 1,5T | 23 healty humans | mean 28 ± 6.7 years (19-47) | M:F=17:7 | right | whole brain | symmetrical movements>rest reciprocal movements>rest alternating movements>rest | bimanual cordination grasp (visually guided) | N/A | The 3 movement conditions involved were: -symmetrical movements (SM), i.e simultaneous left and right hand grasp and release such that each 1 Hz is characterized by consecutive periods of both hands open and both hands closed;  -reciprocal movements (RM), i.e one hand grasps followed 1 Hz later by the other hand; -alternating movements (AM), i.e where one hand grasps then opens followed by the other hand in a repeat consecutive pattern.  All subjects performed under every movement pattern condition with order of first hand to grasp and order of task condition performed. determined at random. | **SM/RM/AM**:  bilateral SMC (greater for SM then RM and then AM), SMA (in particular significant in AM) and PMC (in particular right); right VPM, VPL and mammillary bodies (in RM significant activation in bilateral thalamus); bilaterral putamen and right lateral GP; bilateral parietal cortex (BA40)  **SM/AM**:  bilateral cerebellum (significant activation in right cerebellum) | **We found strong activations within bilateral SMC during all movement tasks  During all three bimanual coordination tasks, activation patterns in the SMC were surprisingly similar with greater activation in the right side than in the left.  This result implies that activities performed with the left hand by right handed subjects are more demanding with respect to cortical control needs, thus requiring more participation of the right SMC than the left SMC.** | BA: Broadmann Area GP: globus pallidus. PMC: PreMotor cortex; SMA: Supplementary Motor Area; SMC: SensoryMotor cortex; VPL: Ventro Postero Lateral nucleus of the thalamus; VPM: Ventro Postero Medial nucleus of the thalamus; |
| Matsumura et al. (1996) | PET | 8 healthy humans | 19-21 years | M:F=8:0 | right | whole brain | reach>baseline grasp>reach | grasp (visually guided) | precision grip | Five trasparent acrylic cilinders with different diameters where aligned in a circle 30cm in diameter. Partecipants performed the following tasks towards the signaled (by a visual cue) cylinder: -reaching with the index finger of his right hand; -grasping with thumb and index finger; -saccade toward the signaled cylinder. After the reaching and grasping task partecipants had to move their right hand on their chest with fingers open. | **reach (-saccade)**:  bilateral PreCG; bilateral IPS; cingulum;  **grasp > reach**:  left medial PFA,; left PreCG; bilateral PHA; bilateral cingulum; BG; cerebellum; | **The bilateral premotor area, posterior parietal area and prefrontal area might be key structures for the performing of grasping movements.** | PMA: PreCentral Gyrus; IPS: IntraParietal Sulcus; PFA: PreFrontal area; PHA: ParaHippocampal Area; BG: Basal Ganglia. |
| Michalowski et al. (2022) | fMRI 3T | 20 healthy humans | mean 24.7 years, (20-29) | M:F/10:10 | right | whole brain | GTD > cGTU;  dGTU, eGTU, and GTP > RAM (with the right and left hand);  planning dGTU > GTP;  planning eGTU > GTP;  planning GTP > eGTU;  planning dGTU > eGTU tasks;  left hand GTP > dGTU;  eGTU > dGTU; | pantomime grasp to use (GTU);  pantomime grasp to pass (GTP);  pantomime grasp to displace (GTD);  pantomimed tool use > "simulate animal movements";  "simulate animal movements" > pantomime tool use; | N/A | Stimuli consisted of photos of gaspable common objects (depending on their real sizes, half would require a precision grip, and half a power grip to manually handle them) in six different orientations.  BOLD fMRI signal was measured while participants planned and executed tool-oriented **pantomimed** actions in an event related design: (1) the GTU (grasp-to-use) task the to-be-seen object should be grasped in a way that would allow for its immediate use, without any further adjustment of the hand/wrist posture; there were two versions of the GTU task, a demanding (dGTU) and an easy one (eGTU), contingent on object rotation;  (2) the GTP (grasp-to-pass) task the object should be grasped in a simple or convenient way (i.e., without unnecessary hand rotation), with an intention to pass it to another person positioned in front of the participant;  (3) the RAM (reach-to-move) task, the object should be reached and simply moved (pushed) with the back of the hand.  Participants then had to pantomime the grasp identified by both the intention cue and the stimulus image as soon as the image of the object appeared. All the 20 participants were also tested twice (once per session) in a functional Tool Use Localizer (TUL) which served to identify brain areas associated with pantomiming the functional use of familiar tools: there were blocks of pantomimed tool use in responce of their image and and blocks of more abstract hand and finger movements in response to pictures of animals. The grasp-to-displace (GTD) localizer task served to identify brain areas associated with processing of structural tool information for grasping a tool to displace it or put it aside (a reference, structure-based condition).  The control grasp-to-use (cGTU) condition, participants were instructed to pantomime grasping the presented tool with an intention to subsequently use it according to its function. | **GTD > cGTU**: RH border of area PF and PFm, and at the intersection of areas PFm, PGi, and PGs.  **cGTU > GTD**: bilateral (LH>) LO3, MT, V4t, FST, PH, PHT through TE1p on the left, and mainly LO2, V4t, FST, PH, and PHT on the right.  Bilateral EVC, V1 through V4 on the right, and V1 and V2 on the left; left POS1, right intersection of 7PC, AIP, and area 2.  **pantomimed tool use > "simulate animal movements"**: left LOTC, IPL, IPS, SPL, lateral lIFC. Bilateral SMA and CMA. Right dPM, cingulate cortex and IFC.  **"simulate animal movements" > pantomime tool use**: V1–V3, on the left, and V1–V4, on the right, here also extending further to areas V8 and PIT, and even V4t/MT. Right tM and anterior FFC.  **dGTU, eGTU and GTP > RAM:** with the right hand: bilateral BA 4, 3a, 3b, and 1. Bilateral SMG, IPS extended from AIP through POS2, SPL cMTG bilateral V1 and V2; bilateral PMv and the SM/PMd vicinity. Bilateral AVI and FOP , lateral prefrontal, SMA complex and cingulate cortex.  **planning dGTU > GTP**: left BA 4, 3a, 3b, 1, and 2, anterior SPL PMd and supplementary motor cortex. Right aSMG and PSL. Left V1 and V2.  [≈ with left hand but strongly right-lateralizedand also more medial including the CMA]  **planning eGTU > GTP**:   V1 through V4, parieto-occipital/retrosplenial/posterior cingulate cortex  **planning GTP > eGTU**: with the right hand, left rMFG, PMd , cSPL/IPS, aIPS, SMG. WIth the left hand increases in neural activity were observed also in rMFG, PMd, cSPL/IPS, and finally SMG/IPS.  **planning dGTU > eGTU**:  -right hand: bilateral PMd/SM, bilateral posterior cluster from the somatosensory cortex to POS. -left hand: greater bilateral networks of dorsal and medial prefrontal/frontal, as well as parietal activity. In the PMd/SM vicinity, additional bilateral mid- to anterior cingulate cortex and clusters observed in frontal opercular regions; right rMFG.  **eGTU > dGTU**: left visual cortices | **regardless of the used hand, all major components of left-lateralized PRN were engaged in planning both functional and non-functional/structural grasps of tools more than in reaching actions with an intention to move or push tools as obstacles.  Yet, in contrasts with the latter task, there were also signifcant contributions from the right hemisphere counterparts of the identifed PRN nodes.** | aIPS, AIP/area 2: Anterior intraparietal sulcus; aSMG, PF/PFt/Pfop: anterior supramarginal gyrus; BA: Broadmann Area cMTG, PH/PHT: Caudal middle temporal gyrus. cSPL, VIP/MIP/7PL: Caudal superior parietal lobule; PMv, 6r/IFJp: ventral PreMotor cortex; rMFG: Rostral middle frontal gyrus; SM/PMd, 6mp, 6d: Supplementary motor / dorsal pre-motor cortex; |
| Monaco et al. 2011 | fMRI 3T | 11 healthy humans | mean 33 years | M:F/8:3 | right | whole brain | grasp>baseline reach>baseline look>baseline  grasp>reach>baseline grasp different>grasp same | reach/grasp/look (visually assisted) | precision grip | We had a combination of three TASKS (Grasp,Reach, and Look) and two rod ORIENTATIONS (Same and Different) that gave rise to a 3x2 factorial design. Therefore we had six experimental conditions: Grasp Same orientation (GS), Grasp Different orientation (GD), Reach Same orientation (RS), Reach Different orientation (RD), Look Same orientation (LS), and Look Different orientation (LD). | **grasp/reach/look > baseline**:  bilateral SPOC  **grasp > reach > look**:  bilateral aIPS   **GD > GS**:  bilateral SPOC and pIPS, left dPM  **Grasp and Reach > look**:  anterior portion of SPOC, anterior to the POS (aSPOC showed a clear involvement in wrist orientation for grasping, while pSPOC showed only a trend) | **We found reduced activation, that is, adaptation, in superior parieto-occipital cortex (SPOC) when the object was repeatedly grasped with the same orientation.  In contrast, there was no adaptation when reaching or looking at an object in the same orientation, suggesting that hand orientation, rather than object orientation, was the critical factor.** | aIPs: anterior IntraParietal Sulcus; dPM: dorsal PreMotor; pIPS: posterior IntraParietal Sulcus; SPOC: Superior Parieto-Occipital cortex; |
| Monaco et al. (2013) | fMRI 3T | 14 healthy humans | 20-42years | M:F=6:8 | right | whole brain | task (grasp or view) novel dimension > repeated dimension;  grasp novel dimension > repeated dimension;   view novel size > repeated size; | grasp visually guided | precision grip | Participants were required either to use their right hand to grasp the object or to passively view the object. In the grasping conditions, participants grasped the object by opposing the index finger and the thumb as in a precision grip.  The grasp-relevant dimension coincided with the bottom-up axis of the object, according to which the grip had to be scaled by using the same wrist posture in all trials. The grasp was performed without lifting the object. In the passive viewing conditions, participants were asked to attend the object without performing any action.  In all conditions, participants were required to attend to the location of the object without moving their eyes from the fixation point.  There was a combination of : -2 tasks, Grasp (G) and View (V),  -2 levels in the grasp-relevant dimension, repeated (rD) and novel (nD), and  -2 levels regarding object size, repeated (rS) and novel (nS), giving a 2 × 2 × 2 factorial design, which yielded 8 experimental conditions. | **task (grasp or view) novel dimension > repeated dimension**: left LOC, left SPOC.  **grasp novel dimension > repeated dimension**: left PMd.  **view novel size > repeated size**: left calcarine, left extrastriate. | **Areas in the parietal-frontal network (SPOC and dPM), as well as a ventral stream area (LOC), are clearly involved in processing objects’ grasp-relevant dimension rather than size.  We found a task-dependent effect in calcarine and extrastriate visual areas with adaptation to object size in viewing, but not in grasping conditions, and in dPM with adaptation to the grasp-relevant dimension for grasping, but not for viewing conditions.  In contrast, intermediate parietal and lateral occipito-temporal areas show adaptation to the grasp-relevant dimension regardless of the task, revealing a more flexible and encompassing encoding across tasks.** | LOC: lateral Occipital cortex; PMd: PreMotor dorsal; SPOC: Superior Parieto-occipital cortex |
| Monaco et al. 2015 | fMRI 3T | 11 healthy humans | mean 32 years | M:F/6:5 | right | whole brain | (1) Grasp DS SL vs. Grasp SS SL   (2) Grasp DS DL vs. Grasp SS DL   (3) Grasp SS DL vs. Grasp SS SL  (4) Grasp DS DL vs. Grasp DS SL  (5) Grasp DS DL vs. Grasp SS SL  +  same contrasts in the look conditions | reach-to-grasp | precision grip | We used a fMRI adaptation paradigm to investigate brain areas involved in processing object size and object location when relevant for reach-to-grasp movements.  On each trial, there was one of four possible combinations of two events:  (i) a repetition of both object size and location;  (ii)a repetition of object size in different locations;  (iii) a different object size in the same location; and  (iv) a different object size in different locations.   These four combinations were factorially crossed with two tasks (Grasp, Look), yielding eight conditions: -Grasp:Same Size:Same Location (Grasp SS SL);  -Grasp:Different Size:Same Location (Grasp DS SL);  -Grasp:Same Size:Different Location(Grasp SS DL);  -Grasp:Different Size:Different Location (Grasp DS DL);  -Look:Same Size:Same Location (Look SS SL);  -Look:Different Size:Same Location (Look DS SL);  -Look:Same Size:Different Location (Look SS DL);  -Look:Different Size:Different Location (Look DS DL).  The task (Grasp or Look) was cued with a verbal instruction at the beginning of each trial: in the ‘Grasp’ condition, participants used the dominant right hand to reach and grasp each of the two objects in the trial sequentially; in the ‘Look’ (passive viewing) control condition, participants observed the same trial sequences without performing any actions. | **conjunction analysis [(1) AND (2)]**: examining brain areas showing adaptation to object size regardless of object location.  Significant for grasp in left aIPS, significant for look in LH LOC/MT+  **(5)**:  significant for grasp and look in LH aIPS and LOC/MT+  **conjunction analysis [(3) AND (4)]**:  examining brain areas showing adaptation to object location regardless of size. No adaptation revealed  **conjunction analysis [(1) AND (3) AND (5)]**: examinig brain areas showing joint adaptation to object size and location. Significant in left PMd, SMA, LH SPOC, LH precuneus and left S1/M1.  The same analysis run on looking tasks did not reveal any significant activation.  ROI analysis:  aIPS: grasping in (1), (2) and (5) LOC/MT+: looking in (1) and (2), grasping and looking in (5) left SPOC: grasp in (1),(3) and (5). | **We found adaptation to object size for grasping movements in the left aIPS, in agreement with the idea that object size is processed separately from location.  In addition, the left SPOC, S1/M1, precuneus, PMd and SMA showed non-additive adaptation to both object size and location.   We propose different roles for the aIPS as compared with the SPOC, S1/M1, precuneus, PMd and SMA.  In particular, while the aIPS codes intrinsic object properties, which are relevant for hand preshaping and force scaling, area SPOC, S1/M1, precuneus, PMd and SMA code intrinsic as well as extrinsic object properties, both of which are relevant for digit positioning during grasping.** | aIPS: anterior Intra Parietal Sulcus; M1: Primary motor cortex; LOC/MT+: lateral Occipital cortex/visual motion area in the middle temporal gyrus; PMd: PreMotor dorsal; S1: Primary Somatosensory cortex; SMA: Supplementary Motor Area; SPOC: superior Parieto-Occipital cortex; |
| Monaco et al. (2017) | fMRI 4T | 18 healthy humans |  |  | right | whole brain | (Look at object > baseline) AND (touch object > baseline);  Look at object > touch object;  Touch object > look at object;  Immediate grasp > immediate reach;  haptic > visual exploration;  Immediate grasp > baseline) AND (immediate reach > baseline);  grasp > reach;  grasp > imagine;  grasp > stop;  reach > imagine;  reach > stop;   imagine > stop. | grasp visually guided; | precision grip | Participants first explored curvy planar objects visually or haptically and then performed delayed hand actions. Stimuli were located in the workspace of the hand below a fixation point such that the scene was viewed directly without mirrors. Grasping movements consisted of reaching to grasp the shape with a precision grip and reaching movements consisted of reaching to touch the shape with the knuckles, in the imagine grasping condition, participants were asked to imagine themselves grasping the shape without performing a real action. in stop trials, participants were required to abort the action, so no action was performed and there was no need to recall the stimulus. | **(Look at object > baseline) AND (touch object > baseline)**: LOtv.  **Look at object > touch object:** Cuneus.  **Touch object > look at object:** OP.  **Immediate grasp > immediate reach:** aIPS.  **haptic > visual exploration**: right LG.  **Immediate grasp > baseline) AND (immediate reach > baseline):** PMd.  **grasp > reach**: bilateral cuneus; right LG.  **grasp > imagine**: left LOtv dorsal; bilateral LOtv ventral; bilateral cuneus; right LG; bilateral OP; left aIPS; left PMd.  **grasp > stop**: left LOtv dorsal; left LOtv ventral; bilateral cuneus; right LG; bilateral OP; left aIPS; left PMd.  **reach > imagine:** left LOtv dorsal; right LG; left aIPS; left PMd.  **reach > stop:** left LOtv dorsal; left LOtv ventral; right LG, left OP; left aIPS; left PMd.  **imagine > stop**: left LOtv dorsal; left aIPS; left PMd. | **Exploration of unfamiliar shapes recruits the foveal cortex when shapes are explored haptically in the dark and retinotopic cortex (corresponding to the location of the object in the lower visual field) when shapes are viewed.  This suggests that object perception relies on different coordinate frames depending on the sensory modality used to explore the object. Interestingly, actions in the dark toward visually or haptically explored objects reactivate the EVC as well as LOtv, aIPS, and PMd regardless of the sensory modality initially used to explore the object. This suggests that the action plan accesses an abstract representation of the object that is stored in these areas.  Last, action imagery cannot entirely explain the reactivation for action in these areas.** | aIPS: anterior IntraParietal Sulcus; LG: Lingual Gyrus; Lotv: lateral Occipital tactile visual area; PMd: PreMotor dorsal; OP: Occipital Pole; |
| Nathan et al. (2012) | fMRI 1,5T | 18 healthy humans | mean 22.5 ± 3.1years | M:F/12:6 | right | whole brain | reach > relax; grasp > relax; reach and grasp > relax | grasp (visually guided) | N/A | A block design paradigm with 3 alternating ‘RELAX’ and ‘TASK’ states indicated by visual cues:  during the ‘RELAX’ state, subjects lay still in the scanner and during each ‘TASK’ state subjects performed 15 repetitions of the instructed movement task.  Three motor tasks were performed in three separate blocks: -reaching out and grasping a sponge ball; -reaching but not grasping the sponge ball; and  -only grasping of the sponge ball;  The use of the grasp only task was intended to mimic rhythmic finger tapping paradigms. Both the grasp-only and reaching-only tasks represent non-goal-oriented movements. | **reach/grasp only/reach and grasp > relax**:  activation intensity was much higher for the reach and grasp task in SMA, M1, S1 and parietal region.  The grasping only task had much higher activation in the visual cortex as compared to the reach only and the reach and grasp tasks. | **Neuromotor strategy for functional goal-oriented movements is different from rhythmic movements such as finger tapping or non-functional movements: this difference can be quantified and mapped using fMRI. There are some overlap with activation of movement execution however the cognitive component that mediates the specific movement is not just the linear combination of simple movements rather it is task and context specific.** | M1: Primary motor cortex; S1: Primary Somatosensory cortex; SMA: Supplementary Motor Area; |
| Pavlova et al. (2015) | fMRI 1,5T | 18 healthy humans | mean 33 years (20-49) | M:F/6:12 | right | whole brain | 1)Compression(T)–Rest(T)   2)Compression(noT)–Rest(noT);  3)Compression(T)–Compression(noT)  4)Compression(noT)–Compression(T). | sping compression task (where T and noT stand for conditions with and without tactile input, respectively) | precision grip | A spring compression task was employed under two sensory conditions: with and without local anesthesia to the thumb and index finger | **conjunction of (1) and (2):**  left SMC; left SMG; right IPS; bilateral insula; bilateral pallidum; right vPM;  **(3)**:  no significant difference  **(4)**: bilateral dPM | **all brain activity present during performance of a motor task with full access to sensory information is also present after digital anesthesia. This finding in combination with a compensatory activation of the right premotor cortex, can explain a relatively well-preserved fine motor control after acute tactile deafferentation** | dPM: dorsal PreMotor. IPS: IntraParietal Sulcus; SMC: SensoryMotor cortex; SMG: Supra Marginal Gyrus; vPM: ventral PreMotor; |
| Przybylski and Króliczak (2017) | fMRI 3T | 20 healthy humans | mean 22.7 years (19-24) | M/F:10/10 | right | whole brain | RH tool grasp planning > control grasp planning;  RH control grasp planning > tool grasp planning;  RH tool grasp VS control grasp;  LH tool grasp planning > control grasp planning;  LH control grasp planning > tool grasp planning;  RH tool grasp VS control grasp. | grasp planning; grasp | functional grip (i.e the best grip accordingly to the use of a specific tool) | Familiar tools and control objects with no obvious functions, were photographed and used as stimuli. Each was photograped and presented in three different orientations (0°, 135° and 225°).  Participants completed two separate sessions on consecutive days, using their dominant right and the non-dominant left hands, counterbalanced. There was no specific instruction about how the grasp planning should be performed. It was emphasized that hand movements and their rotations should be as precise as possible. Each trial consisted of an initial variable stimulus; subsequently, the “GRASP” cue, was shown to prompt participants to simulate preplanned grasps | **RH tool grasp planning > control grasp planning**:  left cMTG/LOc; left aSMG; left cSPL; left (>) and right aIPS; bilateral PMv; bilateral PMd; left adPreCun; bilateral SMA-complex; right aIC;  **RH control grasp planning > tool grasp planning**:  AG, bilateral ventral PreCun; bilateral mpMTG/STG;  **LH tool grasp planning > control grasp planning**:  left cMTG/LOc; left aSMG; left cSPL; left PMv; bilateral aIPS; bilateral PMd; bilateral SMA-complex; bilateral adPreCun; bilateral aIC; left rMFG; right sensorimotor cortices (S1-M1);  **LH control grasp planning > tool grasp planning**:  right AG; | **Planning functional grasps of tools (vs. non-tools) was associated with significant asymmetrical increases of activity in the temporo/occipital-parieto-frontal networks. The greater involvement of the left hemisphere PRN was particularly evident when hand movement kinematics (including wrist rotations) for grasping tools and non-tools were matched.  The networks engaged in the task for the dominant and non-dominant hand were virtually identical.  The differences in neural activity for the two object categories disappeared during grasp execution.** | adPreCun: antero-dorsal precuneus;aIPS: anterior Intra Parietal Sulcus; aIC: anterior Insular cortex; aSMG: anterio SupraMarginal Gyrus; cMTG/LOc: caudal Middle Temporal Gyrus/Lateral Occipital cortex; cSPL: caudal Superior Parietal Lobule; PMd: PreMotor dorsal; PMv: PreMotor ventral; SMA complex: supplementary/pre-supplementary motor area; |
| Ras et al. (2022) | fMRI | 20 healthy humans | mean 22.5 years | M:F=10:10 | right | whole brain | Grasping of complex > simple tools;  Grasping of tools > non-tools;  grasp planning of tools > non-tools;  use of tools > non-tools. | grasp planning; grasp to use; hMT; | functional grip | Real objects were used as stimuli in this project: they were both functional one-handed tools and non-tool graspable objects, and there were half tools associated with a low level of motor-to-mechanical transformations (simple tools), and half with a relatively high level of motor-to-mechanical transformations (complex tools); the to-be-handled object was made easy to recognize, grasp and use. All study volunteers undertook intensive training before the experiment proper and then they completed it with using their dominant right and the non-dominant left hands in a counterbalanced order. Participants open their eyes and an immediate verbal “Plan”command followed: in the case of tools, the command indicated planning a functional grasp in the context of the required subsequent action, regardless of whether simple or complex; in the case of non-tools, the plan command indicated preparation for grasping of a stick for subsequent pointing movements.  Then after the subsequent “Grasp” command and a variable delay interval participants had to grasp and then hold the presented object, no overt action was allowed until the subsequent “Use” command was delivered.  Following the use command, with either a tool or control object, participants perform a suitable manipulation on an action recipient until a “Go Back” cue was delivered. Each participant was also tested twice in a Tool Use Localizer (TUL), whose goal was to find a measure of brain activity limited primarily to tool use actions themselves, with little contribution from action planning and object grasping. | **Grasping of complex > simple tools**: left AIP; left 2/7PC; left MIP; left IP1/IP0; right PFt; right AIP/2; right MIP/IPS1/IP0.  **Grasping of tools > non-tools**: left AIP; left PFt; left a2/PFop; left V4v; left PH; right PFt; right AIP/PFt; right 6a; right 6ma; right V3v; right PIT.  **grasp planning of tools > non-tools**: left PFt; right PFt.  **use of tools > non-tools**: right PFt. | **demanding transformations of fnger movements into proper mechanical movements of functional parts of complex tools invoke signifcantly the right rather than left rostral IPL, and bilateral posterior-to-mid and left anterior intraparietal sulci. These fndings emerged during the functional grasp and tool-use programming phase.  The expected engagement of left IPL was partly revealed by traditional region-of-interest analyses, and further modeling/estimations at the hand-independent level.  Thus, our results point to a special role of right IPL in supporting sensory-motor spatial mechanisms which enable an efective control of fingers in skillful handling of complex tools.** | AIP: Intraparietal Sulcus, anterior Intraparietal Area; 2/7PC: Anterior Parietal Lobe, Superior Parietal Lobule; MIP: Intraparietal Sulcus, medial Intraparietal Area; IP1/IP0: Intraparietal Sulcus, posterior Intraparietal parcels; PFt: Inferior Parietal Lobule, anterior Supramarginal Gyrus; MIP/IPS1/IP0: Intraparietal Sulcus, mid-to-posterior Intraparietal parcels,. 2/PFop: anterior Parietal Cortex, Postcentral Gyrus; V4v: Occipital Cortex, Occipital Pole; PH: Occipital Cortex, Lateral Occipital Cortex; AIP/PFt: Intraparietal Sulcus, anterior Intraparietal Area; 6a: Frontal Cortex, Superior Frontal Sulcus; 6ma: Frontal Cortex, Superior Frontal Gyrus; V3v: Occipital Cortex, Occipital Pole; PIT: Occipital Cortex, ventro-lateral Occipital Cortex. |
| Rossit et al. (2013) | fMRI 3T | 10 healhty humans | 27 ± 5years | M:F=3:7 | right | whole brain | Grasp + Look > baseline;  Grasp > Look;  Grasping lowerVF > Grasping upperVF;  Look rightVF > Look leftVF;  Look leftVF > Look rightVF;  Grasp rightVF > Grasp leftVF;  Grasp leftVF > Grasp rightVF;  Grasping lowerVF > Grasping upperVF;  Right grasp > left grasp;  Left grasp > right grasp. | grasp visually guided | precision grip | participants were asked to maintain their gaze on one of four fixation LEDs positioned diagonally from the central object such that the object appeared in the upper left, upper right, lower left or lower right VF with respect to the fixation LED: -during grasping trials, participants employed a precision grip (using the index finger and thumb) to grasp the object along its longest axis (without lifting the object) with their right hand; -during Look trials, participants simply viewed the illuminated object while maintaining fixation. | **Grasp + Look > baseline**: Left SPOC; Right SPOC; Left aIPS; Right aIPS.  **Grasp > Look**: left SPOC; right SPOC; left aIPS; right aIPS.  **Grasping lowerVF > Grasping upperVF**: left SPOC; right SPOC;   **Look rightVF > Look leftVF**: left SPOC;  **Look leftVF > Look rightVF**: right aIPS.  **Grasp rightVF > Grasp leftVF**: left SPOC;  **Grasp leftVF > Grasp rightVF**: right SPOC; right aIPS.  **Grasping lowerVF > Grasping upperVF**: (Medially) Left SPOC; Left Precuneus; Left ant. cuneus  **Right grasp > left grasp**: Left PaHG; Left Cn.  **Left grasp > right grasp:** Right PaHG; Right Cn; Right MOG; Right SPOC;  Right aIPS; Right SPL; Right SFG; Left SMG; Left occ. pole | **SPOC and left precuneus have a lower VF preference for hand actions** | aIPS: anterior IntraParietal Sulcus; Cn: Cuneus; MOG: Middle Occipital Gyrus; PaHG: ParaHippocampal Gyrus; PreCn: PreCuneus; SFG: Superior Frontal Gyrus; SMG: SupraMarginal Gyrus; SPL: Superior Parietal Lobule; SPOC: Superio Parieto-Occipital cortex; |
| Stark and Zohary (2008) | fMRI 1,5T | 10 healthy humans | 21-31years | M:F= 10:0 | right | whole brain | grasp RH RF > rest grasp RH LF > rest grasp LH RF > rest grasp LH LF > rest | grasp visually guided | precision grip | To study the relative contribution of the visual and motor aspects in various regions along the visuomotor pathways, we applied a ROI analysis method, selecting voxels in anatomically restricted regions along the visuomotor pathway which had significant fMRI activation in all conditions relative to the rest period. The experiment included 4 conditions in a 2-by-2 design, manipulating the acting-hand and the visual-field in which the objects were placed. During separate blocks, subjects were required to use either their right (RH) or left hand (LH) in order to REACH and GRASP the handles of 3D tools located in the right (RF) or left periphery (LF), while maintaining central fixation. Tools were selected such that the handle and the functional part of the tool could be easily distinguished. During trest periods, subjects placed both arms on the table and maintained central fixation (rest). | **grasp RH > rest**:  left (controlateral) M1; left AIP; left MIP  **grasp LH > rest**:  right (controlateral) M1, AIP and MIP  **grasp RF > rest**:  left (controlateral) cIPS; SOG  **grasp LF > rest**:  right (controlateral) cIPS and SOG | **Quantitative analysis revealed 2 opposite visual and motor gradients along the posterior--anterior axis within the IPS: although the importance of the visual-field gradually diminished, the weight of the acting-hand became increasingly greater. Moreover, direct evidence for visuomotor interaction was found in all 3 IPS subregions, but not in occipital or frontal regions.  These findings support the hypothesis that the human IPS is comprised of subregions that have different properties, and that it is engaged in visuomotor transformations necessary for visually guided prehension.** | AIP: anterior IntraParietal area; cIPS: caudal IntraParietal sulcus; M1: Primary Motor cortex; MIP: medial Intraparietal Area; SOG: Superior Occipital Gyrus. |
| Sulpizio et al. (2020) | fMRI 3T | 25 healty humans | mean 26.5years | M:F/3:22 | right | whole brain | pantomime grasping > baseline;  imagine grasp > baseline;  pantomime grasping > imagine grasping. | pantomime grasping; imagine grasping. | N/A | photographs of graspable objects commonly used in everyday life were presented in central vision.  According to a written instruction, participants were required to perform a pantomime of an actual grasping by shaping the hand accordingly with the presented object (real condition) or to imagine that movement without shaping the hand (imagined condition). Partecipants were required to adjust their fingers/wrist configuration as appropriate as possible for the objects presented and to make an aperture/closure of their hand as much as they needed to grasp the object. In no case participants had to imagine using the object. | **pantomime grasping > baseline**:  hand territory of Left M1 and S1; bilateral PMd; bilateral SMA; bilateral CMA; bilateral PMv and iFg-PT; Bilateral pIPS with the adjoining SPL and IPL; bilateral SMG; bilateral dorsal surface of SPL from the caudalmost hV6Ad to the rostralmost hPEc; bilateral (but stronger in LH) aIPs.  **imagine grasp > baseline**:  left PMd; left SMA/CMA; left pCg; left iFg-PO and -PT; left aIPs; left pIPs; left SMG; left dorsal POs with partial hV6Ad; right (small foci) CMA; right PCg; right dorsal pIPS.  **pantomime grasping > imagine grasping**:  hand territory of Left M1 and S1; bilateral PMd; bilateral SMA and CMA; bilateral PMv and iFg-PT; bilateral SMg; bilateral dorsal surface of SPL from the caudalmost hV6Ad to the entire hPEc; right pIPS; bilateral (but stronger in LH) aIPs. | **involvement in the control of grasping movements of two specific cortical regions in the dorsomedial portion of the superior parietal lobule, hV6Ad and hPEc: -hV6Ad was activated by both real and imagined grasping;  -hPEc by real but not by imagined grasping. These results speak against the traditional notion of a medial-to-lateral segregation of reaching versus grasping information within the PPC and strengthen the idea that the human dorsomedial parietal cortex implements the whole complex pattern of visuomotor transformations required for object-oriented actions.  Our findings suggest that hV6Ad is particularly involved in implementing all the visuomotor transformations needed to create an abstract representation of the object-directed action, while hPEc is involved in implementing the sensorimotor transformations needed to actually perform that action.** | CMA: Cingulate Motor Area; hPEc: human area V6; hV6Ad: dorsal parte of human area v6; iFg-PT: Inferior Frontal Gyrus, pars-triangularis; IPL: Inferior Parietal Lobule;  M1: Primary Motor cortex; pIPS: posterior intraparietal sulcus; PMd: PreMotor dorsal; PMv: PreMotoro ventral; S1: Primary Somatosensory cortex; SMA: Supplementary Motor Area; SMG: supramarginal gyrus; SPL: Superior Parietal Lobule; |
| Vaillancourt et al. (2007) | fMRI 3t | 10 healthy humans | 21-35 years | M/F:6/4 | right | whole brain | grasp (hold) > rest;  grasp (sim pulse) > rest; grasp (diff pulses) > rest; grasp (sim pulse) > hold; grasp (diff pulse) > grasp (sim pulse); | held and pulsed (similar and different) power grasp | pinch grip | The current study was designed to examine whether specific nuclei of the basal ganglia in humans are involved in: a) producing the same series of force pulses, b) internally selecting different levels of force, or c) producing force pulses and selecting different force pulses. Subjects pinched the grip apparatus with their right index and middle finger opposing the right thumb to produce isometric force output, so an fMRI blocked-design was used while participant participants executed: -The HOLD task which required subjects to generate steady-state force at 15% of their MVC for the block (since they produced force to one known force level, they did not have to select amongst different force amplitude level); this task was performed internally-guided and externally-guided (with a visual cue). -The SIMILAR PULSE task which required subjects to generate a force pulse to 15% of MVC for 2 s, rest for 1 s, and repeat 10 times in the block (since the same force target was required on each force pulse subjects did not have to select which force level to produce on each force pulse). -Third, the DIFFERENT PULSE WHICH task required subjects to generate a series of force pulses to force levels that varied in amplitude within the block; target force levels were varied between 5% and 40% MVC with the average across the 10 targets per block equal to 15% MVC. Thus, the overall level of force was controlled as a potential confounding variable. During the externally-guided condition subjects generated pulses of force that were specified by the target on the screen and as such did not have to internally select which force level to produce; after 10 s rest, they then produced a series of different force pulses that they internally selected between 5% and 40% MVC. | **held and pulsed power grasp>rest**:  caudate, DLPFC, and ACC; group activation for GPi, posterior putamen, STN, SMA and M1; GPe, anterior putamen and pre-SMA  **similar pulsed power grasp>held power grasp**:  GPi, posterior putamen, STN, SMA and M1; GPe, anterior putamen, and pre-SMA  **variable pulsed power grasp>similar pulsed power grasp:** caudate, DLPFC, and ACC; GPe, anterior putamen, and pre-SMA | **Three main findings in the basal ganglia were identified: -Caudate nucleus had increased activation during the selection of force amplitude (it was the only basal ganglia region that displayed this specific pattern of activation).  -GPi had increased activation during the production of similar force pulses, but GPi did not increase activation during the selection of different force amplitude pulses. We also identified that the STN and posterior putamen increased activation during force pulse production and not force amplitude selection. -GPe had increased activation during force pulse production and further increased activation during force amplitude selection.** | ACC: Anterior Cingulate cortex; DLPFC: dorsolateral PreFrontal cortex; GPe: Globus Pallidus esternus; GPi: Globus Pallidus internus; M1: Primary Motor cortex; STN: SubThalamic Nuclues; SMA: Supplementary Motor Area; |
| Verhagen et al. (2008) | fMRI 3T | 19 healthy humans | mean 22 ± 2years | M:F=19:0 | right | whole brain | planning grasping and grasping > baseline;   grasping f(object slant);  grasping f(object slant) monocular > binocular. | grasp visually guided | precision grip | Subjects were asked to grasp a right rectangular prism along its longest dimension with their thumb and index finger; the target object could be rotated along the subject’s sagittal plane, in steps of 30° between 0 and 90° from the vertical plane.  Subjects’ vision was controlled by means of liquid crystal shutter goggles positioned in front of the left and the right eye independently controlled allowing the manipulafion of the type (i.e., monocular or binocular vision) and timing of visual information available to the subject: when they started the grasping task the shutters closed. | **planning grasping and grasping > baseline**: left superior PreCG; left superior PostCG; right and left cerebellum; right and left LG, right and left Cu; right and left MOG; right SPL; right and left SOG.   **grasping f(object slant)**: left occipitoparietal fissure, left MOG, left posterior inferior occipital gyrus, right MOG, left PMd.   **grasping f(object slant) monocular > binocular**: left LOtv; left PMv; left AIP. | **Dorsomedial parieto-frontal regions (V6A, PMd) were involved in the prehension movements regardless of viewing conditions.  In contrast, perceptual information processed in the ventral stream (LOtv) influenced the visuomotor process through dorsolateral parieto-frontal regions (AIP, PMv).** | AIP: anterior IntraParietal cortex; Cu: Cuneus; LG: Lingual Gyrus; LOtv: lateral occipital visual tactile area MOG: Middle Occipital Gyrus; PMd: PreMotor dorsal; PMv: PreMotor ventral; PreCG: PreCentral Gyrus; PostCG: PostCentral Gyrus; SPL: Superior Parietal Lobule; SOG: Superior Occipital Gyrus |
| Vingerhoets et al (2012) | fMRI 3T | 28 healthy humans | mean 21.5 years (19–26) | M:F=17:11 | right and left (14/14) | whole brain | unimanual tool dominant > unimanual control dominant,   bimanual tool dominant > bimanual control dominant,   bimanual > unimanual,   unimanual nondominant > unimanual dominant,  and all tools > all control conditions,  tools right dominant VS tools left dominant | pantomime grasp (viually guided) | N/A | The position of the object underlined with the right line indicated which hand had to be used to pantomime its use, if the red line was in between it indicated biamnual pantomiming. In unimanual control conditions, the volunteers were instructed to pantomime a rotating movement with the wrist while they imagined holding the egg with their fingers; in bimanual control conditions, they were asked to pantomime holding one egg in a central position, while rotating with the other egg around it.  Together, the instructions gave rise to eight different conditions: (1) unimanual right tool pantomime (UniTool-Right),  (2) unimanual left tool pantomime (UniToolLeft), (3) bimanual right dominant tool pantomime (BiTool-Right),  (4) bimanual left dominant tool pantomime (BiToolLeft),  (5) unimanual right control pantomime (Uni-ControlRight),  (6) unimanual left control pantomime (UniControlLeft),  (7) bimanual right dominant control pantomime (BiControlRight),  (8) bimanual left control pantomime (BiControlLeft). | **RH UniToolRight > UniControlRight**: PCG (Left [L]=Right [R]), vPM (L>>R), dlPFC (L>>R), posterior parietal (L > R), inferior temporal (L=R), and occipital (L=R) regions  **RH BiToolRight > BiControlRight**:  PCG (Left [L]=Right [R]), vPM (L>>R), dlPFC (L>>R), posterior parietal (L > R), inferior temporal (L=R), and occipital (L=R) regions, but with a more robust pattern of activation also spreading to the medial temporal region  **RH Bimanualt>Unimanual (corrected for controls)**:  SFG/MFG, MeFG; precuneus, IPS, occipital gyri, fusiform gyrus and MTG  **RH UniToolLeft > UniControlLeft = RH UniToolRight > UniControlRight  RH AllTool>AllControl:** PMC (L>>R), dsPFC (L>>R), meFC (L=R), posterior parietal (L>>R) and temporo-occipital regions (L=R)  **LH UniToolLeft > UniControlLeft**:  PCG (L=R), PMC (L=R), dlPFC (L>>R), posterior parietal (L>>R), inferior temporal (L=R), and occipital (L=R) regions  **LH BiToolLeft>BiControLeft**:  PCG (L=R), PMC (L=R), dlPFC (L>>R), posterior parietal (L>>R), inferior temporal (L=R), and occipital (L=R) regions, but with a more robust asymmetry  **LH Bimanualt>Unimanual (corrected for controls)**:  additional activation in SFG/MFG (L>R), IPS (L>R), occypital giry, cuneus and fusiform gyrus (R>L).   **LH UniToolRight>UniControlRight=LH UniToolLeft > UniControlLeft**   **LH AllTool>AllControl**:  almost identical pattern of left lateralized premotor, dorsolateral prefrontal, and posterior parietal activation than that of the right-handed participants  In comparison with left-handers, right-handers show additional activation in a region over the right precentral gyrus during bimanual compared with unimanual pantomiming | **A left hemispheric lateralization was observed in the right- and left-handed groupregardless of which hand(s) performed the task. Asymmetry was most marked in the DLPFC, PMC, SPL and IPL.  Unimanual pantomimes did not reveal any significant differences in asymmetric cerebral activation patterns between left- and right-handers.  Bimanual pantomimes showed increased left premotor and posterior parietal activation in left- and right-handers.  Lateralization indices (LI) of the 10% most active voxels in DLPFC, PMC, SPL, and IPL were calculated for each individual in a contrast that compared all tool versus all control conditions: left-handers showed a significantly reduced overall LI compared with right-handers, mainly due to diminished asymmetry in the IPL and SPL.   We conclude that the recollection and pantomiming of learned gestures recruits a similar left lateralized activation pattern in right and left-handed individuals. Handedness only influences the strength (not the side) of the lateralization, with left-handers showing a reduced degree of asymmetry that is most readily observed over the posterior parietal region.** | IPS: IntraParietal Sulcus; MeFG: Medial Frontal Gyrus; MFG: Middle Frontal Gyrus; MTG: Middle Temporal Gyrus PCG: PreCentral Gyrus; PFC: PreFrontal cortex; vPM: ventral PreMotor; SFG: Superior Frontal Gyrus; |
| Ward and Frackowiak (2013) | fMRI 2T | 26 healty humans | 21-80 years | M:F=17-9 | right | whole brain | dominant hand single handgrip > rest;  nondominant hand single handgrip > rest;  dominant hand > nondominant hand grasp;  nondominant hand > dominant hand grasp;  dominant hand > flipped dominant hand grasp;  nondominant hand > flipped nondominant hand grasp;  dominant handgrip > flipped non-dominant handgrip | grasp (visually guided) | dynamic isometric hand grip | Subjects performed a dynamic isometric hand grip task using dominant (right) and nondominant (left) hands in separate sessions, and in a randomized counterbalanced order using a magnetic resonance imaging compatible manipulandum consisting of two force transducers situated between two moulded plastic bars. Target forces during scanning were set at 10, 20, 40 and 60% of MVC for each subject | **dominant hand single handgrip > rest:** L. Central Sulcus, L. PostCG, L. PostCSulcus, bilateral PMd, bilateral caudal and rostral PMv, bilateral rostral cingulate sulcus, L. caudal cingulate sulcus, L. SMA, R. preSMA, bilateral Insula, bilateral SMG, R. IPS, L. superior parietal cortex, L. parietal and frontal operculi, L. STG, L. putamen, bilateral ventrolateral thalamus, L. red nucleus, bilateral cerebellum and vermis.  **nondominant hand single handgrip > rest:** R. Central Sulcus, R. PostCG, R. PostCS, bilateral PMd, R caudal and bilateral rostral PMv, R. caudal cingulate sulcus, R. SMA, R. preSMA, bilateral Insula, R SMG, bilateral IPS, R. parietal operculum, R. STG, bilateral putamen, R posterolateral thalamus, bilateral cerebellum and vermis.  **dominant hand > nondominant hand grasp**: L. Sensorimotor cortex, R Cerebellum (V),  R. Cerebellum (XI).  **nondominant hand > dominant hand grasp**: R. Sensorimotor cortex, L. Cerebellum (V).  **dominant hand > flipped dominant hand grasp**: L. Sensorimotor cortex; R. Cerebellum (V),  R. Cerebellum (XI), L. Frontal operculum.  **nondominant hand > flipped nondominant hand grasp**: R. Sensorimotor cortex, L. Cerebellum,  R. Angular gyrus.  **dominant handgrip > flipped non-dominant handgrip**: L. Parietal operculum, L. Insula cortex,  R. Intraparietal sulcus, R. Posterior inferior frontal gyrus. | **Handgrips most lateralized activations were in contralateral sensorimotor cortex and ipsilateral superior cerebellum.  Other activations were bilaterally distributed, including PMd and PMv, SMA, CMA, inferior parietal cortex and IPS, insula, cerebellar vermis and both inferior and superior cerebellar hemispheres.  Categorical comparisons of dominant and non-dominant hand grip demonstrated that the activation pattern generated differed only by the activation of contralateral sensorimotor cortex and ipsilateral superior cerebellum.  Conjunction analysis was performed between the effects of age and the effects of rest compared with hand grip: for both dominant and non-dominant hands, significant correlations were seen in ipsilateral M1, such that in younger subjects there was more likely to be a deactivation of ipsilateral M1 during hand grip compared with rest.** | CS: Cental Sulcus; IPS: IntraParietal Sulcus; FO: Frontal Operculum; PMd: PreMotor dorsal; PMv: PreMotor ventral; PO: Parietal Operculum; PostCG: PostCentral Gyrus; PostCS: PostCentral Sulcus; SMA: Supplementary Motor Area; SMG: SupraMarginal Gyrus; SMC: SensoryMotor cortex; STG: Superior Temporal Gyrus; |

Supplementary Table 2: **Main findings, subgroup “Grasping in the dark” (human primates):** *Abbreviations are reported in the main table.

| **Reference** | **Imaging technique** | **Sample (N)** | **Age (years)** | **Gender** | **Handedness** | **Target** | **Contrast (i.e. grasp > rest)** | **Category** | **Grasp type** | **Details** | **Cortical areas involved** | **Principle findings** | **Abbreviations** |
| --- | --- | --- | --- | --- | --- | --- | --- | --- | --- | --- | --- | --- | --- |
| Ariani et al (2018) | 4 T fMRI | 24 healthy humans | mean 28.21 years (18-38). | M:F=11:13 | Right | Whole Brain | Delayed reach-and-grasp or Delayed reach-and-touch > Baseline (Rest) | Grasp No Vision | N/A | To examine how movement representations evolve throughout different stages of planning, execution, and suppression, we compared three tasks in which movements were: (1) planned, withheld, and then executed (delayed go task);  (2) planned and immediately executed (non-delayed go task); or (3) planned, withheld, and then suppressed (delayed no-go task).  We used a slow event-related design with factors “movement type” (reach-to-touch; reach-to-grasp ) and “task” (delayed go; non-delayed go; delayed no-go). | **Planning delayed reach vs planning delayed grasp:** contralateral PMd, aIPS, SPL**.**   **Delayed reach/grasp vs Non-delayed reach/grasp**: bilateral M1, PMd, SMA, and aIPS**.**   **Delayed vs Non-delayed reach/grasp planning**: contralateral M1 | **During early stages of movement planning, representations of reach-to- touch and reach-to-grasp movements are similar to theones obtained during early stages of immediate movement  execution.  Significant decoding of hand movements during delayed planning in premotor (L-PMd) and parietal (L-aIPS) cortex.  Lack of within-condition decoding for planned movement types in visual or visuomotor areas.** | PMd: Pre-Motor dorsal; aIPS: anterior IntraParietal Sulcus; SPL: Superior Parietal Lobule; M1: primary motor cortex; SMA: Supplentary Motor area. |
| Buschbeck et al (2001) | fMRI 1.5T | 8 healthy humans | mean 28.5 years (22-33) | M:F=8:0 | right | whole brain | gentle hold VS rest;  normal hold VS rest; firm hold VS rest;  gentle VS normal hold (VS rest); firm VS normal hold (VS rest);  gentle VS firm hold (VS rest). | grasp no vision | precision grip | hold with precision grip a small object (weight 200 g) in the dominant right hand: -In one condition, they used their normal, automatically scaled grip force; -in a second condition the object was held gently the isometric grip force was maintained just above the critical level at which the object would have slipped; -in a third condition, the force was increased to hold the object with a more firm grip. | **gentle hold > rest**:  L. central sulcus M1/S1; L. precentral gyrus M1/PMD; L. inf. parietal cortex; L. intraparietal sulcus BA 7/40; L. inf. precentral gyrus PMV; Superior frontal gyrus SMA/CMA; R. inf. parietal cortex BA 40 44; R. intraparietal sulcus BA 7/40.  **normal hold > rest**: L. central sulcus M1/S1; L. intraparietal sulcus BA 7/40; R. inf. parietal cortex.  **firm hold > rest**: L. central sulcus M1/S1; L. precentral gyrus M1/PMD; L. inf. parietal cortex BA 40; L. intraparietal sulcus BA 7/40; L. inf. precentral gyrus PMV; R. inf. parietal cortex BA 40.  **gentle > normal hold**: L. central sulcus M1/S1; L. precentral gyrus M1/PMD; L. inf. precentral gyrus PMV; Superior frontal gyrus SMA; Cingulate sulcus CMA; L. inf. parietal cortex BA 40 (supramarginal gyrus).  **gentle > firm hold**: L. central sulcus M1/S1; Superior frontal gyrus SMA; Cingulate sulcus CMA;  L. inf. parietal cortex BA 40.  **firm > normal hold**: L. central sulcus M1/S1; L. precentral gyrus M1/PMD; L. inf. precentral gyrus PMV | **The supplementary and cingulate motor areas were significantly more active during the gentle force condition than during either of the other conditions in all subjects, despite weaker contractions of the hand muscles.  In addition, the left primary sensorimotor cortex, the ventral premotor cortex and the left posterior parietal cortex were more strongly activated during gentle than during normal grasping.** | M1: Primary Motor cortex; S1: Primary SomatoSensory cortex; PCG: PreCentral Gyrus; PMD: PreMotor dorsal; IPS: IntraParietal sulcus; PMV: PreMotor ventral; SFG: SuperioFrontal Gyrus; SMA: Supplementary Motor Area; CMA: Cingulate Motor Area. |
| Cavina-Pratesi et al (2018) | 4 T fMRI | 11 healthy humans | 24-37 years | M:F=7:4 | Right | Whole brain | all Grasps > all Reach/all Point;  PRECISION:  whole hand grasp precision + lifting > whole hand grasp coarse + lifting; whole hand grasp precision + lifting > 3 fingers grasp + lifting AND 3 fingers grasp + lifting > precision grasp + lifting.  LIFT: precision hand grasp + lifting > precision hand grasp;  All reach/point actions > passive viewing;  Reach-to-Touch and reach-to-point > Point-without-reach;  Point-with-reach AND Point-without-reach > Reach-to-touch;  point-without-reach actions > reach-to-touch AND reach-to-point; | Grasp No Vision / Pointing / Lifting | precision grip;  "3 fingers grip"; whole handgrip; | Nine different tasks were used such that subtraction logic would make possible to disentangle key cognitive components (indicated by letters and numbers):  -pointing with the index finger (P); arm transport (T); finger grasping (G) with two (2), three (3) or five (5) digits, either precisely (p) or coarsely (c); and object lifting (L). | **all Grasps > all Reach/all Point:** left S2; Medial Cerebellum; left Pulvinar; left PMd; left SMA; left PMv; left M1; left S1; left aIPS;  **whole hand grasp precision + lift > whole hand grasp coarse + lift**: left M1/S1; left aIPS;  **whole hand grasp precision + lift > 3 fingers grasp + lift AND 3 fingers grasp + lift > precision grasp + lift** left M1; **precision hand grasp + lift > precision hand grasp:** left M1; left aSPL; SMA; left PMd;  **All reach/point > passive view**: left aSPL; right aSPL; left aSPOC; left S2; Medial Cerebellum; left Pulvinar; left PMd; left SMA; left M1; left S1; left aIPS  **Reach-to-Touch and reach-to-point > Point-without-reach**: left aSPL;  **Point-with-reach AND Point-without-reach > Reach-to-touch**: right PMd; right S2; right SPOC; right aCu;  **point-without-reach actions > reach-to-touch AND reach-to-point**: right LOTC; right TPJ; left TPJ; | **These results clarify the roles of dorsal-stream regions such as aIPS, SPOC, aSPL and premotor cortex (PMv and PMd) in reaching, pointing and grasping.  Moreover, these data provide support for the idea that point-without-reach recruits regions within the ventral stream (LOTC) and another region that is anatomically situated between the two streams (TPJ).** | aCU: anterior Cuneus; aIPS: anterior IntraParietal Sulcus; aSPL: anterio Superio Parietal Lobule; aSPOC: anterior portion of the Superior Parieto-Occipital ortex;; LOTC: lateral Occipito-Temporal cortex; M1: Primary Motor cortex; PMd: dorsal PreMotor cortex; PMv: ventral PreMotor cortex; S1: Primary Somatosensory cortex; S2: Secondary Somatosensory cortex; SMA: Supplementary Motor Area; TPJ: temporo-parietal junction |
| Ehrsson et al (2000) | 1.5 T fMRI | 5 healthy humans | 21–27 years | M:F=5:0 | Right | Whole brain | Grasp (Precision grip) > Rest;  Grasp (Power grip) > Rest Grasp (power grip) > Grasp (precision grip); Grasp (precision grip) > Grasp (power grip) | Grasp No Vision | precision grip; power grip (i.e whole hand grip squeezing) | Partecipants performed two different grip tasks with the right hand and two matching rest tasks. We used functional magnetic resonance imaging to compare human brain activity during force production by the right hand when subjects used a precision grip and a power grip. During the precision-grip task, subjects applied fine grip forces between the tips of the index finger and the thumb. During the power grip task, subjects squeezed a cylindrical object using all digits in a palmar opposition grasp. | **Grasp (Precision grip) > Rest:** SMA; left CS (SMC, M1/S1); left PMd; left PMv; left lateral PO; left thalamus; left PosCS; left IFG; left AG; right IPS; right PMv; right MFS; right SMG; right IFS; right anterior insula; right MFG; right SPL;  **Grasp (Power grip) > Rest:** left PostCG (SMC, S1); left CS, upper anterior bank (SMC, M1/PMd); SMA; left lateral PO; left thalamus; Cingulate sulcus (CMAc); left PMv; right PostCS; right lateral PO;  **Grasp (power grip) > Grasp (precision grip):** left PostCG (SMC, S1); left CS, anterior upper bank (SMC, M1/PMd); left lateral PO; **Grasp (precision grip) > Grasp (power grip):** left IFS; left SMG; right IFS; right PMv; right IPS; right MFG; right CMAr; right SMG; | **The generation of grip forces by a precision grip between the index finger and thumb was associated with a different pattern of brain activity than that observed when making a power grip that engaged all digits.  The power-grip task was associated with stronger activity in the contralateral S1, M1 and PO, whereas the precision-grip task was associated with stronger activity in the right PMV, CMAr and the prefrontal and posterior parietal cortex bilaterally.  Many of the regions that were more active in the precision-grip task were right-sided (ipsilateral to the operating hand).** | AG: Angular Gyrus;CS: Central Sulcus; CMAr: Cingulate Motor Area rostral; CMAc: Cingulate Motor Area caudal; IFG: Inferior Frontal Gyrus; IFS: Inferior Frontal Sulcus; IPS: Inferior Parietal Sulcus; M1: Primary Motor cortex; MFS: Middle Frontsl Sulcus; PMd: PreMotor dorsal; PMv: PreMotor ventral; PO: Parietal Operculum; PostCS: PostCentral Sulcus; S1: Primary Somatosensory cortex; SMA: Supplementarry Motor Area; SMC: SensoryMotor cortex; SMG: SupraMarginal Gyrus; SPL: Superior Parietal Lobule; |
| Ehrsson et al (2001) | 1.5 T fMRI | 6 healthy humans | 21–28 years | M:F=6:0 | Right | Whole brain | Grasp > Rest;  Small > Large force; Large > Small force; | Grasp No Vision | precision grip | Here we use fMRI to examine the hypothesis that some of the areas of the brain associated with precision grips are more strongly engaged when subjects generate small grip forces than when they employ large grip forces.  Partecipants grasped a stationary object using a precision grip and employed a small force (3.8 N) (representative of the forces that are typically used when manipulating small objects with precision grips in everyday situations) or a large force (16.6 N) (representive of a somewhat excessive force compared with normal everyday usage). During the baseline condition (baseline), the subjects held the thumb and index finger in weak contact with the contact surfaces almost without applying any grip force. | **Grasp > Rest:**  leftl M1; left S1; bilateral PMv; bilateral BA44; bilateral PMd; bilateral SMA; bilateral CMA (both the caudal and the rostral); bilateral PO; bilateral anterior- and posterior parts of the bilateral intraparietal cortex; right SMG; left PreCu;   **Small > Large force**: left and right BA44; CMAr; right IPS; right PMv;  **Large > Small force:** left CS, M1; bilateral PO; | **Several sensory and motor related fronto-parietal areas were more strongly activated when a small precision grip force was applied to a stationary object than when a larger force was used.  This result suggests that the bilateral cortex lining the inferior part of the precentral sulcus (area 44/PMV), CMAr, and the cortex lining the right intraparietal sulcus are involved in the control of small fingertip forces in the range typically used in manipulation.** | BA: Boradmann Area; CMA: Cingulate Motor Areas; CS: Central Sulcus;  IPS: IntraParietal sulcus; M1: Primary Motor cortex; PMv: PreMotor ventral; PMd: PreMotor dorsal; PO: Parietal Operculum; PreCu: PreCuneus; S1: Primary Somatosensory cortex; SMA: Supplementary Motor Area; SMG: SupraMarginal Gyrus. |
| Ehrsson et al (2006) | fMRI 1.5T | 6 healthy humans | 20-32 years | M:F=6:0 | right | whole brain | loading VS baseline; unloading VS baseline; loading VS unloading; unloading VS loading. | grasp no vision | precision grip | The subjects used their right hand to perform a radial flexion of the wrist to lift and hold a nonmagnetic object between the tips of the thumb and the index finger. Participants lifted the object and held it still (they were instructed not to apply an excessive grip force or to make any movements when holding the object) during the holding, sudden changes in the weight of the object occurred (at random intervals so that anticipatory grip-force responses and learning-related effects).  Two different types of changes in the weight occurred (loading and unloading). | **loading VS baseline**: L. CMA; L. central sulcus (M1/S1);  L. PMD;  Med. cerebellum (vermis; Lobule VI); R. lat cerebellum (Lobule V).  **unloading VS baseline**: L. CMA; L. SMA; L. Pre-SMA; L. parietal operculum; L. SSA; L. Precuneus; R. sup. temporal g.; L. sup. temporal g.; L. sup. temporal s.; R. anterior cingulate cortex; R. ventral thalamus; L. ventral lateral thalamus; M. cerebellum (Lobule III).  **loading VS unloading**: L. central sulcus (M1/S1); L. postcentral g. (S1); L. central sulcus (M1/S1).  **unloading VS loading**: R. SSA | **First, we found increases in activity in the contralateral (left) primary motor cortex in response to loading, but not to unloading.   Second, we observed activity in the right lateral and medial cerebellum during the loading trials.  Third, unloading activated contralateral nonprimary sensorimotor areas rather than the primary sensorimotor cortex (M1 and S1), both the loading and the unloading events were associated with activity in the left cingulate motor area, the bilateral supplementary somatosensory area (SSA) was activated during unloading, but showed no response during loading.** | CMA: Cingulate Motor Area; M1: Primary Motor cortex; S1: Primary SomatoSensory cortex; PMD: PreMotor dorsal; SMA: Supplementary Motor Area; PO: Parietal Operculum; SSA: Supplementary SomatoSensory Area; ACC: Anterior Cingulate cortex; |
| Fabbri et al. (2014) | fMRI 4T | 16 healthy humans | 21-52years | M:F=9:7 | 15 righ, 1 left | whole brain | all movement types > baseline; | grasp no vision; | precision grip;  whole hand grip | During each trial, participants were presented with an arrow at the center of the screen and then using their right hand had to execute a center-out reach-to-grasp task on a device attached to their chest (visual feedback was not provided so as to exclude confounds) consisting of 5 half-spheres placed at five equidistant positions on a virtual circle and a 6th positioned at the center. At the beginning of each trial, participants positioned their index finger on the central half-sphere and then executed center-outmovements in one of the five possible directions using one of the three different movement types as soon as the arrow appeared on the screen, and to then move back to the start position: reach direction was indicated by the orientation of the arrow presented on the screen, while the type of movement was specified by its color. | **all movement types > baseline**: left M1; left PMd; left PMv; left S1; left SPLr; left SPLa; left SPLp; left aIPS; left SMA. | **We found overlapping representations for both the reach and grasp components in PMDi, PMv,M1, S1, SPLa, and aIPS.  Moreover, we observed trends for an interaction between the reach and grasp components in PMv, M1, and aIPS, and SPLa, tentatively suggesting that these areas might be involved in the combination of the reach and grasp component.** | aIPS: anterior IntraParietal Sulcus; M1: Primary Motor cotrex; PMd: PreMotor Dorsal; PMv: PreMotor ventral; S1: Primary SomatoSensory cortex; SMA: Supplementary Motor Area; SPLa: Superior Parietal Lobule, anterior portion; SPLp: Superior Parietal Lobule, posterior; SPLr: Superior Parietal Lobule, rostral portion; |
| Fiehler et al (2011) | 1.5 T fMRI | 21 healthy humans | mean 23.6 ± 3.0 years | M:F=6:15 | Right | Whole brain | Grasp (No delay) > Rest; Grasp (Delay) > Rest:  Grasp (No delay) > Grasp (Delay) | Grasp No Vision | precision grip | Participants perfromed grasping movements with the right hand and a precision grip from the home key to the stimulus (rotating the forearm around the elbow and the hand around thewrist) to three-dimensional objects of different size and orientation.  Reaching-to-grasp movements were performed without visual feedback either immediately after object presentation (no delay) or after a variable delay of 2–12 s (delay). | **Encoding**:  bilateral CalcG; bilateral LinG; left FusG; left M1   **Memory delay**:  right inferior parietal cortex (IPC: hIP2, PFm in supramarginal gyrus and PGa in angular gyrus); right STS/MTG; left IFG.   **Grasp (No delay) > Rest**:  left M1; left PMCd, left S1; bilateral SMA proper and pre-SMA (at a lower threshold the sensorimotor activation cluster spreads into the PPC covering aIPS); right cerebellum.  **Grasp (Delay) > Rest:**  very similar activation pattern to immediate grasping: no significant differences from this contrast. | **The right inferior parietal cortex demonstrated sustained neural activity throughout the delay, which overlapped with activity observed during encoding of the grasp target. Immediate and delayed grasping activated similar motor-related brain areas and showed no differential activity.  The results suggest that the right inferior parietal cortex plays an important functional role in working memory maintenance of grasp-related information. Moreover, our findings confirm the assumption that brain areas engaged in maintaining information are also involved in encoding the same information, and thus extend previous findings on working memory function of the PPC in saccadic behavior to reach-to-grasp movements.** | aIPS: anterior IntraParietal Sulcus. hIP2: human IntraParietal Area 2; IFG: Inferior Frontal Gyrus; M1: Primary Motor cortex; MTG: Middle Temporal Gyrus; PFm: parietal area F, part m; PGa: parietal area G, anterior; PMCd: PreMotor cortex dorsal; PPC: Posterior Parietal cortex; S1: Primary Somatosensory cortex; SMA: SUpplementary Motr Area; STS. Superiot Temporal sulcus; VLPFC: VentoLateral PreFrontal Cortex; |
| Gatti and Rocca et al. (2016) | fMRI 3T | 29 healthy humans | mean 23.4years (19.1-29.9) | M:F=14:15 | right | whole brain | EXECUTION:  -SM vs CM, -SM vs FM; -CM vs SM; -CM vs FM; -FM vs SM; -FM vs CM  OBSERVATION: -SM vs CM; -SM vs FM; -CM vs SM; -CM vs FM; -FM vs SM; -FM vs CM;  OBSERVATION AND EXECUTION: -SM vs CM; -SM vs FM; -CM vs SM; -CM vs FM; -FM vs SM; -FM vs CM.  OE vs O;  OE vs E. | grasp no vision | whole hand grip | Subjects had to execution (E) of a motor task, the observation (O) of a video showing the same task performed by another person and the simultaneous observation and execution (OE) of the task were obtained from three groups of healthy subjects (15 subjects per group) randomized to perform:  -a simple motor (SM) task (subjects had to open and close the last four fingers of the right hand, maintaining the thumb abducted and the forearm in an intermediate position between pronation and supination);  -a complex motor (CM) task (subjects had to open, abduct, adduct and close the last four fingers of the right hand maintaining thumb and forearm in the intermediate position); -a finalistic motor (FM) task (subjects performed three finalistic movements using 3 objects positioned on the wooden table: each participant had to raise the little cup and to leave it on the wooden table, open and close the clothes peg and turn the button clockwise).  Participants performed the tasks in three different conditions: -execution (E); -observation (O), subjects watched a video showing another person performing the requested task with the right hand; -observation and execution (OE). | **EXECUTION**:  **-SM vs CM**:  L Inferior frontal gyrus, pars triangularis; L Inferior frontal gyrus, pars orbitalis.  **-SM vs FM:** L Precuneus; L Inferior frontal gyrus; L Middle occipital gyrus; R Superior frontal gyrus; L Anterior cingulate gyrus.  **-CM vs SM:** R Middle temporal gyrus; L Supplementary motor area; L Superior temporal gyrus.  **-CM vs FM**:  R Precuneus; L Precuneus; L Precental gyrus; L Cuneus; L Lingual gyrus; R Cuneus;  L Supplementary motor area; R Middle frontal gyrus; L Middle frontal gyrus  **-FM vs SM**: L Middle occipital gyrus; L Inferior occipital gyrus; R Middle occipital gyrus; L Superior frontal gyrus; R Thalamus; L Inferior parietal lobule.  -**FM vs CM**: L Middle occipital gyrus; L Inferior occipital gyrus; R Middle occipital gyrus; L Superior frontal gyrus; L Inferior frontal gyrus, pars orbitalis; Vermis; L Middle frontal gyrus;  L Inferior parietal lobule   **OBSERVATION**: -**SM vs CM**: R Inferior frontal gyrus, pars orbitalis; R Insula.  -**SM vs FM**: R Superior occipital gyrus; R Lingual gyrus.   -**CM vs SM**: L Superior parietal lobule; L Cerebellum.  -**CM vs FM**: R Medial occipital gyrus; R Calcarine cortex.  -**FM vs SM**: R Superior temporal gyrus; R Middle temporal gyrus; L Superior temporal gyrus; L Precental gyrus; L Superior parietal lobule; L Inferior parietal lobule; R Cerebellum; L Supplementary motor area; L Cerebellum; R Precental gyrus.  -**FM vs CM:** L Superior temporal gyrus; L Hippocampus; R Superior temporal gyrus; R Inferior frontal gyrus, pars triangularis; L Putamen;  L Precental gyrusM; L Inferior frontal gyrus, pars triangularis.   **OBSERVATION AND EXECUTION**: -**SM vs FM**: R Lingual gyrus; R Superior occipital gyrus.  -**CM vs SM**: R Middle cingulum; L Superior parietal lobule.  -**CM vs FM**: R Middle occipital gyrus; R Precuneus.  -**FM vs SM**: R Superior temporal gyrus; R Middle temporal gyrus; L Superior temporal gyrus;  L Precental gyrus; L Postcentral gyrus;  L Inferior parietal lobule; R Cerebellum; L Supplementary motor area; R Precental gyrus; Vermis; L Middle temporal gyrus.  -**FM vs CM**: L Hippocampus; R Superior temporal gyrus; R Inferior frontal gyrus, pars triangularis;  L Putamen; L Precental gyrus;  L Inferior frontal gyrus, pars triangularis.   **OE vs O**: L Postcentral gyrus; L Supplementary motor area; L Putamen; R Cerebellum; Vermis;  L Cerebellum; L Thalamus; R Precentral gyrus; R Superior temporal pole; Insula; L Cuneus.   **OE vs E**: R Inferior occipital gyrus; R Inferior temporal gyrus; L Middle occipital gyrus; L Middle temporal gyrus; L Superior temporal gyrus; R Superior temporal gyrus; R Middle temporal gyrus; L Inferior parietal lobule; L Middle frontal gyrus; L Superior frontal gyrus. | **The use of a familiar, intransitive task, might represent an option for modulation of MNS recruitment in patients for whom a finalistic movement might be unfeasible.** | IFG: Inferior Frontal gyrus MOG: Middle Occipital Gyrus; SFG: Superior Frontal Gyrus; ACC: Anterior Cingulate Gyrus; MTG: Middle Temporal Gyrus; SMA: Supplementary Motor Area; STG: Superior Temporal Gyrus; LG: Lingual Gyrus; PCG: PreCentral Gyrus; PreCu: preCuneus; MFG: Middle Frontal Gyrus; IPL: Inferior Pariuetal Lobule; |
| Holstrom et al. (2014) | fMRI 1.5T | 16 healthy humans | mean 32 ± 4years (26-42) | M:F=16:0 | right | whole brain | active conditions > baseline;  High instability > Low instability;  high force magnitude > low force magnitude. | grasp no vision | pinch grip | Subjects were instructed to compress each spring while preventing them from buckling that is a strenght and dexterity test (S-D): -the strength requirement is defined as the pinch force necessary to compress the spring to solid length; -the dexterity requirement is defined as the ability to compress the spring without buckling.  Springs were constructed to correspond to two levels (high–low) of (1) force magnitude and (2) instability index and four springs covered four distinct strength–dexterity combinations:  -HF/ LI (high force/low instability),  -LF/LI (low force/low instability),  -HF/HI (high force/high instability),  LF/HI (low force/high instability). | **active conditions > baseline**: left SFG (Left SMA); Right MFG (PMd); Right insula; Left PreCG (PMv); Left PostCG (S1); Right PostCG (S1); Left SMG; Left MOG; Right cerebellum (VI); Right cerebellum (IV–V); Right cerebellum (VIII); Cerebellar vermis.  **high instability > low instability**: left M1; rght M1; left IPS; right S1; left cerebellum (VI); right cerebellum (VI).  **high force magnitude > low force magnitude:** left M1; left CS; right medial temporal pole; right LG; right CalcG; left cerebellum (VIII); right cerebellum (IV-V); right cerebellum (IX); right cerebellum (crus 1); cerebellar vermis. | **Tasks that mainly require generation and control of fingertip force magnitude (normal to the spring end surface) predominantly engage a different part of the classical grasping network than tasks that also require precise control of fingertip force direction.  Increased brain activity associated with the contrast high force–low force magnitude was seen in the contralateral primary motor regions, central sulcus, and bilateral cerebellum consistent with the idea that M1 is more involved in force magnitude control, and less so in the control of the direction of the force vector.** | CalcG: Calcarine Gyrus; CS: Central Sulcus; IPS: IntraParietal Sulcus; LG: Lingual Gyrus; M1: Primary Motor cortex; MFG: Middle Frontal Gyrus; MOG: Middle Occipital Gyrus; PostCG: PostCentral Gyrus; PreCG: PreCentral Gyrus; PMd: PreMotor dorsal; PMv: PreMotor ventral; S1: Primary Somatosensory cortex; SFG: Superior Frontal Gyrus; SMA: Supplementary Motor Area; SMG: SupraMarginal Gyrus; |
| Hong et al (2011) | 1.5 T fMRI | 19 healthy humans | mean 28.9 years (25-33) | M:F=17:2 | Right (both right and left hand) | Whole brain | Grasp > Rest; | Grasp No Vision | N/A | Participants were examined supine with their eyes closed, secured firmly with forearms in supination.  A repetitive alternating cycle of control and stimulation with grasp-release hand movements at a metronome-guided frequency of 1 Hz was performed for the motor task.  Probabilistic tractography was used to analuze DTI data that were collected using fMRI activation induced by the motor task | **Grasp > Rest:**  left PMC; left SPL; left IPS; left SMG; left SMA; left MTG; left VPL nucleus of the thalamus; left posterior one-third of the putamen; left IFG-pO; left STG; left IFG-pT; left AG; right M1; left cerebellum hemisphere | **The neural network related to hand movements consisted of many brain areas in addition to those previously identified for motor planning and execution. Brain areas connected to the SM1 were the premotor cortex , superior parietal lobule, intraparietal sulcus, supramarginal gyrus, supplementary motor area, thalamus, putamen, pars opercularis, pars triangularis, angular gyrus, and cerebellum in the same hemisphere and the contralateral primary motor cortex in the opposite hemisphere.** | AG: Angular Gyrus; IFG-pO: Inferior Frontal Gyrus- Pars Opercularis; IFG-pT: Inferior Frontal Gyrus- Pars Triangularis; IPS: Inferior Parietal sulcus; M1 : Primary Motor cortex; MTG: Middle Temporal Gyrus; PMC: PreMotor cortex; SM1: Primary Sensory-Motor cortex; SMA: Supplementary Motr Area; SMG: SupraMarginal Gyrus;  SPL: Superior Parietal Lobule; STG: Superior Temporal Gyrus; VPL: Ventro PosteroLateral. |
| Kim et al (2021) | 3 T fMRI | 25 healthy humans | mean 24 ± 4 years (19–29) | M:F=9:16 | Right | Whole brain | Grasp (Objects) > Grasp (No Object) | Grasp No Vision | N/A | An experimenter placed the stimulus on the palm of the participants’ right hand with the thin side oriented toward the thumb and participants grasped each stimulus once for 1 s and released it with a grasping force as constantly as possible for every stimulus.  Immediately after participants released the stimulus, the experimenter removed it from participants’ hands and placed the next stimulus after an inter-stimulus interval.  A total of five stimuli (four hardness levels plus one sham stimulus) were presented.  The objective of the fMRI experiment was to identify brain regions related to tactile hardness perception and neural activities representing the hardness intensity during active exploration of presented objects. | **Positive correlation:** right pIns;  **Negative correlation:** right pCerebellum; | **We found a significant positive correlation between hardness intensity and neural responses in the posterior insula in the right hemisphere (rpIns) and we found a significant negative correlation in the right posterior lobe of the cerebellum (rpCerebellum).** | pCerebellum: posterior Cerebellum; pIns: posterior Insula; |
| Kim et al (2011) | 1.5 T fMRI | 14 healthy humans | mean 25 years (21-29) | M:F=7:7 | Right (Bilateral hands) | Whole brain | Clapping > Grasp-release > Sequencial opposition | Grasp No Vision | N/A | All subjects were examined in a supine position with their eyes closed, and were secured firmly with an immobilizing frame.  Three different motor tasks were performed using a block paradigm: -clapping; -grasp-release movements of the bilateral hand;  -sequential opposition of fingers. | **Clapping > Grasping > Sequential opposition**:  M1, PMC, SMA | **In M1, clapping showed the most significant activation, followed by sequential opposition and grasp-release movements. By contrast, the PMC was most activated by sequential opposition and then clapping and grasp-release movements, in order. As for the SMA, clapping evoked the most significant activation, followed by grasp-release and sequential opposition movements.** | M1: Primary Motor cortex; PMC: PreMotor cortex; SMA: Supplementary Motor Area. |
| Kurniawan et al (2010) | fMRI 3T | 17 (of 18) healty humans | 27 ± 3years | M:F=13:5 | right | whole brain | Choice to Grip > Choice to Hold;  GripLE > GripHE;  HoldHE > HoldLE;  Persistence > HoldLE;  reward X choice to grip > choice to hold. | grasp no vision | power grip | The choice task was split in choice and execute periods: -in choice periods, participants made a long series of consecutive choices between an effortful gripping option and a holding option (indicated by visual stimuli). Levels of effort and reward were manipulated in effortful gripping, and presented a fixed minimum reward with zero effort for the holding option; -in execute periods, participants executed a proportion of their selected options from the preceding choice period, by gripping (or simply holding) a hand device at the corresponding effort level to receive the corresponding reward amount. | **Choice to Grip > Choice to Hold**:  SFG, Midlle parietal lobe.  **GripLE > GripHE**: L. Putamen, L. S1, L. M1, R. Cingulate motor area, R. SMA, L. SMG, L. MTG.  **HoldHE > HoldLE**: Mid-brain (ventral thalamus).  **Persistence > HoldLE**: ACC, Posterior MTG.  **reward X choice to grip > choice to hold**: ITG; SMA | **we found that the putamen was more active during anticipation of low relative to high effort, a finding that argues against traditional notions of the putamen being solely involved in pure motoric aspects of movement execution and points to a role in a higher order aspect of action valuation.** | ACC: Anterior Cingulate cortex; CMA: Cingulate Motor area; ITG: Inferior Temporal Gyrus; M1: Primary Motor cortex; MTG: Middle Temporal Gyrus; S1: Primary Somatosensory cortex; SFG: Superior Frontal Gyrus; SMA: Supplementary Motor Area; SMG: SupraMarginal Gyrus; |
| Marangon et al. (2016) | fMRI 3T | 10 healthy humans | mean 28.1 years | M:F=6:4 | right | whole brain | explore>rest; explore complex>explore simple; grasp planning>rest; grasp>rest; grasp>reach; grasp> NoGo | grasping no vision | explorative grip (i.e haptical exploration of un unseen object to understand its physical properties) | All the manual tasks in the main study of this project were performed with the dominant right hand. Presented objects varied in shape and size and were arranged in random sets of 8 including two control discs. Participants were firstly asked to explore the presented object and then to move the hand back; next they had to plan a grasping movement of the just explored object; finally either a grasp (50%, with an execution of the planned grasp), reach (25%, with a reach and touch with knuckles) or wait (25%, witha NoGo) command could have been given; finally a rest phase would conclude the task. | **Haptic object exploration > rest**:  Right pre-SMA; Right CMA, Left aIPS, Left aSMG, Left S2, Left PMd, Left PMv, Left rMFG, Posterior CalcarineSulcus, Left Lateral TOC, Left pMTG, Left Precuneus.  **Haptic exploration of (complex > simple) objects**:  Right rSPG, Right aIPS, Right aSMG, Right S2.  **Grasp planning > rest**:  Anterior Calcarine Sulcus, Posterior Calcarine Sulcus, Left amIPS, Left PMd, Left V4v, Left ppFusG, Left pMTG.  **Haptically-guided grasp > rest**: Right pre-SMA; Right CMA, Left aIPS, Left aSMG, Left S2, Left PMd, Left PMv, Left rMFG, Posterior CalcarineSulcus, Left LOTC, Left pMTG, Left Precuneus, Left pSTG, Right pSTG, Left cIPS, Left SPOC.  **Haptically-guided Grasp > Reach**:  Left aIPS, Right rSPG, Right aSMG, Right PMv.  **Haptically-guided Grasp > NoGo**:  Left PO, Right Parietal Operculum, Left PMv, Right PMv, Left SMA, Right SMA, Left pre-SMA. | **The most critical aspects of task performance are controlled by the dorsal-stream regions, with much greater—in fact exclusive— contribution of the right hemisphere to the haptic processing of object shape (or its exact graspable dimension), and the bilateral involvement of the parieto-frontal networks, including left aIPS, in the control of the haptically-guided grasping.  Furthermore, two different kinds of signal processing for grasp performance have been associated with the dorso-dorsal vs. ventro-dorsal parieto-frontal networks (thus forming a dorso-ventral gradient), with the emphasis on representing grasp-relevant features of the unseen targets, and monitoring of grasp kinematics, respectively. Of course, intermediate areas such as aIPS show sensitivity to both object shape and the required grip kinematics.** | aIPS: anterior IntraParietal Sulcus; amIPS: anterior-to-mid Intraparietal Sulcus; aSMG: anterior SupraMarginal Gyrus; cIPS: caudal Intraparietal Sulcus;  CMA: Cingulate Motor Areas; LOTC: Lateral Occipito-Temporal Cortex; pFusG: posterior Fusiform Gyrus; PMd: dorsal Premotor Cortex, PMv: ventral Premoto rCortex; PO: Parietal Operculum; pMTG: posterior Middle Temporal Gyrus; preSMA: pre Supplementary Motor Area; pSTG: posterior Superior Temporal Gyrus; S2: Secondary Somatosensory cortex; rMFG: rostral Middle Frontal Gyrus, Lateral  rSPG: Right rostral Superior Parietal Gyrus; SPOC: Superior Parieto-Occipital Cortex; V4V: ventral Visual Area4; |
| Milner et al. (2007) | fMRI 1.5T | 17 healthy humans | N/A | N/A | N/A | whole brain | simple > rest;  complex > rest;  complex > simple. | grasp no vision | pinch grip (thumb and two or three fingers) | There were three conditions, corresponding to three tasks performed while lying in the supine position with the right hand: Tasks consisted of squeezing a foam ball (simple), balancing a weighted flexible ruler (complex) and resting; partecipants used a pinch grip between the thumb and two or three fingers. | **Simple > rest:** Left M1, Right anterior cerebellum (lobule V/VI).  **Complex > rest:** Left M1, Right anterior cerebellum (lobule V/VI), Right posterior cerebellum (lobule VIII), Left thalamus, Left anterior insula, Left S1 (area 3), Left S2.  **Complex > simple:** Left S1 (area 2); Right Brodmann area 40. | **cerebellum is differentially activated to control grasp while holding an object with complex dynamics compared to one with simple dynamics; because of the similarity in the activation of M1 under the simple and complex conditions, it is unlikely that the neural representation of the task dynamics is localized in M1.  Somatosensory association areas such as S2, Brodmann area 40 and insula, which were selectively activated under the complex condition, are likely involved in the integration of somatosensory information in the context of physical attributes of the object, which in this case might include its flexibility.** | M1: Primary Motor cortex; S1: Primary SomatoSensory cortex; S2: Secondari SomatoSenory cortex; |
| Neely et al. (2013) | fMRI 3T | 17 healthy humans | 21-33years | M:F=9:8 | right | whole brain | Static > Dynamic;  Dynamic > Static;  Dynamic > Static. | grasp no vision | precision grip | A visual display contained two horizontal bars:  -a fixed target bar representing 15% of the participant’s MVC (maximum voluntary contraction); -a movable white force bar. Participants were instructed to produce force when the target bar changed from red to green and received online visual feedback about their force via the white, moveable horizontal bar. In the static task, participants produced isometric force to 15% of their MVC for 30 seconds. In the dynamic task, participants produced force to 15% of their MVC for 2 seconds. Each 2-second force pulse was separated by 1 second of rest: a series of 10 force pulses plus rest were completed to achieve 30 seconds of precision grip force. | **Static > Dynamic**: R IPL; R PMv; R DLPFC; R DLPFC.  **Dynamic > Static**:  SMA; L SPL; L Fusiform gyrus; L V3; L Lobule VI.  **Dynamic > Static**:  L M1; L S1; L PMd; SMA; L V5/MT; L LO;  R Lobule VI; R Crus II. | **There were widespread similarities between static and dynamic force control; however, distinct differences were also observed.  In particular, the production of static force was associated with a right-lateralized cortical network, whereas the production of dynamic force was associated with a mostly left-lateralized cortical network.** | DLPFC: DorsoLateral PreFrontal cortex; Fus: Fusiform gyrus; IPL: Inferior Parietal Lobule; LO: Lateral Occipital cortex; M1: Primary Motor cotex; PMd: PreMotor dorsal; PMv: PreMotor ventral; S1: Primary Somatosensory cortex; SMA: Supplementary Motor Area; V3: Third Visual cortex; V5/MT: extrastriate V5/Middle Temporal area; |
| Renzi et al (2013) | fMRI 1.5T | 15 healthy humans | 28 ± 9 years | M:F=10:5 | right | whole brain | no vision grasp > vision grasp;  vision FD grasp > vison ND grasp;  no vsion ND grasp > no vision FD grasp. | grasp visually guided | precision grip; whole hand grasp. | Before each block, participants were orally instructed about the size of the stimulus they were going to deal with (small or big spheres); then, on a auditory cue, they were asked to reach and grasp with their right dominant hand either using a precision grip (PG) or a whole hand grasp (WHG). The spheres to be grasped were placed at two different distances: ‘near’ (ND) or ‘far’ (FD) from the hand (in order to reach almost the maximal arm extension).  In the proprioceptive feedback condition, volunteers performed the task with their eyes closed.  Nine of the volunteers also performed a session with visual feedback on a different day after the proprioceptive feedback condition session and had their head tilted at an angle of approximately 30° in order to let them see the stimuli directly. | **no vision grasp > vision grasp**: right lingual gyrus; righ fusiform (including the parahippocampal area; BA 19/36/37);  **vision FD grasp > vison ND grasp**: right SPL; left SPL; right dPM; right postcentral/IPL.  **no vsion ND grasp > no vision FD grasp**: right postcentral/IPL. | **More dorsal cortical areas may be involved in spatial remapping processes that integrate visual information for action when an object is located farther away.  On the other hand, the activity in a more ventral anterior parietal area may reflect remapping processes for the boundaries of peripersonal space and thus encode peripersonal space flexibly depending on sensory feedback.** |  |
|  |  | 9 | 24 ± 3years | M:F=7:2 |  |  |  | grasp no vision |  |  |  |  |  |
| Schmuelof and Zohary (2006) | fMRI 1,5T | 14 healthy humans | 25-35 years | M/F:7/7 | right and left | whole brain | grasp watching lateralized > control (scrambled) | passive grasp (i.e grasp watching) | N/A | Each epoch of right-hand footage (in the left and right periphery) was composed of 10 clips of a right hand approaching from the right, grasping and releasing an object; Left- hand clips were generated by a “flip horizontal” operation on the “right- hand” clips.  In all clips, the objects remained stationary and did not move throughout the grasping movements. In the control “scramble” conditions, a 12 s object-manipulation epoch was decomposed to frames, every frame was spatially scrambled.  The experiment was performed using a block design format, the conditions involved viewing the following: 1) object manipulations by the right hand, shown in the left visual field;  (2) object manipulation by the right hand, shown in the right visual field;  (3) object manipulation by the left hand, shown in the left visual field;  (4) object manipulation by the left hand, shown in the right visual field;  (5) spatially scrambled version of the object manipulation clips, shown in the left visual field; and  (6) spatially scrambled version of the object-manipulation clips, shown in the right visual field. | **2 x 2 factoral design identity of the seen [acting hand (left or right) and location of the viewed objects (and grasping hand) in the peripheral  visual field (right or left of the fixation point)] grasp watching**:  Contralateral visual-field preference in both hemispheres, throughout the LOTC, as well as in dorsal areas, in the transverse occipital sulcus.  In addition, in the right hemisphere, we found a significant preference for left-field clips in the cIPS adn SPL. Cortical areas that displayed a significant hand-identity effect, (i.e., preference to video clips showing the contralateral hand) are mainly found between the superior bank of the aIPS and PostCG (AIP) | **fMRI activation in the occipital cortex and in the caudal sections of the parietal cortex was specific to the visual-field location of the clips. In contrast, the principal factor determining the response in anterior intraparietal cortex was the identity of the observed hand.  Furthermore, these “hand-specific” parietal areas also showed contralateral hand specificity during self action (i.e., object manipulation) without visual feedback.  A similar selectivity for the identity of the observed hand was seen when using a ROI analysis focusing on individually defined visuomotor voxels withinthe parietal cortex.  This dual visuomotor grasping representation lends further evidence for the existence of a mirror system in humans and suggests that the anterior intraparietal cortex is involved in the specific motor simulation of hand actions.** | AIP: anterior IntraParietal area; cIPS: caudal IntraParietal Sulcus; LOTC: lateral Occipito-Temporal cortex; SPL: Superior Parietal Lobule; |
|  |  | 9 healty humans | N/A | N/A | right and left | whole brain | (grasp and grasp watching) - ankle flexion > control | passive grasp | N/A | the visuomotor areas were identified as those voxels that show both (1) selective activation during object-manipulation observation (as in part1) and (2) hand-specific motor activation (defined by contrasting the activation during object manipulation using the contralateral hand with the activation during contralateral ankle movement). | **(grasp and grasp watching) - ankle flexion**: preference for the contralateral hand in the left AIP and to both the contralateral hand and visual field in the right AIP although the effect of hand identity was more pronounced |  |  |
| Spraker et al. (2009) | fMRI 3T | 12 healthy humans | 19-34years | M:F=5:7 | right | whole brain | FR > rest; FG > rest; FG > FR; FR > FG. | grasp no vision | pinch grip (thumb and middle finger) | The paradigm required subjects to use a pinch grip (thumb and middle finger) with their right hand to produce force to a target magnitude. The task consisted of alternating rest and task blocks with rest blocks positioned at the beginning and end of the sequence. During the task blocks, the subjects completed the following sequence: a 30-s precisely controlled force generation (FG) condition, an 18-s rest condition, and a 30-s precisely controlled force relaxation (FR) condition. | **FR > rest**:  L M1; caudate bilaterally  **FG > rest:** L M1; caudate bilaterally  **FG > FR**: L M1; caudate bilaterally;  **FR > FG**:  R DLPFC; bilateral ACC. | **Controlled force generation activates a similar network to precisely controlled force relaxation, yet there are also fundamental differences in how the brain regulates these 2 force tasks: the caudate and M1 have greater activity during precisely controlled force generation compared with precisely controlled force relaxation, whereas the DLPFC has greater activity during force relaxation compared with force generation.  The current study also found greater deactivation in bilateral ACC for precisely controlled force relaxation than precisely controlled force generation.** | ACC: Anterior Cingulate cortex; DLPFC: DorsoLateral PreFrontal cortex; M1: Primary Motor cortex; |
| Styrkowiec et al. (2019) | fMRI 3T | 21 healthy humans | mean 22.5 years (20-29) | M:F=10:11 | right | whole brain | Haptic exploration of tools > exploration of nontool objects;  Planning functional grasp of tool / non-tool > baseline;  Planning grasp of non-tool vs planning functional grasp of tool;  Haptically guided functional grasping of tools > grasping of nontools;  Haptically guided functional grasping of rotated > non-rotated tools;  haptically based tool use actions > arbitrary hand movements with non-tool objects in hand; | Grasp No Vision | functional grip | The experimental stimuli consisted of three-dimensional objects: tools and controls; their orientations were counterbalanced. Participants could not see their stimuli, similarly, the participants did not have any visual feedback of their hands. Throughout the whole run, a participant acted only with one hand while the other one remained static.  Each trial consisted of: 1)exploration, i.e participants raise their hands, reach to the location of the stimulus and haptically explore it in order to recognize whether it was a tool or a control and determine its orientation; 2)return, i.e returnign back to starting position; 3)plan, i.e plan a grasping movement (in the case of non-tools the task was to plan the simplest possible reach and grasp movement, while in the case of tools the task was planning a grasping movement that would allow for tool use action consistent with its function); 4)grasp, i.e executing the planned action; 5)return. | **Haptic exploration of tools > control objects**:  left LOC (inferior division); left MTG (temporooccipital part); left and right SPL; left and right LOC (superior division); Left SPOC; Left S1; left and right aIPS; Left MFG (posterior part); left and right AG; left and right aSMG;  left and right PMd; left and right PMv; left MFG; left and right ParaCG; left and right CG; left PreCu; left FusG.   **Planning functional grasp of tool / non-tool > baseline:** left SPL; left IPL; left MTG; small area il left MFG;  **Planning grasp of non-tool vs planning functional grasp of tool**: weaker inhibition of motor-related areas, as well as bilateral regions often linked to the default mode network;  **Haptically guided grasping of tools > non-tool objects**: left aIPS; left SPL(anterior part); left S1; left PMd; left and right Juxtapositional Cortex; left SMA; left MFG; left and right IC; left LOC (superior division); left PreCU; left Intracalcarine Cortex; left ParaCG; right SPOC; right SMG; right PMv; right PMd; right Juxtapositional Cortex; right Intracalcarine Cortex.  **Haptically guided functional grasping of rotated > non-rotated tools**.  bilateral thalamus and putamen, aIC, pIC, aSPL, PMd, M1, SMA, pre-SMA, CMA (whereas dorso-dorsal stream and parietal operculum showed right-lateralized advantage with extention to S2).  **haptically based tool use actions > arbitrary hand movements with non-tool objects in hand**: LOtv area; cMTG;  **ROI analyses to show the direction and amplitude of (%) signal changes in all main study condition relative to resting baseline with the emphasis on task, object, and hand effects**: cMTG, SMG and rMFG showed significantly greater activity in grasp planning (at least when compared to exploration); however, similarly to the whole-brain analysis, none of them showed greater activity for tools.  Nearly the opposite pattern of results was observed in PMv and PMd, wherein planning-related activity was significantly lower as compared to exploration and grasping; only in the latter two tasks the activity for tools was significantly higher than for control objects.  SPL showed the least expected pattern of activity and it was such that there was a gradual decrease of activity from exploration through grasping, with the latter being significantly lower than exploration- and planning-related activity. A significant decrease of activity from exploration to grasp planning and execution was also observed in PMd; yet, consistently with earlier ROIs (except for grasping in cMTG), both during exploration and grasping tool-related activity was significantly higher than the one for control objects. | **haptic exploration and haptically guided grasping of tools engages the majority of areas linked to the temporo-parieto-frontal praxis representation network, which is typically associated with processing of information related to tool-oriented actions. While this may sound obvious, the involvement of PRN in tool exploration and grasping has never been demonstrated in a haptic domai: this in turn suggests that PRN operates on modality-independent inputs.  During haptic exploration some preliminary planning of subsequent target-directed responses takes place and, indeed, it resembles visual affordance processing although achieved more gradually in a haptic domain.   A functional grasp of a tool is based on integration of conceptualtool knowledge with processing of structural tool features which starts as early as during haptic exploration, and is performed and then maintained by the anterior supramarginal gyrus: our study convincingly shows that this inferior parietal area can efficiently guide the control of a functional grasp even in the absence of continuous inputs from the temporal regions involved in conceptual processing of tools.** | aIC: anterior Insular Cortex; aIPS: anterior IntraParietal sulcus; aSMG: anterior SupraMarginal Gyrus; aSPL: anterior Superior Parietal Lobule; CG: Cingulate Gyrus; CMA: Cingulate Motor Area; cMTG: caudal Middle Temporal Gyrus; FusG: Fusiform Gyrus; M1: Primary Motor cortex; MFG: Miffle Frontal Gyrus; OPS: Occipito-Parietal Sulcus; pIC: posterior Insular Cortex; ParaCG: ParaCingulate Gyrus; PMd: PreMotor dorsal; PMv: PreMotor ventral; pRCG: posterior divisions of rostral cingulate gyrus; preCun: preCuneus; preSMA: pre Supplementary Motor Area; rMFG: rostral Middle Frontal Gyrus; SPL: Superior Parietal Lobule; supAG: superior divisions of Angular Gyrus; S1: Primary Somatosensory cortex; S2: Secondary Somatosensory cortex; SPOC: Superior Parieto-Occipital cortex; |
|  |  |  |  |  |  |  | tactile min > baseline; tactle max > baseline; | dexterous manipulation task (no vision) |  |  |  |  |  |
| Turella et al. (2020) | fMRI 4T | 21 healthy humans | mean 29.2 years | M/F:10/11 | right | whole brain | grasping > baseline | Grasp No Vision | precision grip; whole hand grip | Participants were scanned while performing a motor task, which consisted in executing non-visually guided grasping actions on an object:  a 2×2×2 factorial design was used with the factors: “wrist orientation” (no rotation, 0°, vs. rotated wrist, 90°), “effector” (LH vs. RH), and “action” (precision grip, PG, vs whole hand grip, WH).  Participants were instructed to grasp two objects of different sizes, specifically a precision grip toward the small central block, using the thumb and index finger, and a whole-hand grip using their entire hand on the large lateral side of the object. Participants had to perform the grasping action by simply touching the object,without manipulating or moving it. | **grasping > baseline**:  bilateral PMC, M1, and S1; bilateral PPC, posterior temporal cortex,PO; bilateral insula;  **Concrete action representation**:  bilateral M1, PMv, only right PMd;bilateral aIPS, mIPS and pIPS (non dependent of used hand, but left PMd active only for right hand)  **Effector-dependent goal encoding**:  left M1 and PMv when right hand used;  right M1 and PMd when left hand used, right PMv when right hand used; left aIPS, mIPS and pIPS (non dependent of used hand);  right aIPS, mIPS when left hand is used, pIPS when right hand is used.  **Effector-Independent goal encoding**:  left aIPS and pIPS; right aIPS | **We investigated a hierarchical organization consisting of three levels of abstraction:  1) “concrete action” encoding;  2) “effector-dependent goal” encoding (invariant to wrist orientation); and  3) “effector-independent goal” encoding (invariant to effector and wrist orientation).   We found that motor cortices hosted joint encoding of concrete actions and of effector-dependent goals, while the parietal lobe housed a convergence of all three representations, comprising action goals within and across effectors.  The left lateral occipito-temporal cortex showed effector-independent goal encoding, but no convergence across the three levels of representation.   Our results support a hierarchical organization of action encoding, shedding light on the neural substrates supporting the extraordinary f lexibility of human hand behavior.** | aIPS: anterior IntraParietal Sulcus; M1: Primary Motor cortex; mIPS: medial Intra Parietal Sulcus; pIPS: posterior Intra Parietal Sulcus. PPC: Posterior Parietal cortex; PO: parietal operculum; PMv: PreMotor ventral; PMd: PreMotor dorsal; S1: Primary Somatosensory cortex; |

Supplementary Table 3: **Main findings, subgroup “Hand manipulation task” (human primates):** *Abbreviations are reported in the main table.

| **Reference** | **Imaging technique** | **Sample (N)** | **Age (years)** | **Gender** | **Handedness** | **Target** | **Contrast (i.e. grasp > rest)** | **Category** | **Grasp type** | **Details** | **Cortical areas involved** | **Principle findings** | **Abbreviations** |
| --- | --- | --- | --- | --- | --- | --- | --- | --- | --- | --- | --- | --- | --- |
| Binkofski et al (1999) | 1.5 T fMRI | 12 healthy humans | 25-35 years | M:F=12:0 | Right and Left | Whole brain | Hand manipulation > Rest;   Hand manipulation (complex object) > Hand manipulation (sphere) | Hand manipulation (no vision) | N/A | The experiment was designed to assess the cortical areas involved in manipulation of complex objects.  -In the condition (a) the activation phase consisted of a continuous manipulation of complex plastic objects, while the baseline phase consisted of rest, during which no motor activity was required.  -In the second condition (b) the activation phase was the same as in the first one, while the baseline consisted of continuous indifferent manipulation of a sphere. | **Manipulation of complex objects > rest:**  S1, M1, dPMC, vPMC, SMA proper, the cingulate motor cortex (mCing, BA 24), S2, SPL and AIP.  (S1, M1 and mCing were activated contralateral to the manipulating hand whilst the dPMC and vPMC and the parietal areas AIP, S2 and SP were activated bilaterally).   **Manipulation of complex objects > manipulation of a sphere**: bilateral activation of vPMC (BA 44), AIP, S2, SPL and IPL; additional activation for left hand manipulation was pSPL. | **During manipulation of complex three-dimensional objects there are, in humans, selective activations of vPMC (BA 44), an area located in the anterior part of the lateral bank of the intraparietal sulcus (area AIP, BA 40) and of S2.** | AIP: anterior intraparietal area;  dPMC: dorsal premotor cortex; mCing: cingulate motor cortex;  M1: primary motor cortex;  S1: primary somatosensory area;  S2: secondary somatosensory area;  SMA: supplementary motor area;  SPL: superior parietal lobule;   vPMC: ventral premotor cortex. |
| Binkofski et al (1999) | 1.5 T fMRI | 5 healthy humans | 25–35 years | N/A | Right | Whole Brain | Hand manipulation (complex object) > Hand manipulation (sphere) | Hand manipulation | N/A | Different meaningless complex plastic objects (could not be recognized by means of tactile exploration) were used as stimuli.  Subjects were asked to manipulate them carefully with their right hand with the aim of exploring each of their single features (active condition); each active condition was followed by a baseline condition during which subjects were asked to perform the manipulation of a sphere, which was rotated in their hands. | **complex object > sphere hand manipulation**: left (+) and right vPMC; AIP; bilateral opercular part of the IPL (including S2); | **in humans there is a parieto-frontal circuit related to hand-object manipulation.  The cortical sites forming it are an area located anteriorly in the intraparietal  sulcus and a sector in the region of Broca’s area (area 44).  The hand-manipulation circuit appears to be linked to S2, which most likely provides the somatosensory information concerning the manipulated objects.** | AIP: anterior IntraParietal area; IPL: Inferior Parietal Lobule; vPMC: ventral PreMotor cortex; S2: Secondary Somatosensory cortex; |
| Park et al. (2008) | fMRI 1,5T | 11 healthy humans | mean 23.5 years (21-26) | N/A | right | whole brain | simple hMT > rest (right anf left handed); complex > simple; | grasp (visually guided); hMT (visually guided); | N/A | At first, two sessions of each motor task were executed in random order using one hand, and the same process was then performed in two sessions with the other hand. Each task condition, during which subjects were asked to perform the ball rotation task or the grasp movement, was followed by a rest condition. | **Simple hMT RH**:  left M1/S1, SMA and right (ipsilateral) Qua.  **Complex hMT RH**:  left M1/S1, left PMC and right (ipsilateral) Qua; ipsilateral PMC, S1 and left (controlateral) Qua.  **Simple hMT LH**:  right M1/S1, SMA, and left (ipsi) Qua.  **Complex hMT LH**:  region comprising from the right (controlateral) PMC across the CS to PPC; left (ipsi) S1 and left PMC, and right (contra) Qua. | **We showed significantly stronger ipsilateral hemisphere and bilateral cerebellar activities during execution of complex movement compared to simple hand movement.  The ipsilateral motor cortex can be activated when the task is very complex and when the contralateral hemisphere is not well-trained in the execution of the motor task** | CS: Central Sulcus; M1: Primary Motor cortex; S1: Primary Somatosensory cortex; SMA: Supplmentary Motr Area; PMC: PreMotor cortex; PPC: Posterior Parietal cortex; Qua: quadrangular lobule of the anterior lobe of the cerebellum. |
| Talati et al. (2005) | fMRI 1,5T | 10 healthy humans | mean 27 years | M:F=5:5 | right | whole brain | tactile min > baseline; tactlle max > baseline; | dexterous manipulation task (visually guided) | pinch grip (here reffered to as a thumb, indle and middle finger grip) | A spring compression task (three point pinch) was performed under four combinations of visual and tactile sensory input:  (a) eyes closed, no visual input, and smooth (minimal) tactile sensation,  (b) eyes closed and rough (maximal) tactile sensation,  (c) eyes open, watching hand, and smooth tactile sensation, or  (d) eyes open, watching hand, with rough tactile sensation | Eight anatomical regions met the criteria for an Active Area (5 continu- ous voxels, p< .0005, during all four conditions by subject): MFG (BA6); cingulate gyrus (BA 24); M1 (BA 4); S1 (BA 3, 1, 2); IPL (BA 40); MOG (BA 19); IOG (BA 18); cerebellum.  Differences in average voxel counts were observed in the MOG, IOG and IPL when the eyes were open and closed, and in M1 when the tactile input was either a smooth or rough surface. Otherwise, there were no significant differences between the sensory condi- tions for the regions. **dextrous motor task**:  SMA; cingulum; leftM1; leftS1; IPL; MOG; IOG; right cerebellum | **Performance of the dexterous task remained constant in all conditions.  Variations in the two levels of visual input resulted in modulation of activity in the middle and inferior OGG and IPL, and variation in the two levels of tactile input during the task resulted in modulation of activity in M1.  Although significantly active in all conditions, cingulate gyrus, MFG, PostCG and cerebellum activities were not modulated by levels of either visual or somatosensory input, and no interaction effects were observed.   Together, these data indicate that a fine-tuned motor task guided by varying visual and tactile information engages a distributed and integrated neural complex consisting of control and executive functions and regions that process dynamic sensory information related to guidance functions.** | IPL: Inferior Parietal Lobule; IOG: Inferior Occipital Gyrus. M1: Primary Motor cortex; MOG: Middle Occipital Gyrus; S1: Primary Somatosensosry cortex; SMA: Supplementary Motor Area; |
|  |  |  |  |  |  |  | tactile min > baseline; tactle max > baseline; | dexterous manipulation task (no vision) |  |  |  |  |  |
